# ‘It’s life threatening, it’s not life limiting but it’s life threatening’ – Dyadic framework analysis of adolescent and parent adjustment to a type 1 diabetes diagnosis

**DOI:** 10.1177/13591053231216700

**Published:** 2023-12-30

**Authors:** Andrea Habenicht, Elayne Ahern, Declan Cody, Vincent McDarby, Sharon Houghton

**Affiliations:** 1 University of Limerick, Ireland; 2 Children’s Health Ireland at Crumlin, Ireland

**Keywords:** adjustment, adolescent-parent relationship, diagnosis, dyad, type 1 diabetes

## Abstract

Type 1 diabetes-management can be considered an adolescent-parent collaboration. Given particular adolescent adherence challenges, it is integral that adolescent-parent dyadic relationships are investigated. Therefore, this study aimed to explore dyads’ adjustment to type 1 diabetes, while examining the congruence/dissimilarity within these dyads. Semi-structured interviews were conducted with 10 dyads (20 individuals) separately. Interviews were transcribed verbatim and analysed with thematic analysis using a dyadic framework method. Findings suggested complex experiences of adjustment among parents and adolescents which reflect two main themes – Never-Ending Abyss of Management and Diabetes Integration, with three subthemes – A Life of Food Restrictions, Evolving Familial Bonds and Technology as easing the burden of Diabetes. Dyadic analyses revealed dyadic congruence across most themes. This study adds to the adjustment literature by providing a systemic perspective rarely presented in prior paediatric research.

Over half a billion people or over 10% of the world’s population are living with diabetes globally (Sun et al., 2022). Type 1 diabetes, is a chronic lifelong auto-immune condition characterised by the bodily destruction of one’s pancreatic insulin producing beta cells, requiring an intense management regime of multiple daily injections (MDIs) or pump therapy to provide artificial insulin (Maahs et al., 2010). Type 1 diabetes-management is influenced greatly by the well-being of the individual and the support they receive (Chew et al., 2014). Without stable support and a rigorous self-management routine, the possibility of long-term diabetes complications such as peripheral vascular disease or blindness increase (Maahs et al., 2010). However, as incidence of type 1 diabetes has been increasing by up to 5% globally, with highest rates of diagnosis observed in 10- to 14-year-olds (Maahs et al., 2010), this highlights adolescence as most susceptible to adjustment issues.

Therefore, living with type 1 diabetes requires a dynamic personal and transitional adjustment style, where one’s evolving social and medical contexts are included (Kruger et al., 2023), in order to create a coherent sense of self (Oris et al., 2018) that incorporates newfound self-management processes (Gois et al., 2012). This aligns with Livneh and Antonak’s (2005) comprehensive three component definition of adjustment to chronic physical illness: (i) illness integration and internalisation of oneself as living as an individual with a chronic illness, with an updated self-concept; (ii) cognitive understanding of the illness itself (complications, daily impact and chronicity) and (iii) a renewed active pursuit of goals across personal, social and vocational domains. Furthermore, according to the Common-sense Model of self-regulation (CSM, Hagger and Orbell, 2022), perceptions about one’s illness/treatment are assumed to provide a framework for individual coping behaviours, which subsequently affects adjustment (Hagger and Orbell, 2022). The CSM model of illness perceptions (Hagger and Orbell, 2022) postulated to influence adjustment to illness, has five main cognitive domains – (i) Identity including self-labelling and symptoms, (ii) timeline, (iii) consequences/long-term impact, (iv) cause of illness and (v) perceived controllability or curability of the illness, and one noncognitive domain: emotional perceptions of the diagnosis. Correspondingly, parental illness perceptions are hypothesised to influence child perception development, with perception congruence postulated as influencing adolescent and familial adjustment to life with a chronic illness (Heyduck et al., 2015).

According to Erikson (1994) during adolescence, adjustment can be considered as the predominant developmental task of developing into relatively healthy, appropriately functioning and independent adults. However, adolescence is considered a period of particular vulnerability for patients with type 1 diabetes with relatively high rates of depression (Reynolds and Helgeson, 2011), and given the propensity for adherence deterioration and metabolic fluctuations attributed to hormone changing insulin needs, psychosocial distress and simultaneous changes in peer/parental relationships (Helgeson et al., 2018). Concurrently, research has observed that achieving a good level of psychosocial adjustment to chronic illness diagnoses is not only important for patients’ wellbeing, but also their families and greater social network (Kolokotroni et al., 2017). In line with this, parental adjustment to the illness has been reported to influence the child’s quality of life when parents report less stress (Moreira et al., 2014), and children who perceive their parents as providing positive emotional support and communication (Jaser et al., 2009), report better health-related quality of life. Therefore, assessments/interventions are proposed to focus on the family support system, rather than the patient alone (Portillo et al., 2019). Moreover, there is the subsequent balance of congruence between adolescent-parent perceptions which influence familial adjustment (Heyduck et al., 2015) highlighting the importance of parental inclusion in understanding adolescent adjustment.

Furthermore, what is known from current dyadic research includes theories of patient-parent illness responses regarding parental illness beliefs as influencing adolescent self-management (Williams-Reade et al., 2020). There is a gradual shift of responsibility to self-management throughout the course of adolescent development, yet this is often variable, depending on the nature of the task. For example, adolescents may assume responsibility for medication adherence under supervision while parents provide oversight (Markowitz et al., 2015). Furthermore, Williams et al. (2009) observed close links between poor adolescent glycaemic control, family conflict and parental psychological distress. They postulated that these effects are interactive, such that poorer family functioning is associated with poorer self-management which in turn increases family conflict and parental distress. Therefore, the more known about dyadic experiences the better we will understand and approach family relational patterns that may be influencing adolescent illness-management challenges (Helgeson et al., 2018).

There has been a recent growth in the literature regarding dyadic experiences in healthcare providing a third perspective to enrich the knowledge and perspectives of multi-person adjustment (Eisikovits and Koren, 2010). However, qualitative paediatric research does not typically include multiple perspectives (Williams-Reade et al., 2020) meaning little is known about adolescent dyadic experiences. The majority of dyadic healthcare research involves spousal/romantic relationships (Lister et al., 2013), yet romantic dyads are less significant with adolescents given their age, and parental collaboration post-diagnosis (Hanna et al., 2013). With the majority of mothers taking on primary diabetes-management till late adolescence (Schilling et al., 2006), this suggests the adolescent-parent dyad is the most pertinent developmentally, with qualitative research in college students highlighting mothers as primary caregivers and sources of emotional support beyond adolescence (Johansen et al., 2020). However, there has been a call for paternal perspectives given the propensity of research that is skewed towards maternal only views (Marshall et al., 2009).

Notably, type 1 diabetes is regarded as one of the most psychologically and behaviourally demanding chronic illnesses for both adolescents and their caregivers (Totka, 2016), highlighting the need to support these individuals where possible. However, despite the importance of adolescent-parent dyads in overall adjustment to diabetes, type 1 diabetes literature including the dyadic element has been largely quantitative in nature (Schilling et al., 2006), whilst qualitative studies have primarily focused on specific areas such as illness-management issues (Williams-Reade et al., 2020). Therefore, there is a gap in current literature regarding the dyadic experience of adjustment following a type 1 diabetes diagnosis with a need to elicit more detailed knowledge of challenges faced by early-middle adolescent-parent dyads in order to inform future interventions/clinical practice. Therefore, the purpose of this qualitative study was to understand adolescent-parent dyads experiences of adjustment to type 1 diabetes across life domains from both an individual and relational perspective.

## Methods

### Study design

A qualitative, critical realist approach was utilised to explore the experiences of the participants, as individual entities and within their respective dyads, regarding adjustment to the diagnosis of type 1 diabetes, via the use of semi-structured interviews.

### Participants

Participants (*n* = 10 dyads) included either parent, utilised as a proxy for the parental relationship, alongside their adolescent children aged between 11 and 15 years of age (Schilling et al., 2006; early-middle adolescence before self-management is dominant at 17+), at least 1 year since diagnosis. Exclusion criteria included adolescents outside of early-middle adolescence (11–15) and those who attended the department psychologist post-diagnosis to avoid any influence of therapy on adjustment. Sample size was determined based on the information power of the proposed study determined by the narrow focus on early-middle adolescence, analysis strategy and strong quality of dialogue that emerged in the interviews (Malterud et al., 2016). Table 1 outlines demographic information. Of those approached, two dyads declined participation due to one member not wanting to partake or not having enough time. No participants withdrew once they began the interviews. There was an even adolescent gender split (*n* = 5 each), while only one father participated (mothers *n* = 9). However, it is noteworthy that it was primarily mothers who accompanied their adolescents to the outpatient department.

**Table 1.** Dyad characteristics (*N* *=* 10 dyads).

Adolescent pseudonym^a^	Adolescent gender	Adolescent age	Adolescent age at diagnosis (years)	Insulin administration^b^	Parent pseudonym	Parent gender	Parent age	Dyad nationality^b^
Aoife	Female	12	10	Pump	Ava	Female	40	Irish
Ben	Male	12	10	Pens	Barbara	Female	40	Eastern European
Conor	Male	14	8	Pump	Claire	Female	47	Irish
David	Male	12	10	Pump	Dolores	Female	44	Irish
Evan	Male	13	11	Pump	Elaine	Female	50	Irish
Fiona	Female	15	2.5	Pump	Frances	Female	47	Irish
Grace	Female	15	11	Pump	Georgia	Female	49	Irish
Helena	Female	12	2	Pump	Hazel	Female	50	Irish
Issac	Male	15	9	Pens	Ian	Male	43	South American
Jessica	Female	12	1.5	Pump	Jennifer	Female	42	Eastern European

a All participants were assigned a pseudonym to preserve the confidentiality of their interviews.

b To preserve anonymity and confidentiality, Dyad’s counties of origin are collapsed to Ireland or elsewhere, and insulin administration was collapsed to pump or pens.

### Procedure

Ethical approval was granted by the Children’s Health Ireland Research Ethics Committee (REC-063-22). Recruitment occurred via information sheets sent in the post to upcoming potential participants via an independent data manager from the hospital. During three weekly adolescent outpatient clinics in May 2022, dyads were provided with separate information sheets as parents provided their own consent while adolescents under 16 years, provided written assent alongside their parent’s written consent. Dyads that provided written consent, then participated in interviews either face-to-face or online via Microsoft Teams. Semi-structured, in-depth interviews were conducted in order to explore the subjective experience of adjusting to life with type 1 diabetes, and the dynamics of the dyadic relationship in this. A pilot interview with a parent was conducted prior to data collection but not included in the analysis. Interviews were audio recorded and transcribed by the lead author. Transcripts were assigned pseudonyms to protect confidentiality. Interviews averaged 20 minutes per adolescent and 35 minutes per parent similarly observed in other parent-adolescent studies (Blackwell et al., 2016). Interviews were conducted separately in order to capture the individual as a standalone entity without forgoing the dyad.

### Measures

General opening questions concerning illness representations for type 1 diabetes were included, based on those used in a previous CSM-based healthcare study (Paterson et al., 1999). Then the Psychosocial Adjustment to Illness Scale (PAIS; Derogatis, 1986) which is a multiple domain semi-structured interview, designed to assess psychosocial adjustment to a chronic illness diagnosis in patient/caregiver was utilised. The PAIS is an internationally utilised measure that has been validated across multiple chronic illness groups and countries (Kolokotroni et al., 2017; Portillo et al., 2019). The PAIS covers seven domains including questions regarding any attitude or behaviour changes across an individual’s health care orientation, vocational environment, domestic environment, sexual relationships, extended family relationships, social environment and psychological distress (Derogatis, 1986). While initially created for spousal dyads, the PAIS has been utilised with parent-child dyads (Wakefield et al., 2015) so the sexual relationship domain was removed for this study. Studies that have observed the PAIS across patient-familial caregiver dyads have reported psychological distress experienced by family post diagnosis can impact adjustment outcomes in patients (Moser and Dracup, 2004).

### Data analysis

Interviews were transcribed verbatim, and analysed using thematic analysis (Braun and Clarke, 2014) incorporating the adapted framework method (Collaço et al., 2021). This framework method was chosen to see beyond individual experiences and into the perception of the experience as a dyad (Collaço et al., 2021) while enabling fluid movement through the data until a coherent narrative was pieced together (Gale et al., 2013). Both Microsoft Word and Excel software were utilised for data management. Following familiarisation, codes were generated before being charted into a table of themes and dyadic analysis conducted. A working analytic framework was then developed and applied before data was interpreted (Collaço et al., 2021). This method provided a template within a themed matrix so that individual’s views weren’t lost within the dyadic analysis while allowing greater transparency of the analysis process in terms of identifying congruence between the dyadic pairs (Gale et al., 2013).

The extent of congruence for each theme was discussed between authors (AEH, SH and EA) and revisions made as appropriate. It is noteworthy that the prevalence of certain topics doesn’t equate to a theme (Braun and Clarke 2014), rather prevalence combined with strength of conviction with which participants spoke about reoccurring aspects of their adjustment, resulted in the identification of the most significant themes. The data underlying this article cannot be shared publicly in order to protect privacy of participants. Explicit consent was not sought for the publication of transcripts. A table of initial interview guided themes and dyadic summaries is available in the Supplemental Data (see Table 1).

### Reflexivity

The first author was an independent researcher external to the medical team who received interview training during her Doctorate of Clinical Psychology. The first author (AEH) is living with type 1 diabetes herself which was made apparent to all potential participants during recruitment. The interviewer’s own experience with type 1 diabetes was considered important in both the recruitment process and establishing rapport. However, the data collection and analysis phases were conducted with an emphasis on maintaining openness to participant experiences given the closeness to the research topic. Bracketing (Tufford and Newman, 2012) was therefore employed through the use of memos during data collection and analysis phases, alongside reflective journaling in order to have awareness of biases/judgements. The analysis was reviewed by the co-authors to ensure transparency and primacy was given to the participants experiences of adjustment.

## Results

**Figure 1.**
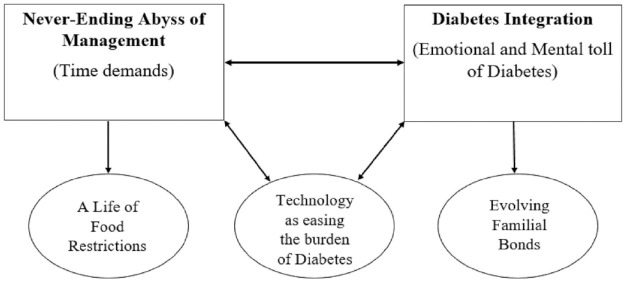
Visual depiction of the dyadic representation of the themes, subthemes and relationships between (sub)themes. The major themes are titled (1) Never-Ending Abyss of Management and (2) Diabetes Integration. The three subthemes are labelled as ‘A life of food restrictions’, ‘Technology as easing the burden of Diabetes’ and ‘Evolving Familial Bonds’.

There were two interrelated superordinate themes with three subthemes identified. The connected relationships between the (sub)themes are visually depicted in Figure 1.

### Theme 1: Never-ending abyss of management

The non-negotiable need for constant vigilant management was noted consistently across individual and dyadic perspectives ‘He has a lifelong chronic illness that has to be constantly managed and maintained’ (Dyad 3-Claire). There was dyadic congruence with regards to the resource heavy nature of diabetes management and the ongoing need for monitoring across contexts (work, school, home, etc.) and states (asleep/awake) without negotiation. Parents repeatedly discussed the rigorous night-time management from a time and sleep-deprivation perspective as well as regarding anxiety derived from lack of information while their child slept ‘The fear there . . .Would never let you rest so’ (Dyad 1-Ava). However, some adolescents diagnosed during infancy noted diabetes-management was so integrated in daily life they struggled to explain its demands ‘It’s like I don’t know (laugh). Like I do know, but like I don’t know to explain it’ (Dyad 6-Fiona).

Parents noted the amount of responsibility on them as primary managers of the illness reporting on numerous facets of life administration required: ‘It’s just a lot more organisation, a lot more thought. . .ehm planning, to ehm having his prescriptions, his appointments, having all his needs met, as in having all he needs to deal with his diabetes. . .’ (Dyad 3-Claire). Moreover, across dyads the potentially fatal or long-term complications of diabetes if poorly managed were heavily reported highlighting the relentless awareness of daily management affecting future outcomes. Adolescents commented on diabetes’ significant responsibility in terms of potential long-term complications but also regarding the physical feelings associated with blood glucose (BG) fluctuations:

I know how hard like and dangerous t-type one is, and I feel like if I do. . . if I leave my blood sugars too low, if I leave my blood sugars too high, I always have to know. . . I need to take care of it because if I don’t then. . .(pause). . .I don’t feel very nice or good’. (Dyad 10-Jessica)

#### Subtheme 1: A life of food restrictions

Throughout all interviews there was a thread regarding the management demands with regards to the perceived limitations associated with living with diabetes with particular focus on food behaviour change ‘Definitely everything, including everything to do with food, has been a lot more restricted nowadays’ (Dyad 1-Aoife). While most dyads considered their families health-conscious prior to diagnosis, food becoming more laboursome was commonplace ‘There’s no carefreeness anymore’ (Dyad 3-Claire). Dyadic agreement regarding diabetes being reframed as adding a positive increase in food-related health consciousness within a family unit was noted. However, adolescents particularly commented on food restrictions, with congruence across one dyad regarding parental food management yet the adolescent noted her subsequent regression of food autonomy: ‘But now that everything has to be measured, my parents have sort of taken over the role for every type of food now’ (Dyad 1-Aoife).

However, generally parents tended to focus their thoughts regarding food around the transition from parental to self-management and how they hoped with growing autonomy, their child’s food choices wouldn’t affect BG control ‘That’s fine as long as he just manages to bolus appropriately’ (Dyad 4-Dolores). This fear was particularly noted in one dyadic dissimilarity. While the adolescent perceived himself to be accepting of food changes since diagnosis, the father reported diabetes-related conflict over his son not taking ownership over his diabetes through sneaking low treatment food when home alone:

I came home and Issac found sweet and he eat everything but was hiding it, you know?. . . And we look after the something around. And when we look I get angry with him, you know, because it look like he doesn’t care what happens. (Dyad 9-Ian)

#### Subtheme 2: Technology as easing the burden of diabetes

There was a consistent trend of dyadic congruence of opinions regarding the benefits of technology in diabetes-management and freeing up mental space outside of diabetes. Some adolescents reported the ease of less injections, and technology as lessening the practical burden of carrying medical equipment ‘I really like the pump, like with the pumps that’s one less thing you have to carry. Without the pump beforehand I’d have to remember to bring the pen, wipes and needles’ (Dyad 5-Evan). There was incongruence in one dyad with the mother noting an information overload due to level of pump monitoring required ‘The fingerprick with your injections, you forget about the sugars. . .Whereas with this it’s much more involved’ (Dyad 4-Dolores), whereas the adolescent reported that while technology is still very laboursome they noted significant increases in health-related quality of life highlighting the pump lastly as having the greatest positive impact on his life:

There’s been like, three significant jumps in health and quality of life. . .When I first got the insulin pen because before that I just been diagnosed you know? Major quality of life improvement. When I started getting Fiasp instead of Novorapid . . .Novorapid takes 10 to 15 minutes to start acting. . .Fiasp, five or immediately you know? Really useful. . .And then, when I got the pump. (Dyad 4-David)

One dyad also reported congruent views regarding respecting the adolescent’s choice regarding new technology. The mother noted a recurrent issue with staff addressing herself concerning changing technology despite the adolescent’s clear preference for discrete technology:

The Ipso pump I have now is a lot smaller than the Animas one. . .Which makes me feel quite better and it’s like in this, like on holidays when you’re wearing like your swimsuit and stuff. . .’. (Dyad 8-Helena)

### Theme 2: Diabetes integration

Alongside the demands of diabetes management, the other superordinate theme related to the challenge versus perceived invariable accommodation of diabetes into one’s life, and for some, their sense of self. Across dyads, despite some parents struggling with acceptance, diabetes was regarded as a non-negotiable aspect of daily life that simply had to become part of a family’s routine. While one dyad showed congruence in not letting diabetes affect life goals, overall, from a dyadic perspective, there was a common emotional pattern of diverging views reported concerning the daily impact of diabetes across life in general. This entailed adolescents having accommodated diabetes into their lives and not reporting significant psychological distress, while parents noted ongoing emotional reactions even when more self-management was engaged in and theoretically less parental responsibility was required:

You have to let them go, it’s really hard, it’s one of the hardest parts about it . . .is letting go of your control over the situation and handing it over to them. . .But he has taken over a lot of control and I’ve had to let him. . .so it is . . .it’s a little easier on me now/. (Dyad 3-Claire)

Most adolescents reported diabetes as a normal aspect of life, while most parents noted diabetes had significantly impacted either their social lives ‘I wasn’t comfortable if the two of us went out. . . because I always wanted somebody to be here – Either me or John (husband)’ (Dyad 8-Hazel), or careers ‘Just leave all our jobs, our dreams, . . . focused on just her health’ (Dyad 10-Jennifer). Across one dyad while the adolescent noted not letting diabetes impact her interests, the mother reported diabetes allowed her to put her own life into perspective:

I had no time for reading books or doing any hobbies (laugh) at that time. So actually, diabetes is probably done me a favour. . .Because I've pulled back on all of that career and I’ve had more time now to do the gym and I’m playing more golf than I used to and you know, all of that had been kind of put on hold. (Dyad 7-Georgia)

Correspondingly, the level of diabetes integration into some adolescent’s sense of self was transparent given the degree of open reporting of diabetes being their ‘new normal’. Relatedly, dyadic similarities were reported in how much dyads believed a cure was probable within their lifetime or didn’t get their hopes raised:

I expect it’s going to be with me for the rest of my life. I don’t think that there’s going to be a cure for it coming out anytime soon. It may be in 50 years’ time. They'll be a magical new cure by then. But then I’m not really sure if I will want it then. I just can’t really remember what it’s like to not have it. I imagine that I won’t want to get rid of it anymore because it’ll just become something that was so regular for me, I’m not sure if I’d be able to. . . What? Just get rid of it and shift back to a normal life like I had before. (Dyad 1-Aoife)

While there was dyadic congruence with regards to the expressed hope for an eventual cure or amount of information seeking engaged in, there was divergence with regards to information content sought. Parents tended to hope/worry for their children’s future with concerns ranging from potential complications to future diabetes-related health difficulties ‘I suppose the next thing I’m thinking about for Grace is you know pregnancy that sort of thing. . .You know, it’s hard enough’ (Dyad 7-Georgia). Contrastingly, adolescents focused on present-day information seeking as vital to daily management and overall well-being:

Well, I’d like to know a. . .. lot about my treatment to a certain extent, I want to know how much you can do for me and how much you can change for the way I live. But I suppose I don’t want to really understand the way it works. I just want it to be able to help me. (Dyad 1-Aoife)

However, three dyads displayed a distinct lack of adolescent awareness about potential complications and diabetes progression, with parents holding the majority of management and trying to shield any potentially fatal complications from their children:

I mean we try and I’d say hide the potential seriousness from him like you know, I don’t know how much he would be aware of that. But you know, you still hear of people going into comas and not. . .So, I mean, we’d be aware of the seriousness, but, you know, we try and not lay too much on him. . .. (Dyad 5-Elaine)

#### Subtheme 3: Evolving familial bonds

Across dyads there was general congruence regarding each respective wider family’s inability to offer support and their lacking true understanding, with adolescents noting a need to educate family members if they did ever need support, and parents reporting on family’s belittling the impact of diabetes:

But in the beginning I did find that frustrating that people would be like, ‘Oh alright, that’s bad’ and you know and then they just get on with it. . .And they don’t realize what a life changing thing it is, and . . .What a big impact it is on the family, you know? (Dyad 4-Dolores)

While adolescents reported a close maternal bond, consistently across individual and dyadic accounts, adolescent-mother relationships were described as more serious than the ‘fun’ paternal relationships. While one dyad similarly described the mother as over-protective since diagnosis ‘No, I’m still her 5-year-old’ (Dyad 9-Issac), other adolescents noted maternal admiration for the support received ‘I love my mam. I think she is the best person in the whole wide world’ (Dyad 3-Conor). In all dyads, mothers were reported as the primary caregivers, with some noting the emotional burden of parenting an ‘ill’ child, as they felt they held the majority of the diabetes responsibility:

Yeah it mostly falls on me, but I have to say I’m not alone in it. Michael (husband) is a great help, he is also engaged, but I think he also leans on me? (laughs). . .I’m the expert now (laugh). And the same with Ben, I do feel the base, I’m definitely the go-to person when it comes to diabetes at the moment but I do work with Ben as well to kind of introduce him into you know realising signs of a low, what he has to do. . .And he does, he knows these things but he still wants to rely on me. So, yeah definitely feel that I am the go-to person and I have this responsibility at the moment. I’d do more if I have to, to be honest. . .As a mam you don’t feel it as a you know, a burden, you’d do even more, you’d do anything you need to do basically. (Dyad 2-Barbara)

## Discussion

To our knowledge, this is the first study to assess in-depth dyadic experiences of adjustment for both an adolescent living with type 1 diabetes and their parent. The findings revealed type 1 diabetes affects numerous aspects of life for the adolescent-parent dyad including relational bonds particularly with mothers, food becoming more laboursome and personal growth through integration of diabetes into daily life. Additionally, there were (sub)themes directly related to living with diabetes including technology as easing the illness burden, and the management demands. While the dyads may have experienced and managed the impacts of diagnosis differently, these were overarching aspects of life for adolescents and parents that required adjustment post-diagnosis.

While Erikson’s (1994) theory of psychosocial development defined adolescence as a period of identity formation and autonomy development, as highlighted in previous research parental-collaboration is encouraged with diabetes (Hanna et al., 2013) resulting in possible developmental delays. However, the findings of this study suggest adolescents reach a point of diabetes acclimatisation within their sense of self (Livneh and Antonak, 2005), as evidenced in the Diabetes Integration theme whereby adolescents noted diabetes as a normal aspect of life, going as far as to suggest not taking a cure should it become available one day. However, parents who have already experienced this developmental period of identity formation, appear to require a bigger adjustment to their everyday lives including career, family and social engagements in order to facilitate the inclusion of diabetes. This juxtaposes the adolescent’s active pursuit of goals across life domains despite their diagnosis, aligning with Livneh and Antonak’s (2005) physical chronic illness adjustment definition. This postulates that early-middle adolescents are at an appropriate developmental period in order to assimilate diabetes within their sense of self (Oris et al., 2018). Parents on the other hand appear to experience more difficulty and emotional distress when trying to assimilate being a parent of a chronically ill child into their identity (Kruger et al., 2023).

At the dyadic level, some themes evidenced more divergent views. The theme, Diabetes Integration, illustrated that adolescents tended to report a mental toll with more acceptance of the illness, than parents who reported difficulties in balancing life and diabetes. In line with dyadic findings from other illness groups (Heyduck et al., 2015), this study observed the parental emotional burden. Relatedly, there is a growing body of research highlighting the mental demand of diabetes including research from Stanford (Digitale et al., 2014) that observed individuals with type 1 diabetes make on average 180 health-related decisions daily. This corresponds with findings from the present study suggesting that across dyads the level of mental burden also relates to parental-management particularly during adolescence when diabetes care is monitored within the home environment. It is noteworthy that the only father who participated in this study identified himself as the ‘secondary caregiver’ to his wife, while being the only participant to note any level of diabetes-related conflict within the home. While one report of diabetes-conflict must be interpreted with caution, it also queries the need for future research to investigate familial cohesion or conflict across the familial triad to assess whether knowledge or diabetes understanding impacts family conflict in relation to self-management of type 1 diabetes.

Given that this study focused on early-middle adolescence and the likelihood of self-management being dominant for older adolescents (17+; Schilling et al., 2006) it is important to note this developmental period overlaps with the transition from parental/shared-management to self-management. Across dyads, descriptions of collaborative management in-cluded degrees of medication adherence, meal planning, (self) monitoring (Hanna et al., 2013), acceptance and emotion-regulation strategies (Heyduck et al., 2015). The emotional toll for parents of passing management responsibility was noted whereas adolescents often craved independence while appreciating tangible support. There was a striking dyadic divergence where adolescents reported technology as the best standard of care with the least amount of mental toll, while some parents reported significant technical faults that required more oversight (Schilling et al., 2006) than MDI regimes. This further highlights the ongoing mental toll perceived by the parent population (Kimbell et al., 2021) despite the growing autonomy of their adolescents.

Within the initial stages of the framework method, analysis is guided by the interview schedule including questions capturing the facets of the CSM (Hagger and Orbell, 2022). The references to information seeking and mental toll of diabetes within the Diabetes Integration theme reflect the dimensions of the CSM of self-regulation which are assumed to provide a framework for individual coping and subsequent adjustment (Hagger and Orbell, 2022). Therefore, the findings of this study align with the CSM as the dyadically reported informed illness perceptions, via the coping method of proactive information seeking, allowed for diabetes integration or adjustment, as a ‘new normal’ aspect of daily life. Similar to asthmatic adolescent-caregiver dyads (Heyduck et al., 2015), the variety of long- and short-term consequences of diabetes reported across dyads demonstrated not only the numerous potential impacts on medical, psychological and social domains of the adolescents’ daily lives, but also emphasised the inter-individual variability within adjustment. Furthermore, the trend with mother-adolescent relationships being described by adolescents as very close but more serious than with their fathers aligns with previous research that suggests mothers take the primary caregiver role (Heyduck et al., 2015) assuming diabetes responsibility till self-management occurs (Schilling et al., 2006). Similarly, it is noteworthy that studies that have compared parental outcomes regarding illness burden and impact have observed mothers report significantly greater burden related to medical treatment and more emotional distress than fathers (Haugstvedt et al., 2011). However, fathers are reported to be more willing in diabetes-management when technology is involved (Kimbell et al., 2021). Furthermore, the dyadic emotional closeness in this study aligns with the ongoing emotional support university students with type 1 diabetes report getting from their mothers (Johansen et al., 2020).

Overall, the findings of this study support the relevance for adolescents of Livneh and Antonak’s (2005) definition of chronic physical illness adjustment across three components: (i) illness integration, (ii) cognitive understanding of the illness and (iii) a renewed pursuit of goals across life domains. Adolescents clearly reported an internal representation of themselves as individuals living with diabetes, with parents potentially struggling with this as hypothesised above due to prior identity formation (Erikson, 1994). Adolescents engaged in active pursuit of goals across life domains despite their illness, while parents reported a gradual return or newfound goals because of diagnosis. In contrast, while all parents reported good cognitive understanding of the illness itself, there were three adolescents (see Diabetes Integration theme), that were shielded from the chronicity and potential complications of diabetes. Therefore, while all components of the Livneh and Antonak (2005) definition were mentioned across dyads, it appears that this definition is more pertinent to the individual living with the illness themselves rather than the caregiver. This queries the need for such a definition for the parental cohort who are adjusting alongside their children (Wright & Kirby, 1999).

Limitations to this study included variability amongst participants in terms of diagnosis duration. On average the time since diagnosis was two years, however there were three dyads where over ten years had elapsed since diagnosis, suggesting a possible recall bias. However, adjustment to adolescence and changing management dynamics were similarly reported. The duration since diagnosis in these dyads may also have influenced the development of the dyadic relationships in terms of parent protectiveness. It is noteworthy that only one father participated in this study, however, given it was primarily mothers who accompanied their adolescents to the outpatient department, consistent with past research (Schilling et al., 2006), our dyadic sample is reflective of the setting.

As highlighted by Portillo et al. (2019) and reinforced with these findings, psychological adjustment to an illness is a complex process whereby targeted interventions are required that reflect the interactive components and the multi-faceted care where patients, parents and healthcare professionals interact. Therefore, future research could aim to consider the dyadic experience of adjustment to a new type 1 diabetes diagnosis, through exploring dyadic congruence on a systemic level. This could be utilised to develop therapeutic interventions aiming to enhance familial diabetes-related engagement, behaviours and adherence outcomes for long-term quality of life (Hanna et al., 2013). By routinely monitoring parents’ representations regarding type 1 diabetes (Hagger and Orbell, 2022) and subsequently normalising differences between adolescent-parent representations this may be worthwhile in the facilitation of familial psychosocial education and creating a cohesive approach to diabetes-management/adjustment. It is also recommended to explore healthcare practitioners’ understandings of adolescent-parent adjustment versus management and their role in encouraging collaboration (Hanna et al., 2013). Furthermore, supportive guidance programmes that facilitate adolescent-parent adjustment following a type 1 diabetes diagnosis should be developed to foster collaborative relationship strategies/behaviours particularly for management and emotional wellbeing. Such guidance is needed to raise awareness of existing resources/tools amongst healthcare professionals that could support discussing dyadic adjustment and cooperative management.

Overall, adolescent-parent dyads experience changes in their approach to food, mother-child relationships, management strategies, outlook on technology and overall integration of diabetes into their lives. Adolescents and parents adjusted separately as individuals while also impacting the dyadic adjustment as parents took pride in their children’s resilience, and adolescents appreciated the unconditional care/support provided to them. Regardless of duration since diagnosis, adjustment from the dyadic perspective was dependent on integration of diabetes into their lives and managing the ongoing mental toll together. It is important that adolescent-parent dyads have access to services that can address their adjustment needs and help support them with the mental burden of type 1 diabetes.

## Supplemental Material

sj-docx-1-hpq-10.1177_13591053231216700 – Supplemental material for ‘It’s life threatening, it’s not life limiting but it’s life threatening’ – Dyadic framework analysis of adolescent and parent adjustment to a type 1 diabetes diagnosis Supplemental material, sj-docx-1-hpq-10.1177_13591053231216700 for ‘It’s life threatening, it’s not life limiting but it’s life threatening’ – Dyadic framework analysis of adolescent and parent adjustment to a type 1 diabetes diagnosis by Andrea Habenicht, Elayne Ahern, Declan Cody, Vincent McDarby and Sharon Houghton in Journal of Health Psychology

## References

[bibr1-13591053231216700] Blackwell L Gardiner E Schoenebeck S (2016) Managing expectations: Technology tensions among parents and teens. In Proceedings of the 19th ACM conference on computer-supported cooperative work and social computing, San Francisco, pp. 1390–1401.

[bibr2-13591053231216700] Braun V Clarke V (2014) What can ‘thematic analysis’ offer health and wellbeing researchers? International Journal of Qualitative Studies on Health and Well-Being 9(1): 26152. 25326092 10.3402/qhw.v9.26152 PMC4201665

[bibr3-13591053231216700] Chew BH Shariff-Ghazali S Fernandez A (2014) Psychological aspects of diabetes care: Effecting behavioral change in patients. World Journal of Diabetes 5(6): 796. 25512782 10.4239/wjd.v5.i6.796 PMC4265866

[bibr4-13591053231216700] Collaço N Wagland R Alexis O , et al (2021) Using the framework method for the analysis of qualitative dyadic data in health research. Qualitative Health Research 31(8): 1555–1564. 33980102 10.1177/10497323211011599 PMC8278550

[bibr5-13591053231216700] Derogatis LR (1986) The psychosocial adjustment to illness scale (PAIS). Journal of Psychosomatic Research 30(1): 77–91. 3701670 10.1016/0022-3999(86)90069-3

[bibr6-13591053231216700] Digitale E Leggett H Vaughan C (2014) New research shows how to keep diabetics safer during sleep. Scope. Available at: https://scopeblog.stanford.edu/2014/05/08/new-research-keeps-diabetics-safer-during-sleep/ (accessed 16 May 2022).

[bibr7-13591053231216700] Eisikovits Z Koren C (2010) Approaches to and outcomes of dyadic interview analysis. Qualitative Health Research 20(12): 1642–1655. 20663940 10.1177/1049732310376520

[bibr8-13591053231216700] Erikson EH (1994) Identity Youth and Crisis (No. 7). WW Norton & Company, New York.

[bibr9-13591053231216700] Gale NK Heath G Cameron E , et al (2013) Using the framework method for the analysis of qualitative data in multi-disciplinary health research. BMC Medical Research Methodology 13(1): 1–8. 24047204 10.1186/1471-2288-13-117 PMC3848812

[bibr10-13591053231216700] Gois CJ Ferro AC Santos AL , et al (2012) Psychological adjustment to diabetes mellitus: Highlighting self-integration and self-regulation. Acta Diabetologica 49(1): 33–40. 20473694 10.1007/s00592-010-0191-7

[bibr11-13591053231216700] Hagger MS Orbell S (2022) The common sense model of illness self-regulation: A conceptual review and proposed extended model. Health Psychology Review 16(3): 347–377. 33461402 10.1080/17437199.2021.1878050

[bibr12-13591053231216700] Hanna KM Dashiff CJ Stump TE , et al (2013) Parent–adolescent dyads: Association of parental autonomy support and parent–adolescent shared diabetes care responsibility. Child: Care, Health and Development 39(5): 695–702. 22380684 10.1111/j.1365-2214.2012.01373.x PMC3371322

[bibr13-13591053231216700] Haugstvedt A Wentzel-Larsen T Rokne B , et al (2011) Perceived family burden and emotional distress: Similarities and differences between mothers and fathers of children with type 1 diabetes in a population-based study. Pediatric Diabetes 12(2): 107–114. 20522171 10.1111/j.1399-5448.2010.00661.x

[bibr14-13591053231216700] Helgeson VS Vaughn AK Seltman H , et al (2018) Featured article: trajectories of glycemic control over adolescence and emerging adulthood: An 11-year longitudinal study of youth with type 1 diabetes. Journal of Pediatric Psychology 43(1): 8–18. 28510719 10.1093/jpepsy/jsx083 PMC5884394

[bibr15-13591053231216700] Heyduck K Bengel J Farin-Glattacker E , et al (2015) Adolescent and parental perceptions about asthma and asthma management: A dyadic qualitative analysis. Child: Care, Health and Development 41(6): 1227–1237. 26283038 10.1111/cch.12277

[bibr16-13591053231216700] Jaser SS Whittemore R Ambrosino JM , et al (2009) Coping and psychosocial adjustment in mothers of young children with type 1 diabetes. Children's Health Care 38(2): 91–106. 10.1080/02739610902813229 PMC2675938 19412355

[bibr17-13591053231216700] Johansen CB Rothmann MJ Andersen A , et al (2020) The role of parental support for emerging adults with type 1 diabetes: A scoping review. Pediatric Diabetes 21(6): 995–1030. 32301182 10.1111/pedi.13022

[bibr18-13591053231216700] Kimbell B Lawton J Boughton C , et al (2021) Parents’ experiences of caring for a young child with type 1 diabetes: A systematic review and synthesis of qualitative evidence. BMC Pediatrics 21(1): 1–13. 33814007 10.1186/s12887-021-02569-4 PMC8019496

[bibr19-13591053231216700] Kolokotroni P Anagnostopoulos F Missitzis I (2017) Psychosocial Adjustment to Illness Scale: Factor structure, reliability, and validity assessment in a sample of Greek breast cancer patients. Women & Health 57(6): 705–722. 27158891 10.1080/03630242.2016.1186780

[bibr20-13591053231216700] Kruger S Deacon E van Rensburg E Segal DG (2023) Young adult women’s meaning-making of living with type 1 diabetes: Towards growth and optimism. Psychology & Health 38(5): 573–590. 34510968 10.1080/08870446.2021.1977303

[bibr21-13591053231216700] Lister Z Fox C Wilson CM (2013) Couples and diabetes: A 30-year narrative review of dyadic relational research. Contemporary Family Therapy 35(4): 613–638.

[bibr22-13591053231216700] Livneh H Antonak RF (2005) Psychosocial adaptation to chronic illness and disability: A primer for counselors. Journal of Counseling & Development 83(1): 12–20.

[bibr23-13591053231216700] Maahs DM West NA Lawrence JM , et al (2010) Epidemiology of type 1 diabetes. Endocrinology and Metabolism Clinics 39(3): 481–497. 20723815 10.1016/j.ecl.2010.05.011 PMC2925303

[bibr24-13591053231216700] Malterud K Siersma VD Guassora AD (2016) Sample size in qualitative interview studies: guided by information power. Qualitative Health Research 26(13): 1753–1760. 26613970 10.1177/1049732315617444

[bibr25-13591053231216700] Markowitz J Garvey K Laffel L (2015) Developmental changes in the roles of patients and families in type 1 diabetes management. Current Diabetes Reviews 11(4): 231–238. 25901503 10.2174/1573399811666150421114146 PMC4826732

[bibr26-13591053231216700] Marshall M Carter B Rose K Brotherton A (2009) Living with type 1 diabetes: Perceptions of children and their parents. Journal of Clinical Nursing 18(12): 1703–1710. 19646116 10.1111/j.1365-2702.2008.02737.x

[bibr27-13591053231216700] Moreira H Frontini R Bullinger M , et al (2014) Family cohesion and health-related quality of life of children with type 1 diabetes: The mediating role of parental adjustment. Journal of Child and Family Studies 23: 347–359.

[bibr28-13591053231216700] Moser DK Dracup K (2004) Role of spousal anxiety and depression in patients’ psychosocial recovery after a cardiac event. Psychosomatic Medicine 66(4): 527–532. 15272098 10.1097/01.psy.0000130493.80576.0c

[bibr29-13591053231216700] Oris L Luyckx K Rassart J , et al (2018) Illness identity in adults with a chronic illness. Journal of Clinical Psychology in Medical Settings 25(4): 429–440. 29468569 10.1007/s10880-018-9552-0

[bibr30-13591053231216700] Paterson J Moss-Morris R Butler SJ (1999) The effect of illness experience and demographic factors on children’s illness representations. Psychology and Health 14(1): 117–129.

[bibr31-13591053231216700] Portillo MC Ambrosio L Lanas Martín R , et al (2019) A pilot study on the Spanish version of the Psychosocial Adjustment to Illness Scale (PAIS-SR) with carers of people with Parkinson’s disease. Nursing Open 6(3): 1262–1268. 31367453 10.1002/nop2.329 PMC6650674

[bibr32-13591053231216700] Reynolds KA Helgeson VS (2011) Children with diabetes compared to peers: Depressed? Distressed? A meta-analytic review. Annals of Behavioral Medicine 42(1): 29–41. 21445720 10.1007/s12160-011-9262-4 PMC3140576

[bibr33-13591053231216700] Schilling LS Knafl KA Grey M (2006) Changing patterns of self-management in youth with type I diabetes. Journal of Pediatric Nursing 21(6): 412–424. 17101399 10.1016/j.pedn.2006.01.034

[bibr34-13591053231216700] Sun H Saeedi P Karuranga S , et al (2022) IDF Diabetes Atlas: Global, regional and country-level diabetes prevalence estimates for 2021 and projections for 2045. Diabetes Research and Clinical Practice 183: 109119. 34879977 10.1016/j.diabres.2021.109119 PMC11057359

[bibr35-13591053231216700] Totka JP (2016) Type 1 diabetes: Factors that affect youth/parent dyads’ health related quality of life and youth metabolic control. Doctoral Dissertation, The University of Wisconsin-Milwaukee.

[bibr36-13591053231216700] Tufford L Newman P (2012) Bracketing in qualitative research. Qualitative Social Work 11(1): 80–96.

[bibr37-13591053231216700] Wakefield CE Sansom-Daly UM McGill BC , et al (2015) Online parent-targeted cognitive-behavioural therapy intervention to improve quality of life in families of young cancer survivors: Study protocol for a randomised controlled trial. Trials 16(1): 1–12. 25872773 10.1186/s13063-015-0681-6 PMC4395969

[bibr38-13591053231216700] Williams LB Laffel LMB Hood KK (2009) Diabetes-specific family conflict and psychological distress in paediatric type 1 diabetes. Diabetic Medicine 26(9): 908–914. 19719712 10.1111/j.1464-5491.2009.02794.x PMC4930362

[bibr39-13591053231216700] Williams-Reade JM Tapanes D Distelberg BJ , et al (2020) Pediatric chronic illness management: A qualitative dyadic analysis of adolescent patient and parent illness narratives. Journal of Marital and Family Therapy 46(1): 135–148. 30725488 10.1111/jmft.12377

[bibr40-13591053231216700] Wright SJ Kirby A (1999) Deconstructing conceptualizations of ‘adjustment’ to chronic illness: A proposed integrative framework. Journal of Health Psychology 4(2): 259–272. 22021484 10.1177/135910539900400219

