# Supplementary material for: ‘It’s life threatening, it’s not life limiting but it’s life threatening’ – Dyadic framework analysis of adolescent and parent adjustment to a type 1 diabetes diagnosis
Source: J Health Psychol. 2023 Dec 30;29(8):905–17. doi: 10.1177/13591053231216700 (PMC11264551; doi:10.1177/13591053231216700)
Supplement: sj-docx-1-hpq-10.1177_13591053231216700 – Supplemental material for ‘It’s life threatening, it’s not life limiting but it’s life threatening’ – Dyadic framework analysis of adolescent and parent adjustment to a type 1 diabetes diagnosis [file sj-docx-1-hpq-10.1177_13591053231216700.docx]

**Table 1- Charted table of Themes and Dyadic Summaries.**

|  | **Theme** | | |  |
| --- | --- | --- | --- | --- |
|  | **Illness Representations of Diabetes** | | |  |
| **Dyad** | **Subthemes** | **Adolescent** | **Parent** | **Dyadic Code/Summary** |
| **1** | **Diabetes Awareness** | Good awareness of auto-immune nature of condition, different types of diabetes and seriousness of illness (lines 14-36): *It can be quite serious if it's left untreated, because then that…Well, it's most likely that you will end up dying if you just stick your head in the sand and ignore the fact that you have type one diabetes, but when it's managed, you live a relatively normal life and not under much threat of dying* | Good awareness of auto-immune condition and need for artificial insulin (lines 13-15). Lots of concern about immediate and future potential complications that general public are unaware about (lines 50-60): *That she would have a severe hypo and ehm die. That's the more immediate issue…But in the long run that her quality of life and maybe not so long run, that's a quality of life, could be detrimentally affected. Umm yeah or, you know. Yeah. So the hypo thing is is you know, if if she was unable to manage* *herself or we weren't able to to intervene …And you know people around her didn't understand that could lead to a life or death situation. But but in the long run off the all the other impacts could could affect her quite a lot as well.* | **Acceptance versus Fear**  Adolescent reported diabetes as a unlucky but accepted part of life. Mother on the other hand provided an in-depth insight into her variety of present and future concerns for living with diabetes that were not voiced by the adolescent. While the adolescent acknowledged the potentially fatal nature of the illness when untreated, they didn’t go into the detail of concern that the parent did highlighting potentially different stages of adjustment to the illness. |
|  | **Emotional Perceptions and Identity** | Better to understand the illness to try and manage it (Lines 12-13). Awareness of food management as more laboursome with diabetes. Perceiving other individuals with diabetes as trying to ignore their condition by eating low carb. Diabetes as affecting self, but awareness of cards dealt in life. (Lines 50-60): *Well, it's …(pause)…definitely changed… my life…Not necessarily for the better, but then not necessarily for the worst. It's just something that I suppose happened to me, and now I have to live with it.* | Devastation at shock diagnosis with difficult adjustment period since (lines 18-23): *we weren't worried about her or anything. So it's, it was a big shock and it's been a difficult adjustment. It's getting better but I would say the first year and a half have been very tough.* The lifelong serious nature and management of the illness at the forefront of the mother’s worries (lines 35-44). Technology as providing some release from the mental toll of diabetes but also fear at handing over management (lines 89-100): *You know, her lifetime is a very long period of time (laugh) and things could change and maybe she won't have a pump. I don't know. You know, so but but yeah the pump is great. There's a lot of thinking taken away. Not everything, but a lot of it's gone. But she should be able to do that herself. And and certainly she she isn't very aware. She's she's not. She's she's happy and she's well. Ehm but she does rely on us a huge amount. So how how shall manage it independently is the biggest worry I think.* | **Management as key**  Both adolescent and parent reported on the never-ending management required to keep diabetes maintained. However, the adolescent highlighted food as a major focus and quite a laboursome aspect to their management while the parent highlighted how while technology has eased the mental burden it provides more worries with the transition from parental to child management.  OVERLAP WITH DIABETES MANAGEMENT |
|  | **Healthcare Orientation** | | |  |
| **Dyad** | **Subthemes** | **Adolescent** | **Parent** | **Dyadic Code/Summary** |
| 1 | **Attitudes towards healthcare** | Acknowledging good health and lack of health consciousness required before diagnosis. Diabetes as the assumed conclusion for all issues now – family, school or healthcare (lines 79-83): *And it's not just necessarily me, at school It's also changed a bit. So not with the other students, with the teachers and sometimes with family members, if there's anything, if I even really feel a bit tired, people usually link it to my diabetes and ask if I'm feeling fine. Do I need something? Am I okay? When it’s usually me just feeling tired or something like that.* Discussed diabetes as common illness with good experiences in hospital yet surprise at the limited treatment options and how slow it is to receive the technology (lines 88-98). | Reported herself as health conscious prior to the diagnosis but acknowledged lack of awareness around diabetes. Understood two main types and that one was generally related to kids but huge knowledge acquisition with the diagnosis (lines 114-129). Treatment options were described as beneficial when received but noted the long wait times for training as frustrating. Discussed the healthcare team as busy but helpful meaning parents often trying to figure it out alone (lines 178-185): *They're very busy. You know. So sometimes, you know. If you're having an issue that you know you can bring them out, ask whatever. But they're only seeing a very small snapshot, and it's hard to get into the detail of trying to understand…You know, why is this happening? Why does it keep happening, or whatever, you know, so probably we, you know, we we tend to just muddle along by ourselves because they just didn't really have the time to to give it…* | **Diabetes at forefront of mind**  Both mentioned respectively how diabetes is the first thought- adolescent in reference to everyone around her, and parent for herself. Parent noted the huge information acquisition with diagnosis and the subsequent learning on the go now. |
|  | **Diabetes Management** | No real mention of personal management. | Focus on the parental control of food and mealtimes and food as becoming more laboursome with the diagnosis. Discussed the mental toll of diabetes and the never-ending information. Learning about the factors that affect adolescent’s BGs and discussed the affect on parental sleep while noting the improvements for this with technology. While technology has provided some relief in the relentless parental management needed there was still significant fear and worry reported (Lines 133-171): *Previous to the pump, it wouldn't have been particularly stable and you could easily have been having lows overnight. So ehm the fear there …Would never let you rest so.* | **Differing management demands**  Adolescent commented on management as key to good quality of life but didn’t go into details of what that entailed while the parent recounted in great detail the significant mental and time demands that diabetes creates. Technology reported as relieving some of the mental concerns, but diabetes was reported as always on the mind.  OVERLAP WITH EMOTIONAL PERCEPTIONS AND IDENTITY |
|  | **Future expectations** | Discussed not realistically thinking she will see a cure in the recent future. Also acknowledgement of potentially not wanting a cure as she already doesn’t remember life pre-diabetes and it has become a part of her but referring to life pre-diabetes as “normal” (lines 101-107): *I expect it's going to be with me for the rest of my life. I don't think that there's going to be a cure for it coming out anytime soon. I'm not sure. It may be in 50 years’ time. They'll be a magical new cure by then. But then I’m not really sure if I will want it then. I just can’t really remember what its like to not have it. It just…It's just something that's irregular and something like 50 years passing by. I imagined that I won't want to get rid of it anymore because it'll just become something that was so regular for me, I'm not sure if I'd be able to… What? Just get rid of it and shift back to a normal life like I had before.* | Reported about the evolution of diabetes care already (last 100 years) and not wanting to raise hopes for a cure as just trying to manage the current mental toll and getting to grips with diabetes (lines 220-229): *They've come a long way already. I can see that and that, you know, hope for even more to dramatic changes. Ehm… Which is great, but to be honest I don't really get into much detail on it because…You just don't know what the future holds, and I I don't want to (pause) raise my hopes I suppose too much that things could change significantly. I think just, you know, getting to grips with the day to day is kind of what where my head is at. So…And if it improves more great. But, you know, I, you know, get yeah, just kind of let's let's manage what we have and ehm and just carry on from there.* | **Emotional processing of life with Diabetes**  Both reported on a potential future cure and while the parent doesn’t want to raise her hopes about it the adolescent was unsure would she even take it when it eventually happens. The adolescent is processing diabetes as a part of herself and a new normal aspect of her life. Parent was trying to prioritise the current management and learnings and not get too hopeful for future progressions. |
|  | **Information seeking** | Information as making management easier and management being key to quality of life. Noting that there is a thing as too much information and not seeking to know about complications, but rather about treatment options and management techniques (lines 115-130): *Well, I'd like to know a…. lot about my treatment to a certain extent, I want to know that how much you can do for me and how much you can change for the way I live. But I suppose I don't want to really understand the way it works. I just want it to be able to help me...I suppose.* | Reported little information supplied regarding technology and having to get to grips with diabetes and initial treatment (pens) before pumps became an option. Noted how a pump was chosen from parental peer support group information (lines 247-250): *I didn't really know a huge amount about other types, so I I'd seen eh So I I we've joined a Facebook group for parents with ehm type one that's very active and there's a lot of different discussion and questions and stuff like that and that's that name have been mentioned a few times. So that's what I went with and it seemed to be getting good reviews* | **Treatment information as relating to Quality of Life**  Information about treatment options were highlighted as crucial for improving quality of life. Adolescent noted only researching what would help her manage her condition while parent reported peer support groups as providing first hand parenting accounts of technology rather than information seeking herself or through the clinic. |
|  | **Impact on the Vocational Environment** | | |  |
| **Dyad** | **Subthemes** | **Adolescent** | **Parent** | **Dyadic Code/Summary** |
| 1 | **Vocational Performance** | Pump alarms as disrupting to the school day as immediate treatment is non-negotiable. Noted diabetes can be othering with peers as it’s done publicly so they stare making her self-conscious (Lines 136-139): *sometimes I get looks from the students when I go over to the person who helps me with my diabetes… and I definitely get look for it when I try to treat a low- I usually treat my Lows with drinking very small cans of coke and definitely people are looking by then even when they're meant to be doing their work.* Also noted that peers curiosity is never really vocalised so it's an unsaid thing in the classroom (lines 159-161): *people aren't generally very nosy upfront. They don't ask you what is wrong with you… They just sort of look at it. They just sort of wonder what it is. But they don't ever say it out loud.* | Noted the mental toll if diabetes and how it was a constant distraction from work during the early days. Diagnosis happening during COVID-19 was nearly regarded as a blessing as no time off work was needed but increased and desirable parental monitoring and management was achievable (lines 266-268): *It does distract me, obviously quite a lot and with the yeah, monitoring, but in a way we were sort of lucky that Aoife was diagnosed during the pandemic and I was working from home.* Noted awareness of attention levels not where they should be in work but trying to balance diabetes and life. | **Integrating Diabetes into daily life**  Differing perspectives here as adolescent reported on the experiential peer aspect of diabetes othering with peers and feeling different within school. Whereas the parent reported around the ongoing mental demands of diabetes within the work day and how that has to be integrated. |
|  | **Vocational Values** | No reported affect of diabetes on importance of school or having to change any school goals (lines 155-156): *My goals are still generally just the same. Diabetes has never really interfered with what I wanted to do academically.* | No reported impact on work goals but noted directly post diagnosis job importance went way down (lines 302-304): *Say for the first while it was very much on the back burner. Yes, as things have settled, it's sort of resumed, you know where it wants to be anyway.* | **Diabetes not affecting long-term goals**  Both acknowledged that diabetes wasn’t really having a long-term effect on goals. Parent noted the importance of her job was put into perspective directly post diagnosis but this has rectified itself again. |
|  | **Impact on the Home Environment** | | |  |
| **Dyad** | **Subthemes** | **Adolescent** | **Parent** | **Dyadic Code/Summary** |
| 1 | **Impact on Relationships** | Reported good parental relationships and no change in sibling dynamic since diagnosis (lines 173-174): *My sister. She doesn't seem to really care that I have diabetes. It really hasn't interfered with our relationship at all.* Later reported some sibling jealously over low treatments (lines 180-181): *sometimes my sister gets jealous when she sees me drinking coke for a low and she'll sometimes get mad when she's refused to have one.* Reported a sense of being alone in her diabetes as family cannot truly understand the condition but noting no affect of diabetes on communication (lines 193-194): *So, no one really knows… what it's like to have diabetes. They can only just… see it. But I don't think there's a lack of communication* | Adolescent emotional support and safety gained from parental management resulting in no perceived resentment towards the illness (lines 315-319): *It's good, good relationship. She's very independent and knows her own mind, but she she relies on us and trusts us a lot in terms of her medical health and…feel safe with us, I think, because knowing that we're always have an eye on things so. Ehm I I know she she doesn't worry about it, which is good.* Noted impact of diabetes on the sibling but reported good understanding for the most part. With spousal relationship noted very purposeful tag team effort to maintain optimal parental management (lines 334-338): *with my husband, I supposed to definitely been very challenging, very stressful. Ehm at times, you know It's, it's brought out the best and the worst of us because it's teamwork and it's, you know, we work… (pause)… we have to work as a team to to to to cover it all I suppose.* Also noted increase in communication and general common topic of diabetes within the family (lines 344-347): *probably more because we always are checking in, you know? Yeah. What do you think happened here or, you know, yeah, what we do next. And so it's yeah, it's always it's always a topic of conversation.* | **Dynamics of diabetes relational impact**  Both members of dyad reported good overall relationship quality since diagnosis. Adolescent noted sibling jealously and a lack of true understanding when not living with the condition itself. Parent noted hoping that parental management is shielding the adolescent from full burden of illness. Noted challenging impact on spousal relationship with diagnosis and having to juggle life and diabetes. |
|  | **Quality of Support** | Noted the parental management of carb counting as leading to a regression in her food making independence (lines 188-189): *But now that everything has to be measured, my parents have sort of taken over the role for every type of food now.* Discussed not liking having to ask for help with diabetes and alluded to an illness identity post diagnosis of being ill (lines 198-200): *Well, sometimes I need help but…(pause)…Generally, I don't like … having to get help for… because of my illness, I just sort of don't want to be seen as the same way I was when I first got out of hospital with diabetes- as the poor, sickly child.* | Noted spousal support as a tag team effort with diabetes management and balancing life (lines 359-360): *The same work, yeah because you know, the various jobs from type one are shared and ehm along with everything else.* Noted that while there is support there for practical issues that diabetes is a family contained issue (lines 366-367): *I think there is, but not with, not with the type one stuff itself. Yeah, there isn't anyone who's I think felt comfortable with that.* | **Diabetes Illness Identity and Family Identity**  Adolescent noted a regression in her independence and feeling as though she is perceived as “sickly” suggesting a poor internal diabetes illness representation. The parent however reported on diabetes as being a responsibility of the family alone as others aren’t comfortable with the risk or management. |
|  | **Impact on Family life** | Diabetes as interfering with meal timings and trying to eat as a family. Knock-on effects of BGs on the wider family (lines 177-179): *Umm well generally my diabetes has interfered…with…The times we do things for, as such, if I'm having a high before dinner, generally everyone has to stop eh eating dinner and wait for my bloods to go back down. So, then we can all eat dinner together*. Acknowledged lack of knowledge around financial impact of diabetes and parental focus on efficacy and quality of life (lines 211-213): *I don't think… there's been too much of demand. Umm, my parents really don't look that worried whenever we're umm buying things for our diabetes, they just always seem kind of more focused on what the diabetes things are gonna do and if they're gonna work.* | No impact on home duties as diagnosis coincided with the pandemic. Reported Long Term Illness Scheme (LTI) as beneficial in not having to pay for most supplies but sourced some additional bits outside of this as well (lines 380-381): *all the supplies are available to us and the pump itself. Ehm so we haven't had to pay anything for any of that.* | **Perspectives on Family Life Impact**  Adolescent noted being conscious of making family members wait to have a meal together based on her BGs while mother reported no major impact on home life. Highlighting the diabetes consciousness for the adolescent. |
|  | **Extended Family Relationships** | | |  |
| **Dyad** | **Subthemes** | **Adolescent** | **Parent** | **Dyadic Code/Summary** |
| **1** | **Extended family communication** | Noted Pandemic had a big effect on communication more so than diagnosis. However, reported pity from cousins with family reactions (lines 220-221): *Some of my cousins Umm, they found out that, that they felt sorry for me and they just said I hope that I'm doing fine.* Noted no major impact on level of communication or socialisation since prominence at diagnosis, but did report similar to school peers lots of staring during treatment (lines 238-241): *We haven't really had much discussion about the diabetes. It's just something that's there sometimes, people will look at me if I have a low or high, or if I just even check my blood sugar, but it's just generally not… a hot topic in the family anymore. Ever since I got it.* | Wider family nervousness post-diagnosis resulting in less socialisation and communication (lines 395-398): *I probably ehm it which could be linked to being nervous about going to various, you know, like family events or whatever and yeah, and yeah, just I think others being nervous and us being nervous as well. It’s not, probably not a current issue, but they may have been in the past.* Noted the parental judgement may have influenced interest and engagement in wider family occasions (line 423): *diabetes and and uncertainty and and fear* due to perceiving others as nervous, seeing diabetes as too difficult for a babysitter and therefore excluding them for events (lines 402-409): *So it's like they're thinking it's like there's a family sort events where we might have all gone, but and then maybe have been nervous about it. There's events that she probably hasn't gotten to because I think other people are nervous of it. And…Then there's, you know, like getting out, you know, babysitters, things like that. That's definitely, definitely reduced and ehm, is still very much reduced. So yes, it's just too big an ask for most people.* Also noted initial engagement post-diagnosis as influenced by feeling guilty at parties and not knowing how to manage this for their child (lines 427-428): *in the early days it just seemed kind of cruel to, you know, host a party with lots of sweets and stuff around when we really didn't know how to manage that for her.* | **Diabetes related othering**  Adolescent reported on reactions from family members and being self-conscious with treating diabetes publicly. The parent however maybe inadvertently also othered the child reporting less immediate family engagement in wider family occasions due to expecting fear or nervousness. |
|  | **Extended family quality of support** | Reported desire for independence and not wanting diabetes to impact on this (lines 229-231*): I don't like to think that I really need to be supported, but, because of my diabetes, I like to think instead that I can sort of do it on my own and I don't need other people to try and help me get through, mental or physically.* | Reported access to practical support if required (lines 415-416): *No, that's there if we need it … but we generally don't need it but it is there if we need it.* | **Family value on independence**  Adolescent noted her desire for independence and not wanting familial help. Similarly, mother reported on not needing help but knowing she could avail if required suggesting a value for keeping diabetes as contained within the family or as an independent issue. |
|  | **Social Environment** | | |  |
| **Dyad** | **Subthemes** | **Adolescent** | **Parent** | **Dyadic Code/Summary** |
| 1 | **Impact on interest levels & participation** | Noted sedentary activities aren’t affect by fluctuating BG levels (lines 250-251): *I don't ever have to stop reading or drawing because my blood sugar is too low or too high because of those things.* However, she noted requiring more planning for any physical activities (lines 262-263): *I suppose I have to be more thought put into it because of my diabetes.* Reported main impact on social activities as regular parental check-ins for carb counting and monitoring (lines 269-273): *sometimes my mom has to… Umm, she wants to, she texts me a lot when I am out, not, not with her or dad… So just so I know what I'm doing and I have to tell her everything. I've been eating at the movies or in someone else's house or restaurant or whatever.* | Noted the interest and participation are both affected by the constant need for management and parental responsibility with diabetes (lines 448-450): *I think my attention levels have reduced, I guess maybe partly be… because the there's a certain amount needs in the background and monitoring things. And I I just find my attention span is greatly reduced.* Reported sporadic engagement in own hobbies and lack of consistency in own life since diagnosis. Noted spousal activities are non-existent since diagnosis as parental concern when both away from child is too high (lines 475-477): *if it's something that I can do on my own with other people, it's not that hard to arrange. But if it's something that the two of us(husband as well) ehm need to get out for that’s much more difficult. So that's fairly…not non-existent.* | **Physical versus Mental impact of Diabetes**  Adolescent reported on the physical aspect of diabetes impacting activities with more thought and planning required whereas the parent reported about the never-ending mental toll of diabetes. Highlighted by the fact that one or other parent is with child almost constantly due to concern. |
|  | **Psychological Distress** | | |  |
| **Dyad** | **Subthemes** | **Adolescent** | **Parent** | **Dyadic Code/Summary** |
| 1 | **Mental toll of Diabetes** | Noted no need for anxiety about diabetes but some initial sadness post-diagnosis with future uncertainty (lines 284-286): *It just generally I knew that then my life was really going to change even more than I thought it was going to change, and it just generally, I didn't, I wasn't sure folks were going to like the way my life has changed and the way things were going to be from now on.* Reported peers regarding her illness as an inspiration when she just wants acknowledgment (lines 312-317): *I suppose that sometimes you feel like people pity you more and sort of in their minds just have an idea of you not really being able to do anything by yourself or just generally can't do much and people will often tell you that you are brave, amazing for going through this, but…I…I don't really want you to do that. I suppose I could just kind of wish that they would ignore the fact, I suppose, that I have diabetes. Well, acknowledge that I do have it, but not make as much of the big deal out of it.* Reported anger or frustration at diabetes in terms of exclusions in school and family impact when having to wait for BGs to stabilise to eat (lines 296-301): *sometimes I feel frustrated with the diabetes when I'm I’m excluded I suppose from physical activities like in PE at school and I see everyone else going off to do it. But I have to stay behind because of my blood sugar and umm sometimes I get annoyed when my blood's just seem to just keep on spiralling up and down our control and people and were often waiting to eat, and my sister or my dad will often get to eat their food and I have to watch them eat it but I can't eat it because of my blood sugar.* Noted that life is much more restricted since diagnosis (lines 290-291): *definitely everything, including including everything to do with food, has been a lot more restricted nowadays.* | Discussed again the constant need for management (line 558): *The relentlessness of it* with diabetes and the ever-demanding nature of the illness with a lack of consistency or patterns (lines 495-499): *it's (anxiety) usually around over analysing or not over analysing, maybe just analysing the right amount of you know why? Why has this gone wrong? Why? Why did this come up? Why did this go down? What? Where did we go wrong? And frustration. And we don't, you know, even with the pump, we still have lots of scratching our heads.* Further level of concern as adolescent has dual diagnosis of Autism (lines 516-517): *she manages fairly well, but it it definitely does complicate managing a complex disease like type one.* | **Parental ongoing concerns but diabetes as acclimatising into adolescent identity**  Adolescent reported on awareness of how her life would change at diagnosis but noted overall it hasn’t been too bad. Wishes for acknowledgement of the load of diabetes but not idolisation or pity. Parental concerns relate to management and the responsibility still on their shoulders with the added worry of how transitioning to self-management will go due to additional needs. |
|  | **Diabetes visibility** | Reported on the visible nature of diabetes technology and the scare the invisible illness leaves behind physically (lines 321-324): *there used to be tiny little bumps or not and like pricks from all on my legs when I did the insulin pen and there are little tiny little pop, pop and when I go in the world there you can see tiny little pocket marks of where I pricked my finger of the finger pricker on my fingers.* | Reported on the aging process being sped up because of diabetes (lines 572-578): *I would say probably quicker than I would have thought …You know, a more rapid change maybe than I would have expected…ehm …And also you know self care I suppose not having as much enthusiasm for it as I should.* | **Visible invisible illness**  Both reported on some aspects of diabetes as seen whether in personal aging or technology. |
|  | **Other** | N/A | Overwhelming amount of information post diagnosis. Acknowledgement that living with diabetes is very different to the parental perspective. | **Learning required**  While the adolescent had no additional comments the mother noted the extreme learning curve with diagnosis and how overwhelming that time can be. The mother also highlighted that living with diabetes versus being the parent of a child with diabetes must be vastly different. |

|  | **Theme** | | |  |
| --- | --- | --- | --- | --- |
|  | **Illness Representations of Diabetes** | | |  |
| **Dyad** | **Subthemes** | **Adolescent** | **Parent** | **Dyadic Code/Summary** |
| **2** | **Diabetes Awareness** | Some awareness surrounding the management needs of diabetes and need for artificial insulin. No mention of the pancreas or auto immune condition (lines 11-13): *I think it's like you have really high blood sugar and you have to take insulin a lot of times. And and ehm fingerpicks a lot of times because the It's like really important to (pause) calculate what it is.* However, lack of awareness over cause of diabetes with personal diagnosis as overshadowing (lines 34-36): *sometimes anxiety or nervous. I think that's how people get type one diabetes. I'm not really sure.* However, noted serious nature and lifelong aspect of condition and hoping there might be a cure one day (lines 38-39): *kind of forever cause ehm when I was asking about how, when will, when will this end? My mother said that it doesn't really end* | Good awareness of the condition as auto-immune related and the need for artificial insulin (lines 12-13): *Autoimmune disease really… Basically ehm the virus or genetics can trigger it. Ehm I ehm and your immune system attacks the pancreas cells that produce insulin.* Awareness of the serious nature of the condition and the potentially fatal complications if unmanaged but not letting that knowledge scare her (lines 52-53): *it is serious because if you don't manage it (laugh), it can kill you pretty quickly, but also long-term consequences if it's not managed properly.* | **Parental shielding about diabetes**  There were differing levels of information between the dyad regarding diabetes awareness. Adolescent discussed the details of management but alluded to psychological causes of the illness and seeking information from his mother. The mother noted a more informed awareness of diabetes and its complications suggesting potentially a level of shielding the adolescent and primary parental management. |
|  | **Emotional Perceptions and Identity** | Unsure about the impact of living with diabetes and his experience of such (lines 25-27): *I don't really know actually, … I think I don't know how to answer that question.* However, noted the unfairness of his diagnosis and how he believes he will adapt to it soon (lines 72-77): *Well, not really fair, not really fair but not to be honest, it's not really fair, but it is a okay because like I mean, I could get used to waiting for us sometimes each like 4 meals a day because I need to like wait, I need to like, wait for the food to get digested, because when I didn't have diabetes, I just had to eat all of it at once (laugh), and I couldn't I was just still eating. And then after that one diabetes occurred I had to like, ehm I think I will get used to it soon* | Parental positivity and not allowing diabetes to stop her child doing anything in life (lines 16-17): *I don't see this as a something that will stop him in life to do anything, really. It just means that we have to do it in a different way.* Discussed the mental toll of diabetes management and needing to plan ahead for everything while not letting it burden her (Lines 59-65): *it's probably uh three or four times more planning ahead and thinking ahead of things…And just I feel that I have to be 100% engaged with what's happening. So, ehm yeah, it put a you know, more pressure on me ehm but I don't feel it as a burden.* Discussed how diabetes is at forefront of the mind and trying not to become anxious about the daily management (lines 72-74): *I think that's the first thing that I'm thinking, you know, and I'm thinking diabetes. And I'm not as worried on the Day-to-day managing things or correcting things, but more long-term.* Mother noted worrying about her child now entering puberty and wanting to blend in and the potential effects this might have on diabetes management and how this could be avoided with technology (lines 90-91): *Emotionally and blending in and not thinking that he has restrictions.* Noted that as a parent she would change it for her child she would (lines 99-102): *you know if I could change it, I would really (nervous laugh).For him, not for me necessarily, but for him. Ehm, but at the same time. I don't know, to be honest… ehm it gets me emotional* | **Diabetes as a life of restrictions**  The adolescent reported on the unfair nature of being diagnosed and hoping to adapt to life with diabetes in the future. However, the adolescent also reported on mealtimes and having to wait to eat and carb counting as aspects to adjust to. Similarly, the parent discussed at numerous points the mental demand of parental management and while she noted that she doesn’t want diabetes to hold her child back she did report on the significant amount of restrictions he will face (lines 90-96): *not thinking that he has restrictions. …A lot of restrictions. We did try to make the restrictions, you know, and also you know reward the restriction in a way…yeah.…But they are still restrictions you know?*  OVERLAP WITH IMPACT ON FAMILY LIFE |
|  | **Healthcare Orientation** | | |  |
| **Dyad** | **Subthemes** | **Adolescent** | **Parent** | **Dyadic Code/Summary** |
| 2 | **Attitudes towards healthcare** | Noted didn’t think about his health prior to diagnosis. When asked about healthcare staff experiences the adolescent acknowledged they were all “fine” but noted he was still struggling to come to terms with the diabetes himself (lines 101-102): *If it's not really that good to have ehm to be diagnosed. But I'll have to get used to it soon* | Grateful to the hospital team for their care for both their child and themselves during the stressful period in ICU (lines 164-167): *Very good care…very considerate as well, not only towards Ben and the emergency that ehm he was in DKA and they had to treat that…But also with us. Asking us how we are feeling, trying to teach us, train us.* | **Adolescent Internal focus versus Parental gratitude**  The adolescent had no real opinion about the quality of healthcare received instead focusing on his own attitude towards diagnosis and adjusting to life with diabetes. The parent however reported gratitude to the team and positive experiences with healthcare. |
|  | **Diabetes Management** | Noted that parental management is important for young children who don’t really understand diabetes (lines 85-87): *if you’re older or old enough then you could take care of yourself…But if you’re like young then yes parents might need to take care of you.* The adolescent reported a lot of support and practical help through mainly parental management (lines 92-94): *if it’s like carbs or something, when it like has to be a meal, my mother has to like count, or calculate the carbs, and what I have to do if the blood sugar is low, is I ask her, then I do a fingerprick and so on.* Went on then to reference wanting independence and knowing he should be transitioning to self-management at his age (lines 106-107): *But I think I would like have to do it by myself because I'm I'm a bit older, so I have to like, take care of myself.* | Mother as primary caregiver and responsible for diabetes management. Noted spouse and adolescent rely on her practically and emotionally (lines 139-152): *ehm yeah it mostly falls on me, but I have to say I’m not alone in it. Michael (husband) is a great help, he is also engaged, but I think he also leans on me? (laughs)…I’m the expert now (laugh). And the same with Ben, I do feel the base, I I’m definitely the go-to person when it comes to diabetes at the moment but I I do work with Ben as well to kind of introduce him into you know realising signs of a low, what he has to do…And he does, he knows these things but he still wants to rely on me. So, yeah definitely feel that I am the go-to person and I have this responsibility at the moment. Ehm I’d do more if I have to, to be honest…As a mam you don’t feel it as a you know, a burden, you’d do even more if you, you’d do anything you need to do basically.* Also noted appreciating the healthcare team for allowing her to change rates as required given the differences between hospital and reality (lines 179-180): *I appreciate that very much really because doctors know the disease, and everything behind it …but I know my son* | **Readiness for self-management**  The adolescent reported wanting more independence with his diabetes while reporting primarily maternal management. The mother reported on being the family “expert” and supporting both her son and husband practically and emotionally. However the mother did note that she is starting a slow transition by building awareness with her son. |
|  | **Future expectations** | Didn’t really have much to say about expectations- spoke more about parental management and practical aspects of managing diabetes at present but did report an honest lack of awareness about the future care (lines 41-42): *But if I'm older, I don't know if there…If this is true, but I could have an antidote or something. I don't know yet.* | Reporting hope for the future with diabetes and not letting it hinder her child’s life. Noted her positive attitude and modelling how to preserve (Lines 203-210): *I’m not at all fearing the future for Ben…Not at all. Because (pause) I thought to myself when we found the diagnosis, I said I’m going to turn this into a positive for him. Ehm because, he needs to know that he he can do anything he wants to…And, and I’ll show him how to do it* | **Future as uninfluenced by diabetes**  There was a lack of expectations reported by the adolescent combined with the parental perspective of diabetes not hindering anything in life. Suggesting a commonality of diabetes not affecting the future. |
|  | **Information seeking** | Didn’t report on information seeking. | Highlighting informed management as her need for information seeking but noting not researching potential complications and just focusing on the present day (lines 230-234): *So I try to you know be as informed as I can when it comes to diabetes, what it means for his day-to-day life, teach him how to set correct boundaries for himself but not to become fatalistic about you know…What might happen in the future.* Parent also noted the differences in information seeking about treatment options versus future complications (lines 248-249): *we read about improvements or new research findings for sure a lot more engaged probably in this side of things than reading about what might happen.* | **Parental treatment seeking**  While the adolescent didn’t report any information seeking himself the mother reported he looks into technology and sees the pump as the next step in his care. The mother reported as a family they are more focused on researching management options and opt out of researching potential complications that might never happen. |
|  | **Impact on the Vocational Environment** | | |  |
| **Dyad** | **Subthemes** | **Adolescent** | **Parent** | **Dyadic Code/Summary** |
| 2 | **Vocational Performance** | Reported on distraction post diagnosis and feeling left behind curriculum wise (lines 149-150): *Sometimes the care from teachers because I didn't really know what was happening. I didn't really know what they were talking about.* | Noted her job performance was affected initially as her child was fully reliant on her with no hypo awareness but noted a really supportive work environment that meant she could work through this (lines 263-265): *I felt that I had to be fully engaged there (with diabetes) and even in my one-to-ones with my manager I would say “you know I know I wasn’t able to give a hundred percent”.* | **Initial reactive distraction post diagnosis**  Across the dyad both mentioned initial patterns of reduced attention or concentration respectively though both noted it lessening since that initial period. However, the mother noted still feeling a mental demand of diabetes just to a lesser extent. |
|  | **Vocational Values** | Reported school as really important and noted only school goals amended post diagnosis as relating to physical activity and contingency planning for his BGs (lines 170-172): *Not really, but we did change something about like if it's PE or if it's a long walk or if it's a tour we have to like ehm calculate the blood sugar. If it's below 7 then that's not good. We have to like, drink a bit of a juice and if it goes like a…over 7 then we're good.* | Noted career progression is less valuable now as priority is on child’s health but noting job is still important to get her time outside of the house (lines 293-301): *What I think is not as important at the moment is career progression because I’m thinking my priority now is making sure that Ben becomes independent and when that happens I’ll just be in the background, nagging, the nagging mam (laugh) ehm but then I could think of career progression or changing jobs or doing something you know more exciting, because you know that will really require a lot more engagement from me and I can disengage from Ben. Yeah so career, so my job is definitely important as it provides me something outside of home and…But career progression is probably not as important at the moment.* | **Integrating Diabetes into Vocational settings**  Both reported having to adapt with diabetes with creating contingency plans for going low in school, or placing career progression on the long finger for the time being. |
|  | **Impact on the Home Environment** | | |  |
| **Dyad** | **Subthemes** | **Adolescent** | **Parent** | **Dyadic Code/Summary** |
| 2 | **Impact on Relationships** | Reported good relationship with mam and a light-hearted relationship with dad. No reported impact on communication. | Reported a closer relationship since the diagnosis noting that he always relied on her and this has become more prominent with the diabetes (lines 326-329): *he’s always been a mama’s boy (laughs) really…But I think now even more. Ehm and not becau…well he does depend on me with a lot of the diabetes related things although I am working on that with him.* With her husband she noted how the diagnosis strengthened them as a team (lines 349): *definitely brought us closer really.* Also reported an overall increase in communication in the home. | **Strengthening relationships**  Both members of the dyad reported positive relationships with each other. The mother went into detail of how diabetes has led to an increased bond or closeness with both her child and her husband since the diagnosis. |
|  | **Quality of Support** | Reported parental support when they aren’t busy while noting wanting to do things himself. However, also reported a reliance on parental management and seeking parental reassurance or guidance around BGs and duties at home (lines 203-206): *what I do I would just ask if it's okay like for example if it's somewhere around 4, should I should tell, tell them if, Should I do it? And if she said no then I listen. But she said yeah you can keep going. Then I should listen because it depends if it's like really low or not. If it's like a serious one.* | Family support is there once she requests it (lines 376): *if I’m vocal about it.* Noted preference to keep diabetes care within the immediate family but gets practical support from wider family once a year when in Ireland (lines 390-392): *I actually prefer if I do it…With my husband, you know the two of us.* | **Emotional versus Practical Support**  The adolescent noted the emotional support and reassurance he receives from his mother while the parent reported on keeping diabetes within the family unit. |
|  | **Impact on Family life** | Discussed about the food restrictions and not being able to have full-sugar drinks. No real awareness of any financial impact of type one diabetes (lines 238-239): *We just take it a bit easy I think on diabetes type one, because if it’s like I don't think we're spending a lot of money on it*. | Acknowledged how diabetes has affected the house in terms of food restrictions or familial avoidance of certain foods (lines 365-370): *you know it changed our diets…In a way that ehm that I prefer avoiding things that are difficult for me to count carbs or…You know stuff like that but yeah (pause) absolutely there is an ehm impact.* Reported no impact on finances but noted eating even leaner since the diagnosis. | **Food restrictions**  Both members of the dyad reported on food restrictions as being the biggest impact on family life. The adolescent reported on the lack of sugary foods at home since his diagnosis while the mother discussed avoiding foods for carb counting purposes.  OVERLAP WITH EMOTIONAL PERCEPTIONS AND IDENTITY |
|  | **Extended Family Relationships** | | |  |
| **Dyad** | **Subthemes** | **Adolescent** | **Parent** | **Dyadic Code/Summary** |
| **2** | **Extended family communication** | Reported on Grandparents checking in post his diagnosis but no real change in communication noted (lines 256-258): *when I came back from the hospital, I think we were chatting with the, my grandparents when I came I think that’s all like I did chat with some of my friends when I felt better.* | Affect of pandemic on communication as well as diabetes diagnosis. Noted parents having to manage grandparent stress relating to diabetes (lines 430-431): *they do want to know how’s Ben and they are very worried about very you know ehm bad consequences.* While interest levels remain unchanged the socialization had to adapt with diabetes (lines 452-456): *Yeah we look forward to meet with them (pause) they know we have to do things differently…They are accommodating. And you know we try to make it as normal as possible.*  *.* | **Grandparent diabetes check-ins**  Both members of the dyad referenced the grandparents as having checked in post diagnosis and from the parental perspective from having a lot of worry about the illness. Diabetes was noted as requiring some minor adaptations for the regular family contact to continue. |
|  | **Extended family quality of support** | Reported on mutual support from his grandmother and the awareness of other types of diabetes (line 268): *Yeah, I also support my grandmother because she has diabetes, but it's type 2.* | Noted a good support network is available to them should they need it but reiterated keeping diabetes within the family unit (line 465): *I know we have a good support network if we ever needed it.* Reported a great appreciation for family since the diagnosis (lines 477-482): *I think it actually improve, improved in a way that ehm in a way that I don’t know, made me appreciate more my relationship with them? Ehm but not in a sense that I am opening about “this is so hard” this is not for that, this is just that I am thinking that I have these great relationships and I should cherish them.* | **Newfound family bonds**  The adolescent reported on mutual support gained from his relationship with his grandmother while the mother commented on the overall appreciation for her family she has since the diagnosis. |
|  | **Social Environment** | | |  |
| **Dyad** | **Subthemes** | **Adolescent** | **Parent** | **Dyadic Code/Summary** |
| 2 | **Impact on interest levels & participation** | Reported no real impact on hobbies of any sort but noted any changes would be due to outgrowing hobbies rather than any influence of diabetes. | Parental loss of personal time since diagnosis though interest levels unchanged (lines 499-502): *maybe not as interested. Ehm I still enjoy them…Really. Ehm I just feel I don’t have as much time to do to as I used to.* Reported a bigger effect from the pandemic than diabetes on changing family activities. While some initial concern over physical activities with diabetes it has all been fine so levels unchanged. Noted any activities with her spouse are more difficult due to only leaving their child with trusted family members due to their concerns (lines 543-544): *You know, cause I know I I prepare ahead so I know Ben will be safe and I am okay to be far away from him.* | **Parental social engagement adaptations**  While the adolescent reported no effect of diabetes on his social life the parent highlighted the mental toll and responsibility of parental management as affecting hobbies. This was both in terms of time and mental energy to engage in hobbies as well as fear when leaving her child. |
|  | **Psychological Distress** | | |  |
| **Dyad** | **Subthemes** | **Adolescent** | **Parent** | **Dyadic Code/Summary** |
| 2 | **Mental toll of Diabetes** | Reported on initial fear around diagnosis and lack of knowledge if it was terminal or not (lines 347-350): *The diabetes I think because I was really anxious about that. Like I was going to die (pause) or something. I didn't. I thought I was going to. But no, that didn't happen.* Reported on a reactive depression when in ICU post diagnosis following his coma. Also commented on his emotions affecting his BG levels (lines 382-382): *Sometimes I do worry because if it's like low then I just because sometimes if I'm like going back to school it just goes high because I'm a bit worried about it.* | Commented on diabetes anxiety post diagnosis while getting to grips with the illness. Reported it now as second nature and less anxiety provoking when there are BG fluctuations (lines 566-568): *It did at the beginning really when I didn’t know what I was dealing with but the more I learn, I learned, the more diabetes becomes like second nature. You know I don’t get worried when I see his trend going high or going low or whatever*. Mother noted guilt at the diagnosis in terms of not preventing the illness but of preventing the extent of his sickness and bringing him into hospital sooner. However, parent noted positive coping and living in the present (Lines 591-596): *absolutely, theres the odd moment when I try, or you know I feel tired or overworked or…Overwhelmed with how many things I have to do, and I might feel weak and blame myself but I always am able to, to* *realize I am* *when it’s happening and go “no no stop”* | **Diabetes and Anxiety**  Both members of the dyad reported anxiety as the main emotion they experienced related to diabetes. The adolescent noted lack of awareness at diagnosis and then normal everyday emotions now triggering fluctuations in his BGs. The parent noted initial reactive diabetes while adjusting to life with the management. |
|  | **Diabetes visibility** | No physical changes reported associated with diabetes. | No physical changes reported associated with diabetes. | N/A |
|  | **Other** | N/A | N/A | N/A |

|  | **Theme** | | |  |
| --- | --- | --- | --- | --- |
|  | **Illness Representations of Diabetes** | | |  |
| **Dyad** | **Subthemes** | **Adolescent** | **Parent** | **Dyadic Code/Summary** |
| **3** | **Diabetes Awareness** | Good awareness of diabetes as relating to lack of insulin production (lines 7-9): *it’s when your pancreas stops working, it shuts down and your body stops producing insulin so ehm you either have to inject yourself or get a pump to give yourself insulin.* Commented on awareness of genetic component and unknown element for lots of individuals (lines 18-19): *I’m not sure really if it’s a genetic thing or…(pause) if it’s just an unfortunate thing that just happens, that you can just get it out of the blue.* Noted awareness of potentially fatal nature if unmanaged. Described life with diabetes as containing food restrictions and being mindful of physical activity (lines 36-38): *it just affects the meals I eat, I’m not allowed to have too much sugar or I just have to be cautious of running or doing loads of sports- I still do them but just keep an eye on myself.* | Good understanding of diabetes and auto immune condition (lines 16-17): *his pancreas, the islet cells were wiped out by an autoimmune reaction and he is now insulin-dependent. So his body doesn’t create insulin.* Diabetes management as key to a good life (lines 26-27): *It means he has a lifelong chronic illness that has to be constantly managed and maintained.* Commented on the genetic component or potential triggers for the illness with awareness of no known cause or way to prevent it (lines 31-33): *It was an autoimmune reaction, it was nothing we did or he did or …it was just something that he is predisposed to, and it happened like a switch went and that was it. We don’t know why.* Awareness of the potentially fatal nature if left untreated (lines 45-47): *Well, if it’s not managed, it’s as serious as it can get, it can kill him so… It’s life threatening, it’s not life limiting but it’s life threatening.* Life with diabetes was described as requiring never-ending management and lots of forethought (lines 52-58): *It’s just a lot more organisation, a lot more thought…ehm planning, to ehm having his prescriptions, his appointments, having all his needs met, as in having all he needs to deal with his diabetes…Always there. And ehm food ehm yeah you know he always had a pretty good diet it’s not changed drastically or anything but you just have to have that little bit of extra thought that there’s no carefreeness anymore.* | **A Life lacking carefreeness**  Both members of the dyad touched on having to be mindful and purposively thoughtful about a lot of life now with diabetes. Parent noted the life admin associated with management and supplies restocking while the adolescent noted the day to day thought required with everyday activity. |
|  | **Emotional Perceptions and Identity** | Noted an initial apprehension after diagnosis but not letting diabetes stop him now (lines 14-15): *when it started I felt kind of limited in what I did and then I realised that it doesn’t really change that much except you just have to be cautious of some things.* Reported diabetes as being more manageable and therefore less of a priority for a cure than other illnesses (lines 26-27): *it is important but I feel there is other more important things that could be researched and developed before diabetes.*  Reported diabetes as a normal aspect of life now (lines 42-45): *it is a lot easier than you think it would be… Ehm I don’t really mind it’s just a normal thing now…* | Parental sadness over diagnosis and wishing to have been the one diagnosed (lines 68-74): *yeah, I’m sad, I’m sad for him. I’m sad that he has to deal with this for the rest of his life, I would take it for him…*  *Interviewer: Yeah.*  *Interviewee: …But unfortunately as his mother I cant. It’s just, so I just have to give him the tools to deal with it and hopefully he will live a full and happy life. Ehm I cant do anything to take it away, I cant do anything to make it better, but I can teach him how to life a full life, and not let it affect him or hold him back.* | **Diabetes as a normal part of life**  Both members of the dyad highlighted that while there is an emotional load with diabetes, that it will not limit the adolescent’s life. While the parent reported wishing to take away the diagnosis there was also an understanding of not letting diabetes define you or limit you which the adolescent appears to have inherited. |
|  | **Healthcare Orientation** | | |  |
| **Dyad** | **Subthemes** | **Adolescent** | **Parent** | **Dyadic Code/Summary** |
| 3 | **Attitudes towards healthcare** | Diabetes making him more health conscious (lines 57-58): *I’ve realised that well I do know a lot more about health and all that.* Noted really considerate and understanding healthcare team that made diagnosis less daunting (lines 75-76): *and I feel like everyone understood that I was going through a difficult time…* | Fairly health conscious prior to diagnosis. Great experience with the healthcare team (line 129): *I literally couldn’t say enough good things.* Reported staff as the one good thing in the bad situation. Healthcare team as very proactive with technology as well which was hugely beneficial to management. | **Healthcare light in diagnosis darkness**  Both members of the dyad recounted the really positive experiences with the healthcare team that stood out positively during such a difficult period. |
|  | **Diabetes Management** | Constant management required for stable diabetes – noted monitoring of BG fluctuations as integral aspect of routine (lines 65-69): *well I have to constantly check my bloods, with my blood glucose, check, prick…And ehm just make sure that everything is steady and if I’m going to high, to give myself insulin and if I go low to make sure that I don’t go completely, down.* Technology regarded as making diabetes management easier (lines 81-82): *all the technology and stuff really helps, that I don’t have to do injections and all every couple of hours, every meal and that.* | Parental management has changed since the initial time of diagnosis thought still same level of parental responsibility and level of oversight in all activities (lines 100-101): *I suppose when he was diagnosed I had to go to all the birthday parties and I had to be sitting outside, I still do, I, I what I do now is I get involved in his activities…* Noted the parental concern at transitioning to self-management (lines 108-112): *You have to let them go, it’s really hard, it’s one of the hardest parts about it …is letting go of your control over the situation and handing it over to them…But he has taken over a lot of control and I’ve had to let him…so it is …it’s a little easier on me now.* Noted the level of this fear means that she still hasn’t left him over a weekend (line 124): *I haven’t gone away for a weekend and left him yet.* | **Management as never-ending**  Both the adolescent and parent commented on the constant need for monitoring and management with diabetes. While the mother discussed the transition from parental to self-management and the difficulties with this passing of responsibility, the adolescent noted that technology makes the everyday management easier. |
|  | **Future expectations** | No real expectations for his future but noted diabetes did affect his life at the time of diagnosis when getting to grips with diabetes (lines 86-90): *I don’t really know, you just, I just (laugh) move on with my life, like I still do everything that I used to do, I still, like I stopped doing things for a while, I stopped playing sports and that…But I got back into it and I’m really happy that I did.* | Knowledge of the evolution of diabetes care and the drastic advancements in technology in recent years leading to hope for a cure in the distant future (lines 174-179): *I think well I think there’s going to be more technical advancements, I mean I don’t know if I’ll ever see a cure in my lifetime, I’ll be honest I don’t know…*  *Ehm you always wonder about these things, but you have to look at that insulin is only 101 years old and the difference in a hundred years is phenomenal* | **Hope and Nonchalance**  There were differing views expressed about future expectations for diabetes given the adolescent noted not thinking about his future while the parent noted being hopeful for a cure given the advancements in diabetes care thus far. The causal indifference from the child suggests a good level of acceptance or adjustment to diabetes.  OVERLAP WITH INFORMATION SEEKING |
|  | **Information seeking** | Noted does some information seeking about cures but trusts the healthcare teams opinions regarding current treatments (lines 100-103): *I’m happy with what the team tells me but I have looked up like on google and stuff, just to see how the progression on cures and that is going cause. It would be nice to know that you know, it could be gone (laugh) one day.* | Very curious mind always researching and advocating for better quality of life (lines 199-201): *Yeah, I’d be pushing for the next advancements yeah. Whatever he can have to make his life manageable and easier is what I want.* | **Future versus present information seeking**  Adolescent noted information seeking about a cure in contrast to saying he didn’t think about his future, while the mother noted focusing on present day research and making life now as manageable as possible for her son.  OVERLAP WITH FUTURE EXPECTATIONS |
|  | **Impact on the Vocational Environment** | | |  |
| **Dyad** | **Subthemes** | **Adolescent** | **Parent** | **Dyadic Code/Summary** |
| 3 | **Vocational Performance** | School performance affected by lows and the need for treatment- noted lack of concentration and ability when low (lines 108-109): *like if I were to go low in the middle of class I would leave the class to go treat myself cause I would be weak, and I wouldn’t really be able to do much.* Also noted how technology is fallible and the impact of failed technology on missing school days (lines 120-124): *there was maybe one or two days where my bloods, where I woke up high because there was an issue or something with my set, or my pump, where my bloods stayed, so my insulin wasn’t being delivered…*  *So then my bloods would go high and then I’d have to take the day off because they just take a while to go down.* | Noted initial reactive separation anxiety from the child to mother. Mother as primary care giver and having to give up work in order to get to grips with the constant management (lines 219-221): *Because he was so ill ehm and I couldn’t work, I couldn’t be away from him, I had to be on call all the time, so I had to cut, I stopped working for a few months and then I went back…* | **Constant management**  Adolescent reported on the constant daily tasks of monitoring and managing BG fluctuations in school and the lengthy impact these can have physically and mentally on the body. Mother reported on the initial affect on her leaving her career and eventually going back to work weekends so that there is 24/7 a parent at home for management purposes. |
|  | **Vocational Values** | Noted school is as important as pre diagnosis and no impact of his diabetes on his academic goals. | Job as important social and financial outlet for parent once adjusted for diabetes -weekend work so always one parent at home (lines 247-248): *Yeah because well it pays the bills (laughs), and also ehm it ehm it’s an outlet for me…* | **Life outside of diabetes**  Both members of the dyad noted importance of school or job respectively regardless of diabetes. |
|  | **Impact on the Home Environment** | | |  |
| **Dyad** | **Subthemes** | **Adolescent** | **Parent** | **Dyadic Code/Summary** |
| 3 | **Impact on Relationships** | Great close relationship with mother (line 146): *I love my mam. I think she is the best person in the whole wide world.* Overall reporting really close knit and supportive family unit with no reported change in communication levels. | Really close relationship with adolescent noting the time in hospital as bonding (lines 281-284): *I suppose like when he was sick and he was in hospital and stuff and we did, well we were always bonded like I’d be, I just would adore them all…But yeah we’d have a lovely relationship.* Noted sibling relationship as “normal” as if diabetes wasn’t in the equation (line 315): *The kids would watch him like watch out for him, but you know as much as they’d do if he wasn’t diabetic.* Reported the spousal relationship as unchanged by the diagnosis. Noted that diabetes became the main topic of conversation in the household (lines 230-231): *Oh yeah, no there was, it like the conversations changed there wasn’t as much day to day, it was “what are his blood levels now?”* | **Supportive relationship**  Both members of the dyad highlighted their close bond and good relationship. The parent went on to expand on how diabetes is just a normal aspect of life as all of the kids have grown up with it. Post diagnosis diabetes became a focal conversation topic thought with help of technology this has lessened over the years as both parents can monitor with the technology.  OVERLAP WITH QUALITY OF SUPPORT |
|  | **Quality of Support** | No major impact of diabetes on household chores but could rely on siblings for support if required (lines 161-164): *If I had to do a job and then my bloods went low I’d get one, one of my siblings…To do it for me just because they would understand that I was low.* Also noted mother as primary caregiver and main source of support at home (lines 176): *If I did need help, mam would definitely do that for me.* | Noted as primary caregiver majority of the diabetes management falls on her. No resentment towards husband he supports as he can but understanding of the dynamics within the management system (lines 335-336): *So it was very much that I would be the primary caregiver because my husband works very long hours.* Noted not wanting to burden the wider family with diabetes though noting her own mother (grandmother) as great source of support (lines 392-393): *she did, she learned how to do injections and everything…* | **Dyadic support**  Adolescent noted that his mother is his main source of support at home with the mother echoing that she is the primary caregiver. This level of maternal responsibility was also highlighted in the fact that she didn’t wish to ask anyone outside of the immediate family for help. Therefore, this highlights the close bond between the dyad as outlined above.  OVERLAP WITH IMPACT ON RELATIONSHIPS |
|  | **Impact on Family life** | Noted the positives of the LTI scheme in Ireland (line 185): *Like we did get the pump and all of that for free.* | While more tired around time of diagnosis with juggling life and diabetes noted that it everything within the home had to be taken care of as well so forced to adapt (lines 359-361): *Like you can’t sit under it and yeah like I had a cry every now and again, I was sad and I was upset but I, I had three other kids well four kids that need me and a home to run.* Noted trips up and down for appointments as expensive but highlighted the LTI scheme as making diabetes care affordable (lines 414-415): *Not really, no because there’s all LTI (long term illness scheme) and the only thing is coming up and down here.* | **LTI scheme for affordable diabetes care**  Both members of the dyad listed the LTI scheme as resulting in no real financial burdens to diabetes care in Ireland. Mother also highlighted that as a parent you don’t have a choice in balancing life and diabetes echoing back to her stance as the primary caregiver. |
|  | **Extended Family Relationships** | | |  |
| **Dyad** | **Subthemes** | **Adolescent** | **Parent** | **Dyadic Code/Summary** |
| **3** | **Extended family communication** | Reported good communication and no real change in interest or communication levels. Noted grandmother in particular as good support and understanding of diabetes (line 212): *Granny, yeah she definitely, she understands everything.* | Awareness of diabetes but lacking true understanding from wider family emphasizing the parental burden (lines 437-440): *people don’t really understand what it’s like unless they have it in their house, they think they know, but they don’t know. And they are like “How’s Conor? Okay-grand”. And that’s it, they don’t know, unless you are one that is talking about it all the time, and then people don’t even want to listen.* At diagnosis there was communication and checking in but again this lack of true understanding or misconceptions about reality of diabetes was noted within the wider family (lines 454-458): *Family would’ve been texting and stuff like that but people go back to their own lives…You know and it’s not, because people don’t see it as…(pause) ehm ba-they don’t think it’s bad you know?* | **Understanding perceived as true or not?**  Differing views reported in terms of the adolescent noting the grandmother as really supportive and understanding of diabetes even learning how to help administer injections. However, the mother reported a lot around superficial check ins from family and a lack of true understanding or the awareness of the serious nature of the condition. Mother highlighted that when she was managing it all well, family nearly dismissed how hard it was as they didn’t see the work. |
|  | **Extended family quality of support** | Grandmother as great support and helping to share the burden of diabetes (line 199): *And we taught granny how to do everything and she immediately understood.* | Parental fear post diagnosis and not wanting to burden the wider family (lines 480-482): *we probably didn’t go anywhere for a while, we didn’t leave him…For a year or two.* Grandmother as good source of support but rarely called on for not wanting to burden others. | **Grandmother as trusted support**  Both highlighted the maternal grandmother as a great source of support but the mother noted still not wanting to call her for help for fear of burdening others. |
|  | **Social Environment** | | |  |
| **Dyad** | **Subthemes** | **Adolescent** | **Parent** | **Dyadic Code/Summary** |
| 3 | **Impact on interest levels & participation** | Noted diabetes initially stopped participation in physical activities while getting to grips with the illness but now more involved and interested in fitness because of his diabetes (lines 238-239): *I’d say I’m more interested because ehm I’d say like with football like I’d like to keep on top of you know my fitness.* | Diabetes as impacting work schedule (weekdays to weekends) meaning shift in timing of hobbies or adapting to new routines (lines 522-523): *He would ehm, he’d come home and I’d go, I, I, I made it work that I did different times or whatever.* | **Reactive adjusting of social activities**  Across the dyad noted initial changes in routines with hobbies and social engagement to allow for one or other parent to be supervising at all times or to allow for a learning curve with diabetes management. |
|  | **Psychological Distress** | | |  |
| **Dyad** | **Subthemes** | **Adolescent** | **Parent** | **Dyadic Code/Summary** |
| 3 | **Mental toll of Diabetes** | Initially quite anxious following diabetes diagnosis but has now gotten to grips with life with diabetes (lines 278-279): *I was when I was like younger but now I’ve like gotten better and like going places Iand like realising like that everything will be okay.* Noted his adjustment to diabetes over the years with less overall worrying, less anger at parents and generally getting along well with his life now (lines 300-301): *I worried for the first couple of years when I was…but then I started getting less worried and more confident.* | Noted technology as great when working but found it to be fallible leading to high stress and increased parental engagement levels and life admin to try source a new one (line 570): *well his pump failed there about a month ago- I was very stressed.*  Noted the parental burden and concern never goes away and there is more new experiences as he ages (lines 623-628): *Yeah, say he went to a disco the other week and I, I just don’t think I breathed (laughs) the whole time he was gone…And he went to a friends for an overnight stay which he has only done…this is the second time.* | **Positive adjustment versus consistent parental concern**  Opposing viewpoints as adolescent reported on initially experiencing high levels of anxiety and anger post diagnosis and coming into his own now with diabetes versus the parent reporting ongoing concern over the loss of control with transitioning to self-management and letting her son grow up. Overlapping here with emotional perceptions above. |
|  | **Diabetes visibility** | Some small visible scars from placing technology under the skin but no major visible features to the diabetes (lines 308-309): *I’ve a couple s… scratches and marks and stuff say from where I put my sets and that but other than that it’s grand.* | Noted no perceived physical affect of diabetes on appearance. | **Technology as leaving marks**  Adolescent reported on some small marks left from technology making diabetes slightly visible while the mother didn’t comment. |
|  | **Other** | N/A | N/A | N/A |

|  | **Theme** | | |  |
| --- | --- | --- | --- | --- |
|  | **Illness Representations of Diabetes** | | |  |
| **Dyad** | **Subthemes** | **Adolescent** | **Parent** | **Dyadic Code/Summary** |
| **4** | **Diabetes Awareness** | Reasonable understanding of diabetes as related to the pancreas but no mention of insulin production (lines 16-18): *Type one diabetes is when I believe a whole infection infects the pancreas and the immune system does not ehm spare the pancreas it, it just it just it's it's, it's ehm where the immune system kills the pancreas.* Used humour to deflect from the lifelong nature of the condition (lines 34-35): *well for me you should probably last eight years because that's when the… sorry. No, sorry actually that's a that's a bit of a cheeky answer.* Recounted the physical regime and carb counting management required for daily life with diabetes noting the physical pain also associated with technology insertions (lines 61-68): *Like with the school menu- have them custom weighed so you know I don't, so I know what to Bolus is that like I have. Like, if I'm going to like a restaurant, I do have to bolus. Like I I also like every two days. Have to… Ehm… Reinsert my I put it in a new set…(pause)…And every seven days a new sensor…Which is usually pretty painful.* | Good awareness of diabetes and the need for artificial insulin (lines 10-11): *type one is basically where your pancreas stops producing insulin. So ehm you need to take in some form, injections or if you have pump, to match anything that you’re eating and then you need basal insulin to keep your body functioning between meals.* Noted the serious nature of the condition and the need for consistent management (lines 114-115): *I would say it's very serious and as that, but if you're managing it appropriately it can become less serious.* Highlighted due to husband’s job knowledge of long-term complications and awareness of hypers as being as serious as hypos despite healthcare teams emphasizing hypos (lines 123-125): *we know the, the long term effects of staying high for, for quite a long period. So we take highs as seriously as we take lows.* Noted the daily parental management has lessened slightly since diagnosis though highlighted the daily texts with SnA’s and monitoring BGs even while in work (lines 157-161): *It's becoming more just part of the ro-routine, like the last year or two, I mean, at the first few months it was very intense…And now it's becoming just more automatic. You know, that it's part of our routine.* | **Diabetes management as key**  Both members of the dyad highlighted the level of management with diabetes as nearly consistent and extremely important in order to keep healthy. The parent acknowledged the potential long-term complications associated with poor management but noted that diabetes is just now a part of the daily routine. The adolescent used sarcasm when discussing diabetes before reverting to a informed answer about diabetes progression noting again the need for good management. |
|  | **Emotional Perceptions and Identity** | No real awareness of personal feelings towards living with diabetes (lines 23-24): *I don't really have any thoughts on… this with like I don't really have any thoughts on…the whole thing in general like.* While discussing the severity of the illness when untreated he noted that living with diabetes can still be a “normal” life (lines 40-41): *Very serious like, you know, your pancreas is stopped. You no longer naturally produce insulin, but you know …you can live a normal life.*  Highlighted some slight frustration at having diabetes but again no real emotional reaction to his diagnosis (lines 81-85): *I don’t have, have big feelings on the topic…Just like maybe just a slight annoyance…Just a bit miffed about the whole situation.* Later noted that diabetes had changed him but less in an emotional manner and more from a maturity perspective (lines 90-93): *Like if, like to be honest, being diagnosed probably changed a lot about my personality…And maturity levels.* | Shock at diagnosis but noting immediate need for knowledge and understanding so that diabetes fit into their lifestyle rather than vice versa (lines 22-26): *So the diagnosis is obviously a shock to any family and it was a shock to our family…But our key is very much. Figure it out, learn how to manage it and it just falls in with, with our life.* Noting pride at her son for his knowledge of diabetes from reading and being the one to spot the symptoms (lines 82-83): *And David said, “Well, I'm not, I'm not a doctor, but if I were a Doctor” he said, “I would say that I've got diabetes”.* Life pre-diabetes as hard to remember now that it has become part of daily routine (lines 180-181): *it’s nearly harder to remember when he didn't have it?* Commented on the need for individuals with diabetes to vocalise and acknowledge the emotional toll of diabetes (lines 196-198): *by being able to talk about it when they want to talk about it …ehm and having crap days where they're like, this is just, shit yeah.* Reported pride over adolescent’s resilience and attitude towards life with diabetes (lines 207-209): *only recently, where it was sore going in. But it was like, he still didn't say “oh this crap”. He just kind of complained. But he hasn't actually vocalized that this is, this is crap. Like, he's actually been amazing with it all.* Noted the unfair nature of her child having to mature sooner than necessary and the lack of spontaneity now as compared to peers (lines 215-218): *I feel like it's, you know, it's a lot for a kid to take on, and I feel like as he gets older like, I know he'll manage it and I know we're getting into the routine of it. I do feel like it's an extra kind of responsibility that takes away some of the carefreeness that he might have otherwise had.* | **Parental pride and Adolescent Ambivalence**  Differing perceptions about the emotional toll of diabetes reported with the adolescent noting that diabetes still allowed him to have a normal life so therefore reporting no real opinion on having the illness expect that it made him more independent. Whereas the mother reported significant pride at her son’s adjustment and taking diabetes in his stride noting her own sadness that he has diabetes and the loss of carefreeness in his youth. |
|  | **Healthcare Orientation** | | |  |
| **Dyad** | **Subthemes** | **Adolescent** | **Parent** | **Dyadic Code/Summary** |
| 4 | **Attitudes towards healthcare** | Noted great experiences with the healthcare team while allowing room for improvement (lines 116-118): *I mean, when I say top notch, I mean, I don't mean that literally because I'm pretty sure …(pause)…ehm…ehm, like I'm Ehm, like when I say that, I mean, you know, things can always improve like.* | Noting being extremely health conscious and reigning it back slightly in the year prior to diagnosis but noted that being health conscious didn’t prepare them for diabetes management (lines 269): *it meant that we had to learn the carbs and everything and all that.* Noted great experience with the healthcare team (line 326): *Excellent. Yeah, absolutely excellent. It's been amazing since the start.* | **Positive Healthcare experiences**  Across the dyad positive healthcare experiences were noted while the mother also outlined that having a good attitude towards health prior to diagnosis didn’t prepare for the constant need for management that diabetes requires. |
|  | **Diabetes Management** | Noted the big differences in medication management and seeing technology as easier (lines 103-104): *Bolus, like I used to also have to manually inject myself with a with an insulin pen.*  Highlighted technology as requiring less thought devoted to diabetes despite the numerous aspects still involved (lines 136-138): *I do have to reinsert the site and the sensor every set two and seven days respectively. But you know it's really quite like… The pump is really, really nice just to have, just to be able to Bolus and not having to inject myself.*  Noted three major shifts in health related quality of life (lines 140-157): *like I'd say, there's been like, three significant jumps in health and like quality of Life…When I got the ehm, when I first got the insulin pen because before that I just been diagnosed you know? Major quality of life improvement. Ehm then, when Fiasp, was uh when I started getting Fiasp instead of Novorapid* *that…Novorapid like takes 10 to 15 minutes to start acting…Fiasp, five or immediately you know? Really useful…And then, and then when I got the pump.* | Noted the teenage transition from parental to self-management particularly regarding food choices and having to hand over control (line 274): *That’s fine as long as he just manages to bolus appropriately.* However, noted that parental management including oversight and life admin is never-ending and is constantly happening in the background (lines 301-306): *Ehm so as a parent then I, it's on me to make sure that he's calibrated and, in the morning, and in the evening and then also when he's in school, so it's making sure he's equipment. So when he's in school that they are stocked with the bars and the low treatments and fingerprick kits. And then it's also for me when he's in school, even though the teacher very much manages it, as a parent, I have to be just keeping an eye that they're on top of lows and that they bolus for lunch.* Commented on the technology as great if you have it all synced up and the latest technology. Noted manual administration had its benefits as technology can be an information overload and require lots of monitoring to ensure its working correctly (lines 350-353): *then even just the fingerpricking was because the fingerprick with your injections, you forget about the sugars…Whereas with this it's much more involved.* | **Differing views on technology**  Differing views were reported on the impact of technology. The adolescent noted that while technology is still very laboursome and can be painful as manual administration was, it was still regarded as easier overall. The mother noted the handover of management and responsibility to her child with regards to technology and how she can still have parental oversight with the BG trends. This allowed for peace of mind but also was regarded as an overwhelming amount of involvement at times. |
|  | **Future expectations** | Noted hope for a cure in the recent future and technological advancements in the interim (lines 168-172): *I'm quite, I'm quite hopeful that's in like 8-9, ten years…You know, diabetes will be a curable illness but until then I expect like expect the pump systems to advance, the pumps and sensors to advance significantly.* | Noted overlap with information seeking as she reported following lots of research closely to see upcoming advancements (lines 374-377): *That all the technology like is progressing so quickly. I feel like each year there's going to be newer technology…And newer advances.* Noted hope for a cure within her son’s lifetime and to see major progression with day-to-day treatment options along the way (lines 387-397): *I'm very hopeful that by the time David in his forties potentially they'll either be much more kind of regular, kind of like whether it's pancreas and stem cell treatment, or whether it's transplant…It'll be much more commonplace. I think, so I'm quite hopeful for him that, you know, it'll be easier to manage with all the technology advances that are going to happen…And that also the actual potential treatments are, won't be long or will be in his lifetime.* | **Hope for a Cure**  There was a clear commonality in the overlap between future expectations and information seeking in this dyad. Both members reported lots of research together into potential cures and treatments and highlighted their hope for a cure within their lifetime.  OVERLAP WITH INFORMATION SEEKING |
|  | **Information seeking** | Noted no personal need for information regarding diabetes progression or complications but very interested alongside his mother in researching future treatments or potential cures (lines 192-193): *growing organs using a person’s DNA, like you know, it's probably going to replace a loss of a lot of things.* | Noted lots of researching potential cures and advancing treatments (lines 360-361): *I am following a study in the, that started in the states ehm that's in progress-It got a fast-track approval.* Commented on her rapid knowledge acquisition with regards to daily diabetes management and the subsequent researching better treatment options once she got to grips with it (lines 416-420): *then once that part was done, and once I’d the technology set up. And I felt I was able to manage the actual logistics and day to day…Then I wanted to know everything there was to know in terms of what we need to do yearly…* | **Dyadic Information Seeking**  Again as outlined above, lots of overlap as the dyad noted they engage in information seeking together and both reported on the same study they are following the progress of.  OVERLAP WITH FUTURE EXPECTATIONS |
|  | **Impact on the Vocational Environment** | | |  |
| **Dyad** | **Subthemes** | **Adolescent** | **Parent** | **Dyadic Code/Summary** |
| 4 | **Vocational Performance** | Noted exclusion from physical education due to hypos (lines 228-229): *So like, like I've had to stay back from from PE a few times because my sugars were going low.* | Noted no real impact on house duties just a reprioritisation of duties (line 473): *Well, I just didn't prioritize doing some of the stuff that I might've done.* Significant loss of time and mental energy to monitoring diabetes in daily life (lines 493-494): *Like you'll be with someone for coffee and suddenly it’s like, “I'm sorry to take this call”. Or “I'm sorry, I have to check this alert”.* On going back to the workforce noted doesn’t foresee a huge impact of diabetes (lines 518-519): *in general I feel like it's not going to be disruptive to my work apart from the odd time saying “I have to check my phone”.* | **Physical versus mental impact of diabetes**  While the adolescent highlighted on the physical exclusions he experienced due to his illness the mother reported a general loss of time and mental capacity from the parental oversight required for good management. The transition from parental to self-management was noted as lessening this parental burden.  OVERLAP WITH VOCATIONAL VALUES |
|  | **Vocational Values** | No reported change in school importance. | Going back to a job following a lengthy career break the mother reported the job as equally important now and less parental concern due to transition from parental to self-management (lines 502-503): *I'm dying to get stuck in, and I feel like the diabetes isn't going to, I think previously it would have kind of concerned me, like how would I manage.* | Same as above.  OVERLAP WITH VOCATIONAL PERFORMANCE. |
|  | **Impact on the Home Environment** | | |  |
| **Dyad** | **Subthemes** | **Adolescent** | **Parent** | **Dyadic Code/Summary** |
| 4 | **Impact on Relationships** | Reported good supportive relationship with mother and more light-hearted fun relationship with father. Noted some minor sibling jealousy over low treatments but otherwise no real impact on relationships (lines 263-264): *Apart from maybe a few times when I'm going low and I have something, ehm have something sugary and one of my siblings wants it?* No reported change in communication levels. | Noted good relationship with son since the diagnosis and him seeing a therapist for additional needs outside of diabetes (query autism) (lines 548-549): *great… you know, he's, just we were trying- not often enough, but we were trying to kind of have, like, regular check-ins.* Acknowledged lots of thought going into fairness and balancing life and diabetes with the other children post diagnosis (lines 581-585): *So we would have been, I suppose, more like kind of trying to … like a swan (laugh), so like peaceful on the top for the other kids…And things are fine, but that the underneath paddling to make do all the groundwork.* No reported impact of diabetes on communication levels.  *.* | **Whole family adjustment**  Both members of the dyad noted it was the whole family adapting to life with diabetes not just the child diagnosed. Adolescent reported some minor jealously from siblings while the parent noted the mental load of trying to balance diabetes within their lives to keep a semblance of normality for all children. |
|  | **Quality of Support** | Reported not liking asking for help related to diabetes (lines 272-273): *like I just treat my low, and ehm wait for, wait for my sugars to go back up and then get on with it.* | Good support network but diabetes as separate to that (lines 621-622): *not to do with the diabetes really. No, I mean, I already have before he was diagnosed, I had very good kind of support systems in place.* Noted finding a babysitter with knowledge of diabetes and only trusting this one woman with care for initial period to reduce parental concern (lines 624-626): *one thing I did do when he was diagnosed is I found a minder who does like babysitting or overnight, who worked at family, who had a child ehm he was diagnosed with type one.* | **Diabetes specific support**  The adolescent noted not liking asking for help and trying to be independent while the parent noted having diabetes specific support is difficult. Finding someone with a knowledge and understanding of diabetes was the only way the parent could find any comfort leaving her child.  OVERLAP WITH IMPACT ON FAMILY |
|  | **Impact on Family life** | No reported impact on household chores. Lack of awareness around financial aspect of diabetes care but acknowledged accessories (pump straps) as additional but non expensive costs (lines 299-301): *I don’t…think they are really …any demands like. Like you know, maybe you like…I don't know if insulin costs anything, but I'm pretty sure it's it's …like if I'm pretty sure it doesn't really cost anything… or much.* | Noted lots of effort required to go away with her husband. Reiterated need for one babysitting to ease parental fear. Noted the constant management and monitoring and not ever getting a break from diabetes even during a date night (lines 655-659): *even though, like I'm saying, “Oh it’s not that intense” -I'm on autopilot that I'd be checking my phone, checking the sugars…Like I'm going to do that like 100 times a day, nearly like it's not. It's just what I do.* Noted both parents stopped drinking alcohol-unrelated to diabetes but had positive knock-on effects for diabetes worries (lines 683-685): *also then with the diabetes it means like I just would never have wanted that risk, that I would have had a few drinks and then not have responded to something appropriately.* Reported no effect on finances. | **Awareness of impact on family life**  Adolescent reported no real impact of his diabetes on the family unit whereas the parent noted the constant parental management and responsibility as affecting time away from her son, her engagement with other people and requiring diabetes specific support to ease the fear.  OVERLAP WITH QUALITY OF SUPPORT |
|  | **Extended Family Relationships** | | |  |
| **Dyad** | **Subthemes** | **Adolescent** | **Parent** | **Dyadic Code/Summary** |
| **4** | **Extended family communication** | Slight change in communication due to age rather than diabetes (lines 326-327): *say like I'd be more so interested just because I've just matured -like I was ten when I was diagnosed.* | Noted lack of family or public awareness around diabetes. Lack of true understanding without living with it so leading to frustration with family members (lines 708-711): *I, I, I have, but I feel like none of them get it. None of them get it. Ehm so I have as many interactions with them, but it does lead to frustration sometimes that they've no awareness.* Finding the wider family as unsupportive as they don’t have to realise how difficult diabetes management is as it doesn’t affect them (lines 719-724): *But in the beginning I did find that frustrating ehm that people, people would be like, “Oh alright, that's bad” and you know and then they just get on with it…And they don't realize what a life changing thing it is, and …What a big impact it is on the family, you know?* | **Perception of diabetes importance**  Adolescent noted increased level of communication due to maturing with no mention of diabetes impact while the mother reported a lot of frustration at the lack of true understanding of diabetes from the wider family. |
|  | **Extended family quality of support** | Noted due to family members living abroad there is little opportunity for socialisation or getting support. | No support from family members so creating own support network (line 729): *They haven't been involved at all. But I have put supports in for myself.* | **Lack of familial support**  Both members noted lack of support from family members. |
|  | **Social Environment** | | |  |
| **Dyad** | **Subthemes** | **Adolescent** | **Parent** | **Dyadic Code/Summary** |
| 4 | **Impact on interest levels & participation** | Reported change in hobbies due to age and interests changing (line 362): *outgrowing some of the hobbies.* Also noted family members not always being as interested as he is in family activities. | Reported no diabetes related impact on social activities more so the pandemic as affecting engagement. | **No impact of diabetes on social engagements**  Dyad agreed that there was no diabetes related impact on social activities.  OVERLAP WITH QUALITY OF SUPPORT- BABYSITTER |
|  | **Psychological Distress** | | |  |
| **Dyad** | **Subthemes** | **Adolescent** | **Parent** | **Dyadic Code/Summary** |
| 4 | **Mental toll of Diabetes** | Reported no diabetes related affect on emotions in recent weeks. | Diabetes management reported as anxiety provoking but temporary (lines 825-831): *I do get anxious and tense, but it's a temporary thing, and once it's solved, it's like fine. But the diabetes does send me into an immediate anxiety thing in terms of response…It's like fight or flight.* | **Temporary threat response**  While the adolescent reported no perceived impact of diabetes on their emotions, the parent noted that the parental responsibility results in temporary fight or flight reactions until the issue is resolved. |
|  | **Diabetes visibility** | Reported no visible aspects to diabetes. | Reported no visible aspects to diabetes. | N/A |
|  | **Other** | N/A | Noted the area for improvement in terms of encouraging parental support. Noted parental management as a daunting and all-consuming task at the beginning and when you have gotten to grips with diabetes it would be beneficial to have connection and informed support (lines 877-880): *I found that hugely helpful. And I think though I was lucky that I happened to know someone, who knew someone. And I think for parents, when they're here as in-patients there's a real opportunity. They're not going to be ready for it, in the first two or three weeks.* | **Parental Support as invaluable**  Parent commented on the overwhelming nature of diabetes post diagnosis and the need to act on the gap in parental support groups. |

|  | **Theme** | | |  |
| --- | --- | --- | --- | --- |
|  | **Illness Representations of Diabetes** | | |  |
| **Dyad** | **Subthemes** | **Adolescent** | **Parent** | **Dyadic Code/Summary** |
| **5** | **Diabetes Awareness** | Good understanding of need for artificial insulin but no mention of auto immune condition (lines 12-14): *It's when a pancreas stops producing insulin. So you have to put in insulin by pens or by pump to keep your blood sugars steady. I’d have to keep an eye on the blood sugars.* A lack of awareness of potential complications of the illness (lines 28-29): *It's a little bit serious, but not very serious. Like, because once you have the insulin it's easy to get under control.* | Good understanding of need for artificial insulin but no mention of auto immune condition (line 11*): The inability to produce insulin.* Reported honest communication, and that adolescent understood why management was important and necessary and was able to have say in food choices (lines 43-46): *And it was explained to him that he could have whatever he wanted within reason. Once it was once he told us what he was eating. And once once it was covered.* Noted familial shielding of the child from the potentially fatal complications of diabetes (lines 68-75): *Potentially very, I mean we we try and I’d say hide the the potential seriousness from him like you know, I don't know how much he would be aware of that. But you know, you still hear of of people going into comas and not…So, I mean, we'd we'd be aware of the of the seriousness, but, you know, we try and not lay too much on him…* | **Diabetes shielding**  Across the dyad there was a similar explanation of the illness. With regard to complications or the seriousness of the illness the adolescent didn’t regard there to be much cause for control while the parent admitted to trying to not burden her child with the details of potential complications. |
|  | **Emotional Perceptions and Identity** | Acknowledged big shock at diagnosis but noted with adjustment living with diabetes became easier (lines 17-20): *It's a big shock when you're diagnosed first, but once you get on with it, it's not much like it's easy to keep track of… Once you know what you're doing.* Noted open disclosure as making him feel better (lines 39-40): *Tell people about it, like because you need to remember to tell people that you're diabetic?* Discussed emotional response to diabetes now with regards to knowledge acquisition (line 46): *I feel happy enough and confident that that I know what I'm doing.* | Reported lots of shock at diagnosis with no major symptoms, and no family history. Noted adolescent’s age at diagnosis as a positive (lines 27-28): *ehm there's never a good time to to hear, to get any news like that.* Discussed the post-diagnosis fear and the parental responsibility for management at the start (lines 102-103): *You know, we didn't leave them out of sight of one or the other of us, for I'd say the next year probably.* Noted more at ease with diabetes now since the diagnosis- gotten to grips with the illness (line 134): *More confident. I think the fact that he does take it all in his stride.* | **From Shock to acceptance**  Across the dyad both members noted the shock at the diagnosis but the slow adaptation to feeling confident in living with diabetes. |
|  | **Healthcare Orientation** | | |  |
| **Dyad** | **Subthemes** | **Adolescent** | **Parent** | **Dyadic Code/Summary** |
| 5 | **Attitudes towards healthcare** | Reported conscious of not eating too many sweets prior to diagnosis but noted the change since in food becoming more laboursome through the need for carb counting (lines 59-60): *you can't just walk up to the shop and get a big chocolate bar. You have to remember to bolus the chocolate bar.* Good availability of technology within the healthcare setting resulting in positive experience overall | Health-conscious family prior to the diagnosis so no effect on attitudes towards health. | **Health-conscious baseline with added effort for carb counting**  Both members of the dyad reported the family as health-conscious prior to the diagnosis but the adolescent noted there was still a change with regards to food become more effortful due to carb counting. |
|  | **Diabetes Management** | Reported technology as easier with less mental load and less of a physical burden to carry around supplies for (lines 92-98): *I really like the pump, like with the pumps that's what that's one less thing you have to carry. With the, without the pump beforehand I’d have to remember to bring the pen wipes and needles. But now we don't. You just need to remember to do a set change…Every few days.* | Carb counting as a focus of parental management but noted the increase in support groups and technology for this making it easier than years before (lines 161-165): *the carbs being aware of ehm counting the carbs and there's a lot of apps out there though, and ehm and support. That I think you know what? It makes things a lot easier than than they could be or or probably used to be.*  The mother noted the inconsistent use of metrics for “stable” control and how technology isn’t working as seamlessly as they had anticipated (lines 179-180): *And we just can't really get a handle on as he you know, he he'd he'd be stable for a short his HBA1C is okay, but again it's because it's the average.*  Reported the practical burden of carrying medical supplies and technology as being less awkward (lines 251-354): *And find a place because the matches would be in the evening, and he’d have to go off and find a place to do the Lantus. And and, you know, that was, that was awkward. So that I think we nearly was part of one of his main reasons for having to pump-he wouldn't have* *to worry about the injections and having to do things at particular times.* | **Practical burden of carrying medical supplies**  Both members of the dyad highlighted the benefit of technology in terms of being less awkward, offering more freedom with timings and practically being easier with less supplies required to be carried around. However, the mother also noted that she hasn’t found the technology to be as good as she hoped and sees in comparisons with peer support groups. |
|  | **Future expectations** | Noted he believes there is still a lot of public misconceptions about diabetes (lines 104-110): *I didn't really know much about it, but I think other people expect “Ohh he's diabetic, he must have eaten too much chocolate” ... “Or too much sweets. So his parents not must have, mustn't have looked after him…Growing up”.* Reported on a hope for more technological advancements in the future (line 113): *look positively towards it, like with more, bit bit more tech.* | Parental fear regarding the eventual transition from parental to self-management (lines 202-203): *And the thoughts of, the thoughts of that, the thoughts of to having to take the step back, that's that's scary.* | **Externally versus internally focused expectations**  There was differences reported with regards to the adolescent noting he thinks there will still be public misconceptions about the illness in the future while also acknowledging hope for more technological advancements. The mother however focused on her internal expectations for the emotional impact of transitioning responsibility for care over to her son. |
|  | **Information seeking** | Would information seek regarding technology and how they operate (lines 142-143): *Yeah, that was really helpful. So, like, you know what to do and how to install it. And like, what each thing does.* | Overwhelming amount of information at diagnosis led to gradual seeking information once they had gotten to grips with the basics (lines 229-233): *Even the basics is so much to take on, that it's pitched very well. That's kind of gradually more and more. Ehm and then, yeah, I think as he as he gets a bit more understanding of what's going on, I would have kind of read more of myself as well.* | **Getting to grips with diabetes**  Both members of the dyad noted information seeking once they got a few months past diagnosis. There was a pattern of requiring time to adjust to diabetes before seeking information about treatment (adolescent) or just in general (parent). |
|  | **Impact on the Vocational Environment** | | |  |
| **Dyad** | **Subthemes** | **Adolescent** | **Parent** | **Dyadic Code/Summary** |
| 5 | **Vocational Performance** | School support from a special needs assistant reported as very beneficial and noted he gets exceptions to school rules for management purposes (lines 67-73): *like I’ll be able to take out the phone and check the numbers and keep an eye and have the SnA's in the school.Which is very handy. And like if I’m low and don't see the notification, they can come up to me and tell me “Evan you're gone low”.* Noted getting on well with school but acknowledged the occasional fluctuations with BGs and the need for contingency planning within school (lines 155-158): *Like they'll be times where my bloods are say, like 20. So I’ll sorta walk around the car park.* Also highlighted peers openness to his disclosure (lines 179-180): *You know, I think if I tell people they’ll understand what it is or like understand, but I can just tell them what it is and explain it to them.* | Wanted to go back to a more full-on career role since the diagnosis but noted the parental responsibility and management hinder this (lines 275-280): *The thoughts of of going back when the phone is… Still, you know, I I suppose they take on more as they get older, but the phone is still…Kind of constantly…Going, it has to be yeah. Has to be, has to be monitored.* | **Feeling supported and feeling the need to support**  Differing views on impact of diabetes on vocational environments reported. Adolescent discussed positive support in school from both the academic team allowing him to be monitored and treat BG fluctuations as needed, and from understanding peers. Parent however, focused on her feeling unable to return to a more demanding job for the foreseeable future due to parental management and needing to be there for her son.  OVERLAP WITH VOCATIONAL VALUES |
|  | **Vocational Values** | Reported no change in school importance or goals since his diagnosis (line 176): *I still want to achieve what I wanted to do beforehand.* | Job not as important as it was pre-diagnosis noting that her priorities have re-shifted despite the growing independence an adolescent would normally have at his age (lines 282-283): *he's gone back to #1 focus. Whereas you know at that age, kind of… trying to take it trying to take a step back like you know?* | **Reprioritization of family over career**  While the adolescent noted no impact of diabetes on his school performance or goals the mother reported a conscious re-shifting of her job to a less demanding role so that her family’s health could take priority.  OVERLAP WITH VOCATIONAL PERFORMANCE |
|  | **Impact on the Home Environment** | | |  |
| **Dyad** | **Subthemes** | **Adolescent** | **Parent** | **Dyadic Code/Summary** |
| 5 | **Impact on Relationships** | Positive family relationships (lines 184-185): *Very good. Good relations with most of my family or all my family.* Reported an increase in family communication (lines 212-213): *I think that, yeah, it's gotten better. Like if I'm low, I can tell them and they know what's happening* | Reported close relationship with son. Noted she things the children get on well despite the diabetes (line 382): *I don't think she would hold anything against him…* Spousal relationship as coping relatively well with the diagnosis (lines 387-388): *there's probably changed topics of topics of conversation, but no, I think we've, I think we've coped with it fairly well.* | **Diabetes understanding and conversation**  Both members of the dyad highlighted good familial relationships and commented on the nature of communication change since diagnosis. The adolescent highlighted the understanding from his siblings now while the parent highlighted the overall addition in diabetes as a conversation topic. |
|  | **Quality of Support** | Noted siblings as having good understanding and taking an active role in management when parents aren’t around (lines 197-199): *if my mom or dad are gone out…My siblings will know how to do and what to do.* | Noted good level of support at home and everyone shifts roles as required. | **Good immediate family support**  Both commented on having support within the immediate family context. |
|  | **Impact on Family life** | Reported no impact on chores as he just waits till his BGs are back in range to continue (lines 202-203): *No, I still think but like if I'm low I can sit out for a few minutes and then go back in about 10-15 minutes.* Highlighted the benefit of the LTI scheme in Ireland and the easy access to medication as opposed to other countries around the world (lines 252-256): *I don't think there's much difficulty like in Ireland say you have the long term illness card…So you don't have to pay for most things. But like, it's different in the likes of America or something like that…* | No impact on household duties. Noted shopping has changed to encompass sugary drinks as medicinal requirements but LTI covers the medication in Ireland (lines 438-443): *like the Cokes and the juices and the …And the Lucozades. And I wouldn't have been, I wouldn't have been a great one for those beforehand…Whereas now, they have to be in the house.* | **LTI scheme**  Both members of the dyad commented on the LTI scheme as covering most of the financial aspect of diabetes care in Ireland. The mother noted the additional costs of specific foods for medicinal purposes while the adolescent commented on the global differences in diabetes care. |
|  | **Extended Family Relationships** | | |  |
| **Dyad** | **Subthemes** | **Adolescent** | **Parent** | **Dyadic Code/Summary** |
| **5** | **Extended family communication** | Reported no real impact of diabetes on communication levels but noted having to educate against misconceptions (lines 294-205): *Because, like, they say, “Can you be eating that?” and I can kind of explain “Yes as long as I take enough insulin for it”.* | No impact of diabetes rather an impact of the pandemic on communication and socialisation (lines 481-483): *Over the over the last couple of years, same as as there as there has been for everyone. But ehm what there has been no has been pretty much has been pretty much the same as pre, yeah…* | **Diabetes education**  While the mother noted no impact of diabetes on socialisation the adolescent noted the change in topics of conversation to include diabetes education. |
|  | **Extended family quality of support** | Noted no real support from wider family but noted again the support received from siblings instead (lines 283-286): *I could ask my brother, sister to like, say, get a needle. And they’d come back with the right thing.*  OVERLAP WITH QUALITY OF SUPPORT | Noted wider family as accommodating with food but no real emotional support available (lines 418-420): *They would, you know, if we were, if we were going over, would trying to accommodate, you know, does he need to to what? What's for dinner? What time it's going to be. You know, that kind of thing?* | **Immediate family as only support**  Both members of the dyad noted that the immediate family was their source of support. |
|  | **Social Environment** | | |  |
| **Dyad** | **Subthemes** | **Adolescent** | **Parent** | **Dyadic Code/Summary** |
| 5 | **Impact on interest levels & participation** | Reported persevering with his hobbies through his diagnosis (line 318): *Still doing it through the diabetes.* Noted peer and school support with management during activities has helped to not affect participation levels (lines 345-346): *So if even I go low, one of them will be able to go over, take out the lift juice, say “here drink that” and they’d know what to do and when to do it.* | Noted participation in all hobbies dipped immediately following the diagnosis while getting to grips with diabetes and trying to balance life (lines 507-511): *I’d say probably dipped initially…But has probably come back, has probably come back ehm since…Ehm you know the last year or so.* | **Divergent social engagement perspectives**  Differing views reported here with the mother noting the drop in participation in all her social activities following diagnosis. Contrastingly the adolescent highlighted still keeping up all of his hobbies through his illness thanks to good peer support. |
|  | **Psychological Distress** | | |  |
| **Dyad** | **Subthemes** | **Adolescent** | **Parent** | **Dyadic Code/Summary** |
| 5 | **Mental toll of Diabetes** | Noted confusion post-diagnosis and having to learn how to balance diabetes in his life (lines 351-358): *when I was diagnosed initially I was really worried. Because I I didn't know what was happening you know and I didn't know the future like…What the future would entail. And then like I was really confused and worried, but now I have a grip on it all and I'm not as confused and just I'm happier now.* | Reported an upcoming changeover in technology as playing on her mind (lines 552-554): *the thoughts of again something new and how long because the new one isn't out yet and… (pause) …Not knowing what way the, ehm, whole procedure will work…* Noted the burden of diabetes she carries as a parent worrying about not letting her child do things because of the risk and highlighting how he has already had family rule exemptions because of his illness (lines 605-606): *of course he had to get the phone in in, in primary school anyway ehm I feel he missed out, and is still.* Noted the parental balance of building his autonomy and being there to protect him (lines 634-636): *That I need to somehow loosen the reins and encourage him to go and at the same time, the terror of the thoughts of him being… because you know at that age they're fairly good at keeping an eye on it.* Noted how difficult it is mentally to have to say no to her child and highlighted trying to keep fairness between her children doesn’t always work-exclusion from school trips (lines 689-690): *It's very hard to say your brother and sister got to do this, but you can't.* Highlighted how there will be diabetes exclusions in her sons life and while they will try their best to navigate these as a parent she will bear the brunt of his emotional reaction to this as well (lines 711-713): *that's playing on the mind. The fact that with the best will in the world, there will be things that he won't be able to do or he won't be able to do at the same age or he won't be able to do it the same speed…* Finally the mother noted that she will have to help her child realise that he will face exclusions in life because of his diabetes (lines 740-742): *And at what point you kind of have to make him aware…of things like that, that's tough.* | **Adolescent acclimatisation and parental shielding**  The adolescent highlighted the initial emotional reaction to his diagnosis but commented on getting to grips with this new way of living. However, the Mother noted the difficulty in managing her own fear at the transition from parental to self-management and how that will play out as he may become distracted when with peers (line 657): *it becomes harder then to kind of encourage him to go.* She noted the degree of exclusions her son will face and has already faced because of his diabetes and having to be the one to shoulder that and alert him to the fact he maybe cannot do everything in life as his siblings or peers can. |
|  | **Diabetes visibility** | Noted the physical changes due to bodily absorption of insulin -being very skinny at time of diagnosis. | No diabetes related impact on appearance reported. | N/A |
|  | **Other** | N/A | N/A | N/A |

|  | **Theme** | | |  |
| --- | --- | --- | --- | --- |
|  | **Illness Representations of Diabetes** | | |  |
| **Dyad** | **Subthemes** | **Adolescent** | **Parent** | **Dyadic Code/Summary** |
| **6** | **Diabetes Awareness** | Reported giving a short answer for cause of diabetes (line 7): *when your pancreas doesn't produce insulin.* Reported never having thought about the cause of type one as she cannot remember her diagnosis (line 23): *ehm I haven’t thought about that either.* Reported on self-management as second nature (line46): *Check stuff… check your sugar, take your insulin and that’s it.* | Good understanding of diabetes including auto immune nature and need for artificial insulin (lines 15-17): *Ehm so type one diabetes is an autoimmune disorder ehm whereby the beta cells in the pancreas are no longer able to make insulin. So insulin needs to be replaced.* Also acknowledged the aspect of poor luck involved in the auto immune response triggering the onset of the illness (lines 29-31): *as I said, it's an autoimmune disorder. So ehm that means ehm that the body's own immune system makes antibodies ehm that attack some of the body's own cells. And in this case, it's the beta cells in the pancreas. So it's a, it's unlucky.* Reported on the never-ending management required to keep diabetes as a non-serious illness (lines 35-36): *It's completely serious. It's extremely serious. You have to do what you have to do. It's fine when it's been managed fine, but it's not fine if it isn't. It's day to day.* Acknowledgement of both sides out of range as dangerous for an individual with diabetes (lines 39-41): *well, the worst thing that can happen to anybody living with type one diabetes is not taking their insulin and or having a bad low. So we either, either or, so it's it's just a poor management which could lead to a potentially catastrophic outcome.* | **Adolescent Nonchalance versus parental management**  While the adolescent answered all questions shortly noting she doesn’t remember not having diabetes so find it a normal aspect of her life she doesn’t think about, her mother noted the importance diabetes management still holds. The mother gave detailed answers regarding potential complications and why management is so important for long term health juxtaposing to her daughter’s answers.  OVERLAP WITH EMOTIONAL PERCEPTIONS AND IDENTITY |
|  | **Emotional Perceptions and Identity** | Diagnosed very young so doesn’t remember not having diabetes so doesn’t report any real emotions attached with having the illness (lines 16-19): *I've, never really thought about it… to be honest…Because it’s just, I just kind of go with it like you know? I've always had it. So it's like…* Comparison to other illnesses for gauge of seriousness (lines 31-32): *It could be a lot worse. It's not that bad. Like it's not life changing.* Regarded diabetes as a normal aspect of her life at this stage (57-58): *ehm it doesn't really make like a massive impact like its just life, like I’ve always had it, so it's just there, you know.* Reported diabetes is in the background of her life (line 40): *Doesn’t really affect it that much. It's just kind of there… in the background.* | Reminiscing on the difficult shock diagnosis and how it feels like a different life now that they are so far into the diagnosis (lines 20-26): *I suppose it's so long ago now she was two and a half and she was diagnosed, as I said, and she's 15 now. So it seems like a lifetime ago in many, many ways. So…But ehm so we've lived with it all that time while her growing up, but yeah it was an enormous shock at the start… it was shocking. And ehm it was it was extremely difficult.* Reported on the significant amount of time required for diabetes to be integrated into everyday life and the importance it holds (lines 46-49): *now it, it, you know, it's we're doing it for so long that it is part of day-to-day life and it doesn't stand out all that much. Ehm but it definitely takes quite a bit of time to get to that point. Ehm it's not front and center anymore, but it's as important as the most important thing that any of us would have to do on any given day.* Noted the emotion she still carries over the diagnosis 13 years on (lines 66-70): *I feel regretful about it. I hate that she has it still, obviously…(pause)…(sniffing) Ehm but it's fine, you know it, it has never stopped her doing anything, and it won't ever stop her doing anything. But I hate that she has it.*  OVERLAP WITH FUTURE EXPECTATIONS | **Integration of diabetes into daily life**  Both members of the dyad discussed the great length of time since diagnosis and how diabetes is just another aspect of the daily routine now. The mother however noted the raw emotion she still carries over the diagnosis and noting the huge shock at the time highlighting some maybe unprocessed emotions due to the need to deal with daily management (line 137): *You just deal with it. You don't talk about how you feel about*. |
|  | **Healthcare Orientation** | | |  |
| **Dyad** | **Subthemes** | **Adolescent** | **Parent** | **Dyadic Code/Summary** |
| 6 | **Attitudes towards healthcare** | Believes she is more health-conscious than her peers due to her diabetes particularly regarding food (line 78): *I’d need to think about it more. Like you need to think about your eating more.* Good experiences with healthcare staff (line 99): *Like they're all pretty nice.* | Didn’t change general attitude towards health (line 90): *I understood that ehm that Fiona was still healthy.* Noted mixed experiences with the healthcare team over the years but acknowledging that all are on the same team at the end of the day (lines 100-103): *It's like your child going through school or anything. Every day is not going to be a walk in the park. We've hit, hit, you know, bumps along the road very definitely. Ehm but I 100% understand that we're all on the same team and we're we're fighting the same battle.* | **Mixed experiences with healthcare teams**  While the adolescent reported positive experiences with the healthcare staff the mother acknowledged the few run-in’s they have had over the years while noting the overall fight for best quality of life for her daughter. |
|  | **Diabetes Management** | Reported diabetes management as so integrated and second nature in her daily life that she struggles to explain what goes into it (lines 85-86): *it’s like I don’t know (laugh). Like I do know, but like I don't know to explain it.* Reported no major feelings towards technology (line 95): *the pump like it does the job.* | Noted the age at diagnosis playing a role in how much parental management is required but acknowledging it will always be 24/7 never-ending management at first (lines 81-86): *it's 24/7 care at the start and for for quite a long time after that it didn't, when it it dies down, but then the age of the child probably adds to it. Although I would say if somebody a parent of a teenager that was just diagnosed it’s still 24/7. But you know, I suppose a younger child is so dependent and you're doing everything for them anyway. Ehm so yeah, it was, it was round the clock ehm thinking about minding her diabetes and minding her with her diabetes, when she was diagnosed.* | **Second Nature versus Never-Ending management**  Differing perspectives reported here with the adolescent noting that diabetes is just part of her day and she cannot separate it from the rest of her routines. However, the mother noted that parental management is always there in the background consistently regardless of the child’s age. |
|  | **Future expectations** | Noted diabetes as separate to all of her future plans or not impeding on any plans she has (lines 107-109): *I've got my whole life plan, you are asking the right person this, so like it has nothing to do with diabetes though? Let's see, how I can bring diabetes into it. Oh… I don't know. Like keep it going well and it'll be grand.* | Noted her parental fear at child taking full responsibility for the diabetes and the burden this will be in life (lines 117-120): *She'll always be able to do whatever she wants to do with it. But it's it's always, you know it's an extra job to do and I worry about, you know, when I'm not there to mind her somewhat (tearful). Why am I crying? (laugh) It's still. It's amazing how emotional you still get, you know?*  OVERLAP WITH EMOTIONAL PERCEPTIONS AND IDENTITY | **The burden or lack thereof of diabetes**  The adolescent reported diabetes as having no influence on her future plans. The mother however noted the added burden her daughter will always carry with her because of the diabetes and the worry associated with that emphasizing a juxtaposition in future expectations. |
|  | **Information seeking** | Information seeking through parent’s medical profession and noting due to duration of illness not having much need anymore (lines 115-117): *No, because my mom's, my mum's like Diabetes Nurse like a specialist in it. So like anything I needed, I'd ask her and like from having it so young, I kind of grew up and I just got everything as I went…* With regards to treatment information seeking reported a nonchalant approach with relying on maternal and staff opinions (line 131): *My mom decided she wanted that one and I was like, “G”.* | Noted not a huge amount of need for information seeking due to her job and recent experience of diagnosing a child in A&E led her to self-diagnosis her daughter (lines 188-192): *because I was already ehm, a, a pediatric nurse, not a diabetes nurse at that time. I ehm, I had a certain amount of knowledge, and I worked in ED at the time…And I had only recently looked after a little boy who was newly diagnosed.* Noted her medical background allowed her the insight to research what she needed to know but not overwhelm herself (lines 194-195): *I looked a little bit, but I also knew not to look too much type thing and just to kind of stay with.* | **Mother’s medical knowledge**  Both members of the dyad commented on the mother’s role as a diabetes nurse (or paediatric nurse initially) as making information seeking very easy. This was highlighted by the lack of opinion the adolescent now reports on her treatment regime as she trusts her mother’s opinions above her own research. |
|  | **Impact on the Vocational Environment** | | |  |
| **Dyad** | **Subthemes** | **Adolescent** | **Parent** | **Dyadic Code/Summary** |
| 6 | **Vocational Performance** | No affect of diabetes on schoolwork (lines 144-145): *No (laugh). I wish it did, but no (laugh). That way I could get away from class, but no.* Noted trouble with teachers not realising the severity of the illness or undermining her health leading for her to have to self-advocate (lines 185-189): “*Why were you inside for so long blah, blah, blah?” And I was like “My sugar is low. I told you that. I told you had to sit inside for awhile” and she was like, “Yeah, well you shouldn't be allowed to sit inside for like, 15 minutes” like and so I was like “Well, you can either pick me up off the floor when I collapse, or you can let me sit inside”. They are the options. It's not really an in between so*. Reported that diabetes is in the background of her life and so her peers wouldn’t really bring it up (lines 204-205): *but like once my friends know they're like, leave it, they won't keep banging on about it.* | Adapted work schedule in order to have spousal tag team with one partner always at home (line 226): *it changed how I did my job. It changed the hours I worked.* Had this altered night shifts for years for not wanting to bring someone else into the responsibility or managing the diabetes (lines 235-236): *And we continued that for several years because it was definitely the better option than bringing in a third party to help mind her- That's what we were comfortable with.* | **Diabetes adaptations and growths**  The adolescent commented on diabetes not having an effect on her school performance for the most part but acknowledged that some teachers have had poor understanding leading her to have to stand up for herself. The mother acknowledged how diabetes adapted her job in terms of moving to night shifts in order to have full time parental management. |
|  | **Vocational Values** | Reported not really liking school (line 163): *it's not that like big of a deal for me.* However also noted her ambition and not letting diabetes affect any of her academic or vocational goals (line 170): *No. If I want it, I’ll do it.* | Reported job as equally important to her now as it provides an outlet outside the home and also allowed for the parental childcare, they were comfortable with (lines 268-270): *it's actually a nice mix of…Being around for them and and going out to work.* Noted diabetes as not stopping work goals but prolonging them (lines 283-284): *I would say it, it hasn't been a major barrier to anything that I've really wanted to do.* | **Diabetes as not holding them back**  Both members of the dyad noted that diabetes wouldn’t hold them back in life. The mother noted while some work goals had to be adapted she has reached them since so they were more so prolonged then stopped. |
|  | **Impact on the Home Environment** | | |  |
| **Dyad** | **Subthemes** | **Adolescent** | **Parent** | **Dyadic Code/Summary** |
| 6 | **Impact on Relationships** | While initially using humour to deflect from if diabetes had any impact on her relationship with her mam she then acknowledged she believes them to be closer because of it (line 224): *Probably made it closer.* Reported to be closer with her mam than her dad due to the time-consuming nature of diabetes and time spent together at appointments etc. Noted siblings treat her the same as if she didn’t have diabetes (line 235): *Tom (brother) and Rose (sister), like they don’t care. Like it doesn’t change.* | Reported good relationship with her daughter (lines 301-302): *We're very close. Ehm yeah, she's great fun. We're, we're, we're good buddies.* But became emotional on thinking about whether their relationship is any different because of diabetes (lines 310-311): *I guess I have, I have probably just minded her (tearful).* Overall noted good supportive family relationships with other children and her husband as well. Noted the topics of conversation evolved to include diabetes (line 488): *I suppose it's it's shifted the focus of the communication quite a lot…* | **Closer bond due to diabetes**  Both members of the dyad highlighted respectively that they believed they had a close bond due to the level of interaction they have with each other that wouldn’t have been the same without the diagnosis. |
|  | **Quality of Support** | Noted her independence and tendency to do things herself while admitting that all of her immediate family would offer her support (lines 255-258): *Yeah, they probably all would, but I just if I wanted to do it myself, I would do it. If I was low and all…Like I'd do it. But if I don't want to do it I’m like “I can't do it. Sorry” (laugh).* | Immediate family as helpful and husband as great support in balancing the children’s needs. Noted wider family as unhelpful with the diabetes aspect (lines 361-362): *Yeah. I know I, I certainly, I, I certainly needed help. But there wasn't really anybody that could do very, very much.* Noted the parental management and not wanting to burden others as potentially affecting external support (lines 377-379): *But at the same time, it was the there was that sense, you know rightly or wrongly, and I don't think it's a great idea, but there was that sense that this is too much for anybody else to take on. We just need to do this.*  OVERLAP WITH EXTENDED FAMILY COMMUNICATION AND SUPPORT | **Pushing through personal needs**  Both members of the dyad highlighted how they have tendencies to push through difficulties. The adolescent noted that even when low if she wants to finish a task she wont ask for help. The mother noted feeling like she couldn’t burden the wider family with diabetes so pushed through needing practical and emotional support with her husband. |
|  | **Impact on Family life** | Admitted to using her diabetes as an excuse to get out of chores at times (line 241): *Ohh, I just say it does but it doesn’t, like “Sorry I can't do it. I'm low”.* Queried healthcare costs in Ireland showing lack of awareness of finances and diabetes (line 281): *isn't like healthcare in Ireland like free?* | Children’s needs as taking priority over all else till a balance with life and diabetes was found (lines 343-344): C*hores around the house, they came secondary, I guess, to what the kids needed…*Noted the benefits to the healthcare system in Ireland (lines 406-408): *we've both always worked and ehm …you know we do live in a country where all the, you know, the pharmacy bits are provided. And we're very, very lucky for that.* | **Prioritising children and using diabetes to her advantage**  The adolescent noted that at times she will pretend to be low in order to get out of chores she doesn’t like doing. The mother however, showed how she had prioritised the needs of her family above all else at the time of diagnosis. |
|  | **Extended Family Relationships** | | |  |
| **Dyad** | **Subthemes** | **Adolescent** | **Parent** | **Dyadic Code/Summary** |
| **6** | **Extended family communication** | No diabetes related impact on communication or family interest (line 301): *No, I think I'm just lazy .* | Noted a significant change in communication at the time of diagnosis with the wider family feeling like an obligation at times (lines 424-425): *I wasn't interested in that and, but yeah, there was some things you had to do, but I didn't want to do them.*  Noted parental management as superseding family engagements after diagnosis (450-452): *It probably did change around the time of diagnosis because ehm yeah, I I suppose I didn't want to leave Fiona. So it was, ehm yeah, I would, I would kind of decline ehm to do things without, without her or I just try to bring her along with me.*  OVERLAP WITH QUALITY SUPPORT | **Drastic family reprioritisation after diagnosis**  While the adolescent doesn’t remember any impact on wider family contact the mother recalled the dramatic reduction in her interest levels after diagnosis. She noted the fear at leaving her child and burdening others stopped her family engagement. |
|  | **Extended family quality of support** | Noted she believes her wider family would be willing to help her if she asked for diabetes support but noted the lack of awareness and understanding as barriers to this (lines 309-310): *Yeah, they probably wouldn't know anything, though they kind of be like “What do I do with you?”* | Noted good practical support for the other children and emotional support from one member in particular (line 431): M*y mom would have been a good support kind of emotionally.* Noted no support for daughters’ diabetes over the years and highlighted still a lack of understanding at this time (lines 467-468): T*hey would have an, they would have an idea about it, but not ehm they, they wouldn't know, they wouldn't really understand it.* | **Lack of true understanding**  Both members of the dyad noted that there is a lack of understanding about diabetes within the wider family members to this day which has reduced the amount of support which could be requested. |
|  | **Social Environment** | | |  |
| **Dyad** | **Subthemes** | **Adolescent** | **Parent** | **Dyadic Code/Summary** |
| 6 | **Impact on interest levels & participation** | No effect of diabetes on social activities. | Reported prior to the diagnosis she had withdrew from her hobbies due to having three young kids and so she had chosen to surround her life around her children and their needs (line 512): *That was completely my choice.*  Noted family activities took a small pause while the family adjusted and learned to balance diabetes and life (lines 532-533): *I would say I took a little bit of our, of a hiatus for about a year or so…*Noted spousal time became non-existent post diagnosis but due to choice- not wanting to leave their daughter (557-558): *Well, what totally weaned was myself and Kevin (husband) going out together.* | **Choosing to prioritise family**  The mother noted her and her husband’s choice to withdraw from activities around the time of diagnosis for not wanting to leave their daughter and also needing to adjust to life with diabetes.  OVERLAP WITH EXTENDED FAMILY COMMUNICATION |
|  | **Psychological Distress** | | |  |
| **Dyad** | **Subthemes** | **Adolescent** | **Parent** | **Dyadic Code/Summary** |
| 6 | **Mental toll of Diabetes** | Overall reported little to no affect of diabetes on her emotional states but noted some temporary worry associated with hypos (lines 394-396): *Not really only worry I really have is that I'd go low or something…But even that's not like, you can think, it's an easy fix.* | Noted an ever-present unwarranted guilt surrounding the diagnosis and the fact her daughter lives with diabetes (lines 596-597): *Ehm probably around diagnosis, yes, there is that sense of of self-blame with it, which is you know, which is unfounded.* | **Temporary versus ongoing emotional toll of diabetes**  There were differing perspectives reported with the adolescent noting fleeting hypo anxiety and the mother recounting the ongoing guilt over her daughter’s diagnosis that despite having the medical background to know she couldn’t have prevented, is always with her. |
|  | **Diabetes visibility** | No reported impact of diabetes on physical appearance. | Noted the visible strain and tiredness around diagnosis (lines 623-627): *In the early stages, yes, you you could see the strain and and…And you know the worry but at this stage, I've, I've I'm hardened to it to an extent. So no, I don't feel that has a massive impact now.* | **Visible impact of diagnosis**  The mother noted the visible strain and toll of diabetes on herself physically around the time of diagnosis but acknowledged no real impact anymore. |
|  | **Other** | N/A | Highlighted that emotion can be viewed by staff as weakness or not coping when the families have no choice but to cope with diabetes (lines 156-163): *I do think that sometimes when parents show any emotion and I see this in both places…That you know they're, they're not coping or they haven’t accepted it and neither of those things are true, because as you know, they are coping…they are coping extremely well and they are managing extremely well. But they're not, they wish they didn't have to and that's it.* | **Parental Coping**  Noted from her experience as a diabetes nurse that parental emotion can be regarded as a sign of not coping when she highlighted it is the opposite. It is the expression of pent-up emotions that parents are forced to work through with the management every day. |

|  | **Theme** | | |  |
| --- | --- | --- | --- | --- |
|  | **Illness Representations of Diabetes** | | |  |
| **Dyad** | **Subthemes** | **Adolescent** | **Parent** | **Dyadic Code/Summary** |
| **7** | **Diabetes Awareness** | Good understanding of need for artificial insulin but no mention of auto immune condition (lines 10-12): *It's when you're a pancreas stops working, stops producing insulin. So you have to like every time you eat, it needs it so you have to like, give it yourself, either by injections or a pump.* Remembered awareness that the physical symptomatology at the time of diagnosis she was experiencing were wrong (lines 23-26): *I remember doing PE and I had like no energy and like I went home and told my mom and cause like, I'm really active. So it's kinda weird that like I had no energy. And also like, I kept waking up during the night to go to the bathroom. Like I just kept needing to go and I was thirsty. So I knew that's something like was wrong.* Awareness now of treating diabetes as a serious health condition Lines 34-36): *like obviously when you're low, I think it's dangerous. Like you need to like take action and stuff like. If you don't take action, you can get like very serious like you can like pass out and stuff.* Highlighted need to stay alert to diabetes and not being able to forget about it (lines 38-39): *I think like with your friends and stuff sometimes ehm you just need to like, be wary of it. Like, don't get too distracted, like, always kind of think of it.* | Good understanding of diabetes and auto immune nature from medical profession (lines 20-22): *Well, it's when your ehm immune system attacks the beta cells in your pancreas and eventually kills them off so that you can't produce insulin, which you require, to use ehm the sugar in your blood.* Noted that a nephew has diabetes so there was some familial understanding of the illness at diagnosis and working as a doctor she knew something was wrong. Awareness of diabetes as requiring a lot of management (lines 54-55): *Whereas I suppose I just said okay, we're just gonna have to get on with this, you know, I knew it was chronic disease and it is tough going definitely.* Reported on the unlucky nature of her daughter getting diabetes when while there is a genetic predisposition it has to be triggered and none of the siblings got it as well (lines 90-92): *Well, she would have had the …underlying… (pause) … genetic predisposition for it, and then it could have been any number of things, like a virus, which might have triggered the immune system to start attacking the beta cells*. Good supportive experiences with healthcare staff leading to comfort with treatment and not wanting to progress (lines 74-77): *The specialist nurses in Crumlin were so good at giving education and support because we got very comfortable with the multiple daily injections that really we didn't want to change because things were working well.* Noting the potentially fatal consequences if diabetes was poorly managed (lines 111-112): *The worst… is… I suppose, a DKA and dying from it. You know, if she wasn't picked up ehm not getting into hospital quick enough.*  OVERLAP WITH ATTITUDES TOWARDS HEALTHCARE | **Medical Knowledge highlighting serious nature**  Due to the mother’s medical profession both members of the dyad providing a good understanding of diabetes, their individual bodily awareness something was wrong and awareness of the potential complications of the illness. The medical background appears to have led to a congruence across the dyad of the importance of management. |
|  | **Emotional Perceptions and Identity** | Fear at diagnosis as finding diabetes even with some familial awareness as a daunting prospect to live with. Noted pity in the family but a gradual getting to grips with diabetes (lines 15-21): *I think at this start it's like a lot harder, like when I first like, the sound of it sounds a lot like scarier than it actually is. So when I first like heard it, I was like really scared and cause my cousin had it like everyone was kind of like, like always felt bad for him and I was always got kind of scared when he, like, took out his pump, or anything like that. But like, when you, like, get to know it. And like when you have it yourself, I think it's a lot easier to understand.* Noted craving normalcy while appreciating check ins from those around her (lines 43-46): *Like, it's not like that big of a deal like you don't need to be like, people like always checking up on you, like obviously it's nice if people like check up on you, but like, you don't wanna constant person like at you all the time. You just wanna be like normal kind of.* Awareness of period to get to grips with diabetes and her progression to treating diabetes like second nature or an automatic process (lines 50-51): *I kind of just do it automatically like obviously at the start it's a lot different, but when you’ve had it for a while you don't even notice it sometimes.* | Lack of parental self-care during diagnosis leading to personal injury while also noting the emotional difference between working as a doctor with patients versus her own child (lines 54-70): *Eventually the Reg came and was looking after her and I said “Okay, I'm gonna just step out now” because I knew she was all sorted and then I collapsed on the floor. Yeah, yeah. Whacked my head (laugh). So obviously it did, you know, hit me hard…And you know ehm it was just, I suppose, the emotion of having it like it's very different ehm I suppose talking about diseases and and, you know patients are more, ehm…It's more of an objective thing. You know, the way you can watch surgeries or anything going on, but when it's your own child and ehm it is very different.* Reported on the emotional impact on her daughter particularly in school with peer stigma and lack of understanding from teachers (lines 137-141): *The multiple daily injections were tricky because some of, say, the children in her class were a little bit squeamish. So she felt a bit uncomfortable injecting in front of them.* Noted trying to get daughter onto clinical trials and the frustration at lack of research in Ireland (lines 164-170): *Yeah, I suppose, like I'm always thinking of what's coming down the line. And I did try to get her on a clinical trial in ehm San Francisco…When she was first diagnosed. And, you know, it had to be within the 1st 100 day window. And my brother lives in San Francisco, and it was Teplizumab (drug) ehm was the drug. And because we weren't US nationals or Belgian, which was the drug company, they wouldn't take us…* Noted worrying about daughter’s future particularly regarding pregnancy and the complications that diabetes will add to that one day (lines 195-198): *But I suppose the next thing I'm thinking about for Grace is you know pregnancy that sort of thing…You know, it's hard enough.*  OVERLAP WITH FUTURE EXPECTATIONS AND INFORMATION SEEKING | **Medical knowledge not easing the emotional burden**  There were differing perspectives across the dyad reported here with the adolescent discussing a difficult adjustment post diagnosis but an automatic approach to diabetes management and diabetes integration within her life now. However, the mother reported on the emotional burden and the worry that she still carries to this day perhaps heightened from her medical knowledge of complications as well as simply seeing her daughter ill rather than a patient. |
|  | **Healthcare Orientation** | | |  |
| **Dyad** | **Subthemes** | **Adolescent** | **Parent** | **Dyadic Code/Summary** |
| 7 | **Attitudes towards healthcare** | Positive increase in health-consciousness since diagnosis emphasizing food intake (lines 61-63): *It's good to know like you like, you're more conscious of, like what you're eating and stuff like that and like, taking care of yourself, like feeling yourself and stuff like that.* Reported on familiarity with healthcare staff from cousins’ diagnosis easing the fear at diagnosis and providing a positive healthcare experience (lines 77-79): *Like I was really well looked after, like when I first went in, I remember like, they were really nice. And because I was really scared when I first went in.* | Great experience with the healthcare staff team (lines 215-217): *I think did we stay there three nights and you know we're very comfortable with, the education was super, the nurses were just lovely. And so that all went great.*  OVERLAP WITH DIABETES AWARENESS | **Positive healthcare staff experiences easing emotional burden**  Both members of the dyad noted good experiences with the staff team and highlighted easing fear and creating a comfort within treatment regimens that they didn’t wish to change. |
|  | **Diabetes Management** | Noted the need for frequent to constant bodily awareness, monitoring and management (lines 71-73): *I mean kind of just making sure like everything's on track. Like you need to, just like I'm making sure that you're feeling good and stuff like that. Like I’m always like checking up and like making sure your blood sugar is good and stuff like that.* Highlighted the awkwardness of carrying medical supplies for the injections making the pump a much more user-friendly method of treatment (lines 90-91): *Like ehm like for carrying around like pens and stuff it is a bit tricky. So I think like for day to day it's a lot easier using the pump.* | Parental to self-management as difficult to let go control of but noting child as very aware and conscious of keeping within the desired range (lines 229-231): *Overtime I had to kind of just hand that over to Grace and give her more autonomy on that and she's great. She loves her sweets and all that, but she she would be good at keeping an eye on it at the same time because she is 40 (Hba1c).* Reported on child understanding as helping to maintain the diabetes management due to awareness that diabetes can fluctuate easily and be a rollercoaster to restabilize (lines 236-237): *Take too much insulin than you're going low and you know, it does wobble it all around, whereas when she's nice and steady and her food is sensible, it's it's much better.* Current treatment plan as operating okay but still room for more seamless treatment plans and much more options available in the US (lines 286-287): *Now it is helpful that it's it communicates, and it does the overnight, but there's definitely room for improvement.* | **Hypervigilant management**  Across the dyad both members highlighted the need for 24/7 constant diabetes management as well as the potential complications if this isn’t engaged in. The adolescent noted that the pump provides some ease to her day while the mother noted there is still room for improvement with the technology highlighting the different options available in the US. |
|  | **Future expectations** | Noted no major expectations for technological advancements but hope in information seeking around an eventual cure (lines 96-103): *I think just like kind of the same like cause I’m the pump, I don't think a lot more will change, but there's obviously new like technology coming. So I think that it's good to look forward to like what the new technology will do and stuff like that. Like my mum, I remember she was looking at like clinical trials and like…I've heard a lot of things that like, that some people have, like recovered from it, so and there's like watches that can like, check your blood sugars for you or so I think it's exciting to see like what new things will be in the future.* | Discussed expecting more medical advances within the UK and hoping Ireland would eventually be included within that network as well (lines 188-190): *And then you never know in years to come with the stem cell treatments and all that. You know, even you know, with the audit of get the registry.* Noted the potential and expected difficulties with her daughter’s hopeful future pregnancies (lines 195-198): *But I suppose the next thing I'm thinking about for Grace is you know pregnancy that sort of thing…You know, it's hard enough.*  OVERLAP WITH EMOTIONAL PERCEPTIONS AND IDENTITY, AND INFORMATION SEEKING | **Hope for an eventual cure**  Both members of the dyad referenced a clinical trial and hope for future stem cell research to cure diabetes. The mother however noted with her medical knowledge she also expects to see more basic improvements in nationwide care services such as database inception and access to technology. |
|  | **Information seeking** | Noted technology as the gold standard of care and having an interest in researching possible technologies or new treatments for herself (lines 109-114): *I think when I first got diagnosed, I was like really interested and like looking for different sensors and stuff because obviously when I was like just on the pump and didn't have a sensor, I was a lot, a lot more like inclined to like look up different things. But now that I'm, like, happy on my pump and my sensor, I'm less inclined to like look up things. But often, like sometimes I like look up like videos and stuff and see what other like sensors and pumps are out.* Noted trusting the healthcare team mostly for opinions on treatment but likely to double check for herself (lines 121-123: *Yeah, like I I trust what people like, what the nurses say. But like, sometimes I want to look up more things to see. Like what would suit me, because, like, obviously different people suit different things.* | Information seeking as normal aspect of her diabetes routine to stay up to date in her field and personally. Noted she switched her academic research to focus on diabetes and would be researching the next treatment options (lines 320-323): *Yeah, no, I would be searching for more information. Like, I mean trying to get her on the clinical trial…Was totally my ehm doing.*  OVERLAP WITH EMOTIONAL PERCEPTIONS AND IDENTITY, AND FUTURE EXPECTATIONS | **Knowledge as key to management**  Across the dyad both members noted information seeking as a regular task they engage in. It was reported in terms of current treatment options and the more future expectations side as well. The adolescent noted that this was important in order to feel sure she was getting the best treatment that suited her lifestyle. |
|  | **Impact on the Vocational Environment** | | |  |
| **Dyad** | **Subthemes** | **Adolescent** | **Parent** | **Dyadic Code/Summary** |
| 7 | **Vocational Performance** | BG fluctuations after food as leading to concentration issues (lines 131-132): *The only thing that would affect me is like, if I'm after like lunch and I'm like a bit high ehm high blood sugar like I get like I find it harder to focus.* Peer disclosure as a positive experience as she gained support (lines 157-158): *I think that they managed really well and I just like told them about it and yeah, they can help me if like anything’s wrong so that's nice.* | Changing job deadlines and focus of research due to the diagnosis while trying to get to grips with diabetes (lines 345-348): *I turned them down because, you know, it was just too much hassle, you know…Having to, you know, juggle everything.* Noted less of an impact on work now as child has matured (lines 386-389): *As Grace has got older ehm she has matured an awful lot in the last kind of year…Or so. Yeah, I'd be very confident in her ability to manage.* Noted co-workers at the time as very understanding given medical background (lines 400-402): *They knew, you know, this is, this is a big deal. Nobody wants their kid to have type one diabetes all of a sudden. Do you know what I mean? Especially a child.* Boss as aware of familial impact (lines 404-407): *Actually my supervisor on the research project said to me, you know, this is going to take four or five years for you to feel okay…And that turned out to be true.* | **Vocational support**  Both members of the dyad commented on support systems. The adolescent noted the comfort she receives from peers knowing she has their practical support should she need it while the mother discussed the colleague understanding of the adjustment required for diabetes and the difficulty seeing your own child ill. |
|  | **Vocational Values** | School as becoming more important with age rather than any affect of diabetes on this value (lines 146-148): *It's just like I got diagnosed when I was in Junior School, so I didn't really take school that serious in Junior School, but now I take a lot more serious, so I think it's gotten more important.* Reported no affect of diabetes on academic goals. | Had to change goals within her career to allow for time to balance life and diabetes (lines 368-369): *But with Grace ehm being diagnosed in the February, I just had to drop all those plans.* Considering a step backwards in career for ease of availability for her daughter (lines 372-375): *I've definitely taken a step back and I was even thinking there I, when we were meeting with the tax people, I used to work with Ernst and Young that maybe that's an easier job for me to do, that I would be around. I could work from home and ehm I'm just more available, you know.* Noted job isn’t as important as it was prior to the diagnosis. | **Child as more important than career**  The mother noted she had made significant career changes since the diagnosis and was considering another backward step in order to be more present and available for her daughters needs. The adolescent on the other hand noted on impact of her diagnosis on school goals. |
|  | **Impact on the Home Environment** | | |  |
| **Dyad** | **Subthemes** | **Adolescent** | **Parent** | **Dyadic Code/Summary** |
| 7 | **Impact on Relationships** | Really close relationship with mother with good communication that was aided by the mother’s medical knowledge (lines 162-164): *She was really helpful because she's like a doctor, I think that it really helps and she's always, like, checking up and I'll tell her if things go wrong. So that's really good.* Overall close family relationships and noted having to calm her sister’s anxiety around needles post-diagnosis (lines 177-180): *So at the start, she was, like, more scared than I was. Like I don't really mind, like anything like needles or anything but my sister was really scared, so I had to, like, tell her to… (pause) … like relax and stuff (laugh).* | Parental management and monitoring as always there prior to diagnosis so reported no impact of diabetes on dyadic relationship (lines 426-428): *I don't think this there is much of a change in our relationship. I could always be keeping an eye on her. Always ask her “Is everything okay?”, you know?* Noted the shifts in responsibility taken by other children as well post-diagnosis (line 441): *Like her older sister, probably would have taken on a lot of responsibility.* Increased communication within the home when would usually decrease at that teenage stage (lines 472-473): *Probably increase like you need the communication channels open all the time, so you do need to, you know make sure she's okay.* | **Close bond and consistent parental monitoring**  Across the dyad the mother’s checking-in was noted as a positive aspect of their relationship. |
|  | **Quality of Support** | Good support from all family members if she required it (lines 203-204): *Yeah. I think, Maeve (sister), like my sister, brother, Mom or Dad would ehm come in and help if I needed it.* | Reported good support from all family members and increased support from husband year of diagnosis (lines 460-461): *Like the hardest time was definitely when she was in 6th class and my husband was great at helping out.* Increased support from elder daughter as well (lines 444-446): *And if I'm away, she probably is the one, you know, who's helping me, you know? And she does give me a lot of help to make sure everything is okay.* | **Supportive family**  While the adolescent noted she could ask any member of her immediate family for support the mother highlighted her husband the year of the diagnosis and now her eldest daughter as taking on more household responsibility when she is away. |
|  | **Impact on Family life** | Trying not to let diabetes affect her jobs around the house while echoing her awareness of minding herself (lines 188-191): *Not really like obviously if like I’m low or something like well obviously I’ll like take my time and I take it easy, but I like makeup for that, like I don't really like… (pause)… like obviously, sometimes I like take it easy (cough in background) and I cannot do stuff, but I'll try to like do everything I can.* Reported no impact on finances due to LTI scheme (lines 215-216): *No, there wasn't cause you get most of like it from like healthcare like. It's all kind of given to you by the government.* | The pandemic coincided with adjusting to the pump making it less of a stressful time than anticipated. Noted needed lots of support from her husband at the time of diagnosis (lines 461-464): *I suppose with the pandemic, you know, we were all at home, so you know, so we kind of those years kind of passed. And now we're, she's so much older and well established that it's great. Like even the pandemic was so handy for her starting the pump, you know?* Noted LTI scheme in Ireland as great when compared to the US (lines 482-483): *I mean, we're so lucky getting everything for free compared to the states like.* | **No major familial impacts**  Both members of the dyad noted that while diabetes is always present in terms of monitoring and management needs, that it is just a part of everyday life and has been less impactful than the mother would’ve expected. |
|  | **Extended Family Relationships** | | |  |
| **Dyad** | **Subthemes** | **Adolescent** | **Parent** | **Dyadic Code/Summary** |
| **7** | **Extended family communication** | Good communication and engagement with the wider family noting a good familial understanding and educating family when they ask about diabetes (lines 223-228): *My granny always like asks and checks up on me, which is nice ehm… Like my cousin, a girl in her class has it...And so she was, like, interested in it. So I tell, like, them about it. And like, everyone kind of knows, I have it so if anyone was, like, interested in asking or I’d just tell them about it.* | No real affect on communication though acknowledged an initial period of diabetes always on the mind (lines 528-529): *Ehm maybe in the beginning we curtailed going out or, you know would would have been on my mind.* Noted initially post diagnosis there was parental frustration and concern about attending family events (lines 537-543): *I suppose in the beginning you have a point. I, you know, if there was a ehm parties where there was a load of sweets on display, I was a bit kind of you know, we don't really need this…Or my sister would be inclined to go “oh have a sliced cake, have a sliced cake” like like Mrs Doyle (laugh) shoving it on people. So that was a bit frustrating in the start…* | **Initial hesitation despite relative understanding**  While the adolescent noted a good level of support and awareness from her wider family now, the mother acknowledged the initial period post-diagnosis as being distracted by diabetes and siblings pushing her to loosen up about food management. |
|  | **Extended family quality of support** | Reported believes would have support if she asked for it. | Noted didn’t need help with the diabetes but knew it was there if required (lines 516-517): *I didn't need to like reach out for support.* Yet noted how she believed them to be lucky as her medical background gave her knowledge that her sister and nephew didn’t have (lines 512-514): *They were kind of in the dark and it was stressful for them. And even then, as he got older, so he was 13 as he got older, he didn't want them as involved. So they were kind, you know, they found that difficult, definitely.* | **Family understanding of diabetes**  Having a nephew with type one diabetes meant that the mother noted the support was there should she ever require it while also being mindful of her own luck at having a medical background compared to her sibling’s experience of diagnosis. |
|  | **Social Environment** | | |  |
| **Dyad** | **Subthemes** | **Adolescent** | **Parent** | **Dyadic Code/Summary** |
| 7 | **Impact on interest levels & participation** | Noted more involved in group activities (lines 278-279): *No, I was always like really interested in like group kind of things and always like go to hockey like it never really interfered with that* but no hobbies were really changed by diabetes (lines 259-260): *I do like reading and stuff it wouldn't like interfere like anything like that?* | Diabetes allowed to put life into perspective and the pull back from her career (lines 561-566): *I had no time for reading books or doing any hobbies (laugh) at that time.* *So actually, diabetes is probably done me a favour…Because I've pulled back on all of that career and I've had more time now to do the gym and I'm playing more golf than I used to and you know, all of that have been kind of put on hold.* Noted initially hesitant to have both parents leave child but this dissipated as confidence grew in diabetes management (lines 597-599): *Probably the first year I might have been a bit more ehm, you know, worried you might been away for too long, but certainly not now. Now it's no problem.*  OVERLAP WITH DIABETES VISIBILITY | **Diabetes as putting life into perspective**  While the adolescent noted not letting diabetes impact the activities she is passionate about, the mother noted diabetes allowed her to re-find her personal interests and take a step back from her career. |
|  | **Psychological Distress** | | |  |
| **Dyad** | **Subthemes** | **Adolescent** | **Parent** | **Dyadic Code/Summary** |
| 7 | **Mental toll of Diabetes** | Increased worrying post diagnosis and period of self-consciousness with diabetes engagement in public but this has gradually lessened (lines 299-301): *I think I'd be like I went through like a phase of being, a bit like more conscious of stuff, but now I'm kind of like, that was only like when I first got diagnosed, but that was like a long time ago.* | Noted feeling pressure from a medical perspective at not being able to do more for her daughter meaning she puts a lot of time and energy into pushing the national diabetes programme to evolve (lines 614-620): *I would worry. The main worry is is like not being able to do more to help you know. But you know, that's just I am trying to do my best to try and see is there a way of getting clinical trials to Ireland, all that kind of things would have been putting my feelers out on that. So that's really that's more, it's more like, you know, more should be done. It's 100 years since insulin was invented, you know…It should be moving on. That's where I'd find, I'd be frustrated, you know.* | **Adolescent acclimatisation and parental self-pressure**  While the adolescent noted an initial reactive period of increased worrying following diagnosis, they not noted feeling more confident in their diabetes care. However, the mother noted feeling pressure to improve the diabetes systems within Ireland as she cannot aid her daughter directly anymore with the shift from parental to self-management. |
|  | **Diabetes visibility** | Noted the technology as making diabetes a visible condition (lines 307-308): *Just like obviously like the pump and stuff. Like… you can see that, but not like anything else.* | Again, noted diabetes as putting life into perspective and making her slow down in her career (lines 629-630): *No, I'm definitely more rested. That's what's the funny thing is, I was like, going hell for leather and Grace's diagnosis made me stop.*  OVERLAP WITH INTEREST LEVELS AND PARTICIPATION | **Visible technology**  The adolescent noted the visibility of the technology she uses for daily management while the mother noted feeling her appearance is more rested as diabetes has put life into perspective. |
|  | **Other** | N/A | Noted peer perceptions as influencing diabetes management (lines 645-648): *How would other people feel, you know, somebody who's very shy or, you know, that kind of area, that whole anxiety of dealing with the disease and your pencil case full of all your injections and going into school?* and wondering about teenage self-consciousness as potentially impacting wearing technology although this didn’t happen with her daughter (lines 643-644): *So things like that that I've thought were going to be big obstacle, ended up being fine.* | **Anxiety and diabetes**  The mother highlighted a lot of worries she had about her daughter’s management within social settings and while they didn’t come to fruition for her daughter, she noted the potential for them occurring for other young girls diagnosed with diabetes. |

|  | **Theme** | | |  |
| --- | --- | --- | --- | --- |
|  | **Illness Representations of Diabetes** | | |  |
| **Dyad** | **Subthemes** | **Adolescent** | **Parent** | **Dyadic Code/Summary** |
| **8** | **Diabetes Awareness** | Lack of insulin production acknowledged but no mention of auto immune condition (line 11): *When the pancreas stops producing insulin.* Awareness of changing bodily needs as she ages and the need for monitoring throughout these BG fluctuations (lines 18-19): *Well growing up and stuff my bloods are going to like start changing. So I have to keep an eye on them, especially when I'm out.* Noted serious nature of the condition highlighting the need for balance of BGs and care for highs and lows (lines 27-30): *Well I mean, if you're not checking your bloods and they just go high or low, you can just faint...And if no one sees you, you're gone.* | Lack of insulin production acknowledged but no mention of auto immune condition (line 12): *It's when the pancreas stopped working and produces insulin.* Noted her and her spouses lives as centred around their daughter with the mother as the primary caregiver so with the diabetes oversight and responsibility largely falling on her (lines 98-100): *So we're we're kind of always need to be on hand and especially if she's sick in school, if she’s anxiety, if she’s not feeling well, play dates stuff like sleepovers, or stuff like that I have to be well aware the parents knows exactly what's involved.*  Noted the amount of oversight and responsibility she still has despite her daughters age (lines 541-546): *So she’d have her sleepovers, she will go to friends’ houses. I just, if she’s out or away, in a, in a parent that I wouldn’t necessarily know…*  *I wouldn’t have a drink and I’d stay close, or like with any parties I’d never leave the area of the birthday party like stuff, I’s always try my part to stay but like as she got older she didn’t want me to stay at a birthday party with her?* | **Management as key**  Similar descriptions across the dyad of what is diabetes indicating a level of shared understanding about the illness. Both members of the dyad noted the potentially fatal nature of the illness if unmanaged highlighting consistent monitoring as key to managing the illness. The mother also highlighted her role as primary caregiver in ensuring others around her daughter have a diabetes awareness. |
|  | **Emotional Perceptions and Identity** | No memories of life pre-diabetes making it a normal aspect of life (lines 14-15): *I don't really remember not having it so I kind of… I don’t know. I just kind of live with it now.* Noted some frustration within school with potential exclusion for physical activity due to BGs (lines 34-36): *Ehm it doesn't affect much I mean like I might have my SnA (Special Needs Assistant) in school, so like in PE it’s a bit annoying when you have to go off and make sure you're not low.* Some worries about future management and the need for continued vigilance with diabetes (line 41): *Sometimes I'm scared, like in the future, if I get careless or anything.*  OVERLAP WITH VOCATIONAL PERFORMANCE | Huge shock and major adjustment when first diagnosed (lines 15-17): *I suppose we are okay with it now, but at the start I I actually it was life changing for, for us. And because she was only two months turned two when she was diagnosed ehm it was just a shock.* Noted now with hindsight while the adjustment was difficult (line 134): *Eighteen months, we didn't leave her,* she wouldn’t have catastrophised as much back then with her current knowledge (lines 22-26): *And to be honest, if I knew what I know now, I probably wouldn't have been as worried...But you do think when a child has a a lifelong illness, that it is going to affect their life in the most horrible way, and it really hasn't, like she, she is a little trooper.* Noted keeping up to date with research around cures and staying hopeful for the future (lines 33-34): *Well, I'm. I'm hoping I'm still hopeful there's going to be a cure at some stage. Like I I do read up on these things. But so far, there's nothing.* Noted the shift from parental to self-management as easing the parental concerns due to confidence in their child and her sensible nature (lines 109-111): *Now I will say like in the last couple of years it's it's been easier. We're not so on tender hooks, she has a little bit more freedom because she, she’s a very sensible child, she’s very capable of looking after herself.* Parental support groups as helping her to accept and learn to live with diabetes while not catastrophizing (lines 171-174): *They did help me accept it a lot more because I wasn't accepting it at the start, because I was thinking like Helena wouldn’t maybe you know live to grow up and think I thought you do have these mad going around in your head that scare you.*  OVERLAP WITH FUTURE EXPECTATIONS AND INFORMATION SEEKING | **Diabetes integration and fear**  Across the dyad fear was noted in terms of the emotional load of diabetes. The adolescent commented on fear of becoming careless with her management in the future and what that might mean for her health while the mother reminisced on the fear post-diagnosis and how much catastrophizing she did. However, both members of the dyad noted that it has become easier to manage over the years with the adolescent likening it to second nature while the mother commended her daughter’s attitude to diabetes and just treating it like another aspect of her day. |
|  | **Healthcare Orientation** | | |  |
| **Dyad** | **Subthemes** | **Adolescent** | **Parent** | **Dyadic Code/Summary** |
| 8 | **Attitudes towards healthcare** | Diagnosed so young, no known affect on attitudes toward health. Noted healthcare team as addressing treatment options to the parent not her and not listening to her own opinions (lines 70-72): *I think there are all quite nice. But…I feel like somethings they're quite pushy about.* | Health conscious prior to diagnosis meaning not much adjustment required for food prep (lines 184-186): *We kind of would, my husband is like a fitness fanatic, and we kind of would have always ate healthy and everything and I think that’s what kinda helped Helena at the start.* Noted the healthcare team as pushing their opinions on treatment onto her daughter despite her self-consciousness (lines 236-237): *I think I know that the hospital is trying to push Helena to go on the Dexcom and she is dead for against it because it's too big.* Noted recurrent issue with staff addressing mother rather than adolescent and not listening to the actual patient (lines 240-245): *Children have their own mind, they know their own bodies and they're taught to-to use their mind to tell people what they think to take care of their own health. Especially if you have an illness so I do think they should listen to the child a little bit more…Because when Helena tells them no, they they look at me go “Well mum, this the better thing”. Helena has said no, she’s 12.* | **Respect for Adolescent views**  There was congruence across the dyad that while the healthcare team are generally regarded as nice, that they lack respect for the views of the adolescent. The adolescent felt that they addressed things to the mother and were pushing her to go on a treatment option she didn’t like. The mother agreed that the team had been trying to go around the adolescent to get her to push her child for the desired treatment option. While the mother noted in a life-or-death situation, she would step in she also advocated for her daughters right to have an opinion and know her own body.  OVERLAP WITH DIABETES VISIBILITY |
|  | **Diabetes Management** | Awareness of the serious nature of the illness and need for constant management with no breaks from the illness (lines 63-67): *It's quite a big responsibility because it it's it's to do with your life… and your wellbeing. So I suppose you need to, be responsible about it and try and like when something is bad, you cant just sit there and like, not, not do anything about it.* Reported technology as easier to fit into her life and despite the visibility of it, having the smaller pump means she is less self-conscious when it is on show (lines 81-83): *I like the pumps a lot better than the needles…It’s just like, I mean, it's just a lot easier.*  OVERLAP WITH DIABETES VISIBILITY | Noted changing management needs as adolescent has more autonomy and independence in food choices. However, noted that adolescent awareness of her body and management needs means this isn’t too difficult as a mother (lines 195-200): *Helena will tell you straight out that certain sugar or certain Jelly, she won't eat because she doesn't like the way it makes her feel because she can't control…She can’t bolus enough, she doesn't know what the bolus is enough. So she'll stay on the safe side.* | **Diabetes as fitting their lives**  The adolescent spoke of the constant responsibility and need for management that accompanies diabetes. She noted that having smaller or more discrete technology helped her self-consciousness. The mother focused on the transition from parental to self-management and the ease with which this is occurring due to the adolescent’s sensible nature and her patterns of following trends she knows how to manage. |
|  | **Future expectations** | No real thoughts on future with diabetes but awareness of being more mindful than her peers (lines 94-96): *I don't think it's gonna change much from from from other people...When they grow up I suppose, you just need to be a bit more careful.* | Noted keeping up to date with research around cures and staying hopeful for the future (lines 33-34): *Well, I'm. I'm hoping I'm still hopeful there's going to be a cure at some stage. Like I I do read up on these things. But so far, there's nothing.*  OVERLAP WITH EMOTIONAL PERCEPTIONS AND IDENTITY, AND INFORMATION SEEKING | **Life and Hope**  The adolescent commented on the lack of expectations regarding her diabetes she holds. She noted an awareness of being more mindful than her peers highlighting the ingrained management within her daily life. The adult on the other hand reported information seeking about cures and hoping for a future without diabetes. |
|  | **Information seeking** | Lack of need for information seeking due to young age at diagnosis and learning diabetes management as she grew (lines 100-101): *No, not really. Like I mean mom and dad have given me most of it, when I was like, younger.* No independent treatment related information seeking but highlighted querying fit of technology within the clinic environment (lines 109-110): *I don't now, like at home, but I do ask couple questions in the hospital if we're changing pumps or like insulins.* | Awareness of technology and benefits but also noting that it should be self-management or respecting the adolescents wishes (lines 251-254): *Like we said, we we'll address this again in a few months and see how to change the mind. I know the Dexcom is better than the Libra…But Helena doesn’t want it.*  OVERLAP WITH EMOTIONAL PERCEPTIONS AND IDENTITY, AND FUTURE EXPECTATIONS | **Diabetes as fitting the adolescent’s life**  There was an element of agreement reported on this subtheme with regards to seeking information around the correct fit for the adolescent’s lifestyle. The mother noted having the information herself but noting pushing her daughter to make a choice she doesn’t want to while the adolescent highlighted querying with the healthcare team about the fit of technology within her wants (e.g. discrete). |
|  | **Impact on the Vocational Environment** | | |  |
| **Dyad** | **Subthemes** | **Adolescent** | **Parent** | **Dyadic Code/Summary** |
| 8 | **Vocational Performance** | School support from SnA as helpful but experiences the monitoring during PE as exclusionary and frustrating (lines 34-36): *Ehm it doesn't affect much I mean like I might have my SnA (Special Needs Assistant) in school, so like in PE it’s a bit annoying when you have to go off and make sure you're not low.* Noted missing some school days recently due to a high noting the dangerous nature of balancing ketones (lines 120-125): *I did lose two days cause I got quite, I didn't feel well one day in school so I skipped lunch because I didn't feel well…And then I got and starvation ketones…Which caused me to feel even un-even sicker.* Noted good peer support in the school environment (lines 140-141): *If we have a like a substitute, they’ll inform the teacher if I’m not feeling well, what’s wrong. So they are all pretty good about it.*  OVERLAP WITH EMOTIONAL PERCEPTIONS AND IDENTITY | Had to change jobs when adolescent began on pump therapy due to the increased information and parental demands at that time (lines 284-285): *When she was first diagnosed. I tried to stay in work and then when she went pumping, it wasn't working.* Reported the stress of balancing a job and managing diabetes was too difficult (lines 287-290): *If I got caught in traffic, that could take me 40 minutes. I couldn't do the stress of trying to get her…So I gave up work for good few years.* Changed her job when she went back to work- noted this new company is much more flexible and provides her with the ability to be there should her daughter need her (lines 333): *If I need to work at home when she's not well, I set up from home.* | **Vocational support as making life easier**  Both members of the dyad commented on the experiences of support within their vocational environments. The adolescent commented on the SnA and her peers as making her feel supported throughout school while noting the difficulty of managing diabetes when she is ill. The mother noted having to leave her job post-diagnosis due to the stress of balancing diabetes with life and subsequently finding a job that supports her and encourage flexibility to be there for her daughter. |
|  | **Vocational Values** | Reported believing school would still be as important to her if she didn’t have diabetes. | Awareness of lack of importance in job now post-diagnosis (lines 383-384): *It’s lovely but… absolutely not. If I had to leave it tomorrow for one of the kids, I’d leave it.* | **Children over job**  While the adolescent had no real opinion on this matter due to her young age at diagnosis, the mother highlighted that diabetes put life into perspective for both of her children and caused her to reprioritise her life. |
|  | **Impact on the Home Environment** | | |  |
| **Dyad** | **Subthemes** | **Adolescent** | **Parent** | **Dyadic Code/Summary** |
| 8 | **Impact on Relationships** | Close relationship with mother and more light-heated bond with father (lines 156-157): *I suppose me and my dad have a bit more of a funnier relationship and it’s not really like a serious one.* | Extremely close relationship with adolescent noting the family jokes at the closeness of their bond (lines 394-395): *Very close. Very, very close, she, she’s, Noelle (sister) and John (husband) say she would live in my skin if she could.* Difficult adjustment for sibling at the time of diagnosis but noted changing jobs herself meant she was home for both children (lines 349-353): *Helena’s first couple of years was very hard on Noelle (sister)…Very hard. Yeah…I think other people forget about the other children in the house.* Spousal relationship strain post-diagnosis with mother as primary caregiver burdening the responsibility for the diabetes (lines 414-416): *At the start, when Helena was first diagnosed myself and John, found it very hard to, I just thought he wasn't there and he didn't understand, he thought I overreacted. That was a strain for about a year or so.* Change in household conversation (lines 437-438): *Everything was centred around Helena and diabetes for at least 18 months to two years I would say.* | **Primary caregiver responsibility and strain**  There was congruence across the dyad about their very close bond with each other. While the adolescent noted good relationships with both her parents she did highlight the less serious relationship with her father. This highlights the impact of the mother being the primary caregiver and having the diabetes responsibility to shoulder as leading to a more “serious” relationship than with the father. Similarly, the mother highlighted how she struggled with her position as the primary caregiver and the lack of understanding from her husband post-diagnosis emphasizing both the strain and added bond that resulted from this parental role. |
|  | **Quality of Support** | Noted mother as a great practical support as well when running low (lines 177-178): *If I were to need it, my mom would probably do it like, if I come in from school and I don't, I feel quite low and she usually like, just do the dishes or the hoovering for me.* | Great wider network of support available to them (line 477): *I honestly think we were very, very lucky. We had a great network around us.* | **Immediate versus wider family support**  While the adolescent noted her mother would support her with anything she needed the mother herself focussed on her wider network in her sister and mother-in-law for practical diabetes support showing the differing experiences of the immediate family. |
|  | **Impact on Family life** | Awareness of amount of supplies needed for diabetes management but noted the LTI scheme stops any financial burden (lines 185-187): *We don’t pay for all of our, like ehm the cannulas and stuff…But if we were to, I, I’d say there would be trouble yeah.* | Noted the timing of food during the first few years as very difficult for the whole family to adjust to (lines 446-449): *Make her eat it real quick, so she would be okay in time to have her next dinner…and you’d be rushing Noelle (sister) as well.* The whole family had to contingency plan to leave the house during the first few years due to the added labour involved with feeding regimes (lines 464-469): *It was like having a new-born baby again when you left the house, you needed a whole backpac…You need Jellies, you needed Lucozade, you needed snacks, you needed sugar free drinks, you needed water, you needed… It was. Yeah. It was like packing for a new-born, baby.* Benefit of the hospital applying for all the technology meant no real financial demands to diabetes (lines501-502): *The pump and all that the hospital got for us.* | **No financial impacts but initial family needs**  While both members of the dyad commented on the LTI scheme as resulting in no financial burden of diabetes, the mother also discussed food and mealtimes becoming more laboursome with diabetes. Food was noted as being more stressful and affecting the whole family in the early days leading to less time spent outside of the home initially due to the planning required. |
|  | **Extended Family Relationships** | | |  |
| **Dyad** | **Subthemes** | **Adolescent** | **Parent** | **Dyadic Code/Summary** |
| **8** | **Extended family communication** | No affect of diabetes on the wider family communication or engagement. | Increase in communication and support post diagnosis (lines 509-511): *If anything we heard more from my parents and John’s (husbands) parents, they were always willing to help, always willing to collect Helena and stuff, you know, you know, never found an issue.*  OVERLAP WITH EXTENDED FAMILY QUALITY OF SUPPORT | **Increased support**  While the adolescent just commented on no perceived impact of her diabetes on wider family engagement, the mother reported an increase in both communication and support. This highlights potentially the lack of awareness from the adolescent of life pre-diabetes. |
|  | **Extended family quality of support** | Good support from paternal grandmother reported. | Parent’s sister and paternal grandmother as huge supports physically from a diabetes perspective (lines 486-488): *She had her own blood kit, had her own insulin pens. She, so if you were packing up to go to Margaret (aunt), she didn't need all that cause it was always there.* Noted one good friend as having really close bond with adolescent and being able to help change cannulas when her own father cannot providing great support and peace of mind for the parents (lines 523-526): *We can kind of go and take a night or two nights away, and you know if anything happens, happened, if the cannula failed or fell off, she’s only around the corner and she can come over. Same if my mam was staying or Caroline (granny) was babysitting her.* Noted reduced engagement with family directly following diagnosis due to parental fear (lines 529-533): *For me, for me and John (husband) all, but then we were nervous at the start. We, we didn’t want to leave her. We were always afraid something would happen, but I think w-we kind of did that to ourselves…*  OVERLAP WITH IMPACT ON INTEREST LEVELS & PARTICIPATION AND EXTENDED FAMILY COMMUNICATION | **Wide diabetes support network**  While the adolescent merely named her paternal grandmother as a good source of support, the mother went into detail of how different family and friends have learned about diabetes and now support the adolescent in practical ways. Noted site changes that even her own husband can’t help their daughter with as being supported by a family friend. Also noted parental concerns post-diagnosis as hindering their engagement for fear of something going wrong.  OVERLAP WITH EMOTIONAL PERCEPTIONS AND IDENTITY |
|  | **Social Environment** | | |  |
| **Dyad** | **Subthemes** | **Adolescent** | **Parent** | **Dyadic Code/Summary** |
| 8 | **Impact on interest levels & participation** | Noted no affect of diabetes on interest in hobbies but rather aging out of activities (lines 225-226): *I suppose it hasn't affected any, any of it, but as I got older, I kind of lost interest in a good bit of things.* Reported participation in social activities not hindered by diabetes though she does find more fluctuations when out with her friends (lines 235-237): *Ehm well, I suppose it only when I go my friends, I do get, I get a bit high cause ehm or a low, a little especially if we are going shopping and all the walks around but it hasn't affected like socializing or anything.* | Noted life as hindering hobbies not just diabetes citing her other daughter, competing demands, and the death of her sister as all influencing her interest and participations levels (lines 557-566): *Only kind of getting that back now. Well it wasn’t just because of Helena, like I, I, have another daughter that is three years older, and her life kind of, she needed me there as well just, I lost my sister this day four years ago…And at that time, yeah I didn’t want to do anything then…But I don’t think it was totally Helena that stopped that, totally the diabetes I think just kind of life kind of stopped that.* Noted increase in family activities due to wanting to be around adolescent and keep an eye on things (line 573): *And it was more I didn’t want to leave the kids.* Reduction in spousal activities due to fear potentially unwarranted, at leaving the adolescent alone (lines 602-607): *I wasn’t comfortable if the two of us went out…Ehm because I always wanted somebody to be here- Either me or John (husband) but again I think that is something we did ourselves maybe looking back ehm we-we didn’t need to do it?*  OVERLAP WITH EMOTIONAL PERCEPTIONS AND IDENTITY | **Changes in social engagement**  Differing views were reported regarding social environment engagement since diagnosis. The adolescent reported growing out of hobbies and BG fluctuations as occurring more when out with peers. The mother on the other hand noted reverting post-diagnosis to family only activities for fear of leaving her daughter though she also noted that overall engagement and interest weaned due to bereavement and parental stress. This highlights the differing effects of diabetes as needing more monitoring when with peers versus withdrawal due to changing life circumstances. |
|  | **Psychological Distress** | | |  |
| **Dyad** | **Subthemes** | **Adolescent** | **Parent** | **Dyadic Code/Summary** |
| 8 | **Mental toll of Diabetes** | Anxiety around something other than diabetes having knock-on effects on diabetes (lines 247-252): *I’d be quite nervous because there's been a few stuffs with this one girl and it's you know…Yeah, but it then it makes my blood go, like quite high. Like maybe…25.* | Anxiety over pubertal changes affecting diabetes management (lines 616-618): *Well… yeah because she’s starting to hit puberty now and everything’s changing for her and now we had a control over her diabetes for so long, and we’ve kind of lost a bit of it then for a while and we couldn’t get the bloods under control.* Noted the loss of stability recently as being very anxiety provoking and highlighting the never-ending need for management (lines 629-630): *You kind of don’t rest, you don’t sleep, you know the diabetes is just always there.* Reported sadness over the fact her daughter has diabetes and doesn’t remember life without it (lines 633-635): *Not hopeless or depressed but a little bit sad that she is going through it, that she does have it and that she doesn’t remember life without it.* However, acknowledging that her daughter deals better with the diabetes now than she does (lines 639-641): *I think it upsets me more than her now to be fair…Like she just gets on with it.* Noted the anger at diagnosis and the mixed sadness she still feels now when seeing the physical scars from the medication and the emotional toll of the BG fluctuations (lines 645-653): *I was very angry that it happened to her, that it happened to her when she was so young and because she was so sick. I, I’m I’m not as angry, as I I would’ve been at the very very start but I do feel angry, like I get…Angry when I see her little tummy with all the lumps in it, I don’t know if its angry or sad or maybe a bit of both would, ehm…Or when I see her struggling so much or she’s getting excited to do something and all of a sudden she’s got low, or she’s gone high or then cause she’s anxious or nervous.* Highlighted the maternal guilt at her daughter’s illness that was compounded by her own illness during pregnancy (lines 660-661): *That’s a mother thing isn’t it? Especially because I have, I had the gestational d- diabetes I think I did think it was my fault.* | **Emotional cycle with diabetes**  Both the adolescent and the mother reported on the impact of diabetes and emotions. The adolescent discussed anxiety with other aspects of her life that have subsequent knock-on effects on her diabetes management which in turn stresses her out. The mother on the other hand highlighted the weight of the parental responsibility and the emotional burden she carries since the diagnosis. While the mother noted her range of emotions from sadness to anger to guilt, she also acknowledged how her daughter copes much better than her as she doesn’t remember life pre-diabetes. This distinct lack of diabetes related emotions reported from the adolescent corroborates this idea that diabetes is more of an emotional toll on the parent than the child who becomes accustomed to this way of life and knows no different. |
|  | **Diabetes visibility** | Peer comments as affecting confidence (lines 275-276): *There was one girl, when I had the cannula somewhere else, she would always tells me it looks like a pimple. That made me a bit more conscious of it.* Noted the smaller technology while still visible as making her less self-conscious (lines 86-90): *The Ipso pump I have now is a lot smaller than the Animas one…Which makes it, which makes it, makes me feel quite better and it’s like in this, like on holidays when you're wearing like your swimsuit and stuff…*  OVERLAP WITH DIABETES MANAGEMENT | No physical affects of diabetes on her appearance reported. | **Discrete diabetes technology**  The adolescent commented on her desire to have discrete or smaller technology in order to feel less self-conscious about her diabetes. This was further highlighted by the mother noting the child’s wishes regarding technology should be respected even if it is for superficial reasons such as a smaller sensor.  OVERLAP WITH ATTITUDES TOWARDS HEALTHCARE |
|  | **Other** | N/A | Reiterated point about respecting child’s wishes.  OVERLAP WITH ATTITUDES TOWARDS HEALTHCARE | OVERLAP WITH ATTITUDES TOWARDS HEALTHCARE |

|  | **Theme** | | |  |
| --- | --- | --- | --- | --- |
|  | **Illness Representations of Diabetes** | | |  |
| **Dyad** | **Subthemes** | **Adolescent** | **Parent** | **Dyadic Code/Summary** |
| **9** | **Diabetes Awareness** | Some understanding of the illness but some misconceptions about being born with diabetes and no mention of auto immune nature or need for artificial insulin (lines 15-16): *It is a medical condition that you get when your pancreas stops working. Ehm, you're usually born with it. You don't really have anything to do with getting it.* Some awareness of potential complications of the illness (25-27): *Can be pretty serious if you don't take care of it correctly. It could lead to losing your foot or heart disease? Yeah, stuff like that.* Diabetes as not stopping him do anything just requiring frequent management (lines 36-38): *Making sure you eat like the exact amount of …And getting enough insulin. Making sure you don't put too much or too little.* | Good understanding of diabetes noting the need for insulin but no mention of auto immune condition (line 11): *It’s when the pancreas ehm stopped working to create insulin to the body.* Lack of diabetes awareness and knowledge about treatment made for a difficult adjustment post-diagnosis (lines 15-17): *Well in the beginning, it was tough… Not to know because we have no experience at all in about the treatment but noted having to move forward with diabetes.* Noted first query to the doctor post-diagnosis was about the duration of the illness (lines 38-40): *That was my first question, you know? …The doctor told me, how long is going to be the treatment? Oh forever.* Long- and short-term need for management and potential complications noted (lines 43-48): *Very serious…Because in long term we have a very consequence, If if you don't take care about it the diabetes…If it's not in the long, in the short term. If you a low sugar, you can die.* As an immigrant working shift work, found post-diagnosis and the learning period as difficult trying to adapt food and meal times for diabetes while still going to work (lines 77-86): *At the beginning we start, you know we have to eat at the same time, the same food, the same…Was really hard, you know because …(pause)… as immigrant…We have to change the we, have to work anytime…You don't come choose especially, your roster is moving and things so…We have to adapt this for them.* | **Tough adjustment to normal aspect of life**  While the adolescent focused on how diabetes doesn’t stop him doing anything if he continues to manage it, the parent noted the initial adjustment after diagnosis was difficult due to a lack of awareness about diabetes at the time. Noted the amount of juggling they had to do to coordinate work schedules for the needed routine. While both members of the dyad had similar levels of awareness reported now, the father’s reporting of the difficulties at diagnosis highlight the parental burden of management post diagnosis.  OVERLAP WITH EMOTIONAL PERCEPTIONS AND IDENTITY |
|  | **Emotional Perceptions and Identity** | Diabetes acceptance as a part of life now (line 19): *It's not really an inconvenience, it's just something that's there.* Reported feeling good about living with diabetes noting it as a lifestyle rather than any major change (line41): *Don't look at it like a disease, just as a way of living.* | Son having seizure from severe hypos as causing huge parental fear and concern (line 66): *Terrible, terrible, terrible.* Reported adjustment period as difficult and using his faith to get through it (lines 95-96): *Well, at the beginning, you have tough moments and you know, yeah, kind of the prayers.* | **Adolescent acceptance versus parental worry**  Similar to above, across the dyad the adolescent discussed diabetes as not having a major effect on his life while the father noted the emotional burden the parents had to shoulder at diagnosis and the fear with hypos since.  OVERLAP WITH DIABETES AWARENESS |
|  | **Healthcare Orientation** | | |  |
| **Dyad** | **Subthemes** | **Adolescent** | **Parent** | **Dyadic Code/Summary** |
| 9 | **Attitudes towards healthcare** | Not conscious of his health prior to the diagnosis noting a newfound awareness now (lines 55-57): *So it like makes you more aware that there's actually a lot more dangers, with…With like what you do to your body.* Good experience with healthcare staff (line 75): *They try and do the best they can for you.* | Personal journey from fitness to simply not having time for it meant he was previously health conscious and having to return to that place now that his son has diabetes (lines 125-127): *So I do, I know the benefit of the healthy...The style of life. And now we have return to it, regardless.* Noted supportive accessible healthcare experience with lots of compassion during the two seizure episodes (lines 154-155): *I'm really grateful for they have that that brain because they explain us really well.* | **Health consciousness**  Both members of the dyad noted good experiences with the healthcare team as well as commenting on the increase in health consciousness that came about after diagnosis. While the adolescent noted no real attitude towards his own health prior to his diagnosis he noted this newfound awareness while the father noted having been health conscious and diabetes reaffirming this and pushing the family back to a better place health wise. |
|  | **Diabetes Management** | Food as focus of diabetes management noting food as being restricted and more thought about now (lines 67-69): *Ohh, what you eat. Definitely what you eat and how much you eat of it…You can have chocolate but just not the whole bar… like a line…* Noted frustration at the current treatment plan (MDIs) and the effect this has on his hobbies (lines 82-85): *They can get annoying…*  *Especially since I play a lot of basketball, so I have to like, go quickly so I can go back into the game.* | Prior to sensor noted parental management during the night as laboursome with frequent monitoring but noted the ongoing management needs still required from him in terms of monitoring, carrying supplies and checking in with his son (lines 138-142): *Always checking out if they should work, if he he feel the easiest answer, we have to check…All the time. Always being with sugar or Lucozade, or anything that frequently has or extra carbs so it's bigger luggage?* Noted parental responsibility to contingency plan and always monitor how his son is feeling (lines 146-147): *We have to get prepared for everything happen, you know?* Reporting MDIs as a fine method of management for some people, noted his opinion that technology should be available to everyone as the best standard of care available (line 188): *For me, you have to start everybody, should we have the Dexcom, the pump?* | **Pump as the next step**  Across the dyad both members noted that while the pens are the first step post-diagnosis in order to learn about diabetes, that they are ready and waiting for the pump technology and the ease this would provide. The father noted the multiple parental demands in terms of carrying supplies and food with the adolescent agreeing about the added labour of food and mealtimes.  OVERLAP WITH INFORMATION SEEKING |
|  | **Future expectations** | No real future expectations noted but did report diabetes as non-negotiable aspect of life so better to just accept it and live his life (lines 91-92): *You don't really know, just it's gonna be with me my whole life, so might as well embrace it…* | Noted the need for building adolescent awareness for an eventual transition from parental to self-management and hoping his son will take ownership over his health (lines 192-198): *Well from my point of view, my work is prepare him to take care himself…You don't know. I can't be with him for all his life. So I have to…Be, make sure he's aware what is his training, how have to do what you have to do…* | **Parental to Self-management**  While the adolescent noted embracing diabetes as a normal aspect of his life for his future the father focussed on the more practical expectations around transitioning care to his son. |
|  | **Information seeking** | Not much information seeking but noted hopeful about technology and wanting to know more about these options (lines 101-103): *I wish to like know like the newest things and the most successful ones…Out of all of them. That would be pretty cool.* Reported investigating the pump (next step in treatment plan hopefully) and potential cures (lines 109-114): *I forgot what it's called, but it's like this medicine kind of, that was tested…And apparently got, like, rid of diabetes.* | Initially taking information from medical staff and gradual move to information seeking and parental peer support groups (lines 206-211): *Well at the beginning, I just follow what the doctor say…Then we start to investigate for ourselves. Then there is a lot of podcasts or…Information in the multimedia. Ehm… ehm we create contact with another parents…* Noted his current research about the pumps as best standard of care and pushing the team to place his son on a waitlist for one (line 227): *Always, always. If it’s something new, I asking them, yeah always.* | **Pump as way forward**  Again, both members of the dyad focused on the pump as the next step for treatment with both acknowledging frequent information seeking around the technology. While the adolescent also noted hope for a cure the father didn’t mention this. The father did however mention his information seeking through parental peer support.  OVERLAP WITH DIABETES MANAGEMENT |
|  | **Impact on the Vocational Environment** | | |  |
| **Dyad** | **Subthemes** | **Adolescent** | **Parent** | **Dyadic Code/Summary** |
| 9 | **Vocational Performance** | Noted no major impacts of diabetes on schoolwork but highlighted a hypo as affecting concentration and having a detrimental affect on exams (lines 124-125): *Except in a test, if you get a low blood sugar in a test you're completely screwed.* | Need to adapt work schedules to align with changing diabetic needs particularly with school holidays and change in routine (line 240): *Yeah, we'll have to try to adopt my roster and his time.* | **Diabetes responsibility**  While the parent noted no direct influence of diabetes on his performance at work expect for needing to adapt rosters to suit diabetes needs, the adolescent noted the physical toll of BG fluctuations in the school setting. This highlights the parental responsibility of having to be at home for his son and mealtimes while the adolescent focuses on the personal responsibility of his grades being potentially affected. |
|  | **Vocational Values** | No impact of diabetes on school importance or academic goals (line 148): *No. Same as always.* | No impact of diabetes on work importance or goals (line 367): *That's fine. Yeah, has to be, yeah* | **No impact on vocational values.**  Congruence across the dyad that there was no impact of diabetes on school/job importance or goals highlighting the need to still live your life with diabetes. |
|  | **Impact on the Home Environment** | | |  |
| **Dyad** | **Subthemes** | **Adolescent** | **Parent** | **Dyadic Code/Summary** |
| 9 | **Impact on Relationships** | Noted a “fun” relationship with his father (line 162): *Very fun. Takes care of me. He’s a father.* Which was juxtaposed with the more serious and protective relationship with his mother (line 176): *No, I'm still her 5-year-old.* Noted increase in familial communication since diagnosis to include diabetes as a topic as well (lines 195-201): *I think it increased from before I was diagnosed…Like I would like just have normal conversation like my day was good, how was yours?…Stuff like that but not like how did you treat your sugars today? Did you do it well? Stuff like that, extra like.* | Good strong bond with his son though acknowledging potentially being overprotective due to parental monitoring (lines 284-287): *Maybe we are a little bit more overprotective to him…You know. Sometimes, checking over, checking him. What he's doing, what he's done doing what you eating. “Why you eating this? What you doing?”.* Noted his relationship with his wife has shifted as she has become more focussed on their son again (lines 294-296): *My wife having more affected than me in…About this. She has more stressful.* Increased communication as another topic for the household. | **Protective parents**  Congruence across the dyad with regard to the increase in familial communication and additional topic that diabetes provides through parental check-ins. There was also agreeance with the level of parental protectiveness that has increased since diagnosis. The adolescent went on to suggest a more serious relationship with his mother which was also confirmed through the father’s report of his wife taking the diagnosis more seriously and experiencing more stress because of it. |
|  | **Quality of Support** | Both parents as willing to help or support him if he asked (line 207): *Definitely.* | Great tag team of support with his wife and they often rely on each other to switch tasks to get things done (line 322): *All the time.* | **Family support**  Both members of the dyad briefly mentioned the family support as available should they request it. |
|  | **Impact on Family life** | Noted swapping out chores for less physically demanding ones in order to not impact the family (lines 179-183): *If I’ve a low sugar, I can't, like do as much physical work…But I will still do some chores…Clean the dishes or something like that.* Noted that he has tried to push through lows before but no longer does that (lines 210-214): *When you have a low sugar you can't like really run…Because you just feel dizzy and you, you'd fall…It's happened to me before (laughs).* Noted being lucky to live in Ireland for the LTI scheme (lines 219-222): *Luckily in Ireland…The healthcare is usually free… there's some extra things you have to pay for, but it's not really like extra. It's not too much.* | Noted lack of family support due to being immigrants as difficult but noted some support from his sister also in Ireland (lines 329-335): *We have the help because one of my sister in Dublin with us…So can do stuff for us all the time...But we don't have too much people, you know, we'll have…Alone here.* Noted the benefit of living in Ireland with regards to the LTI scheme (lines 345-346): *Because here they support almost everything. I don't know, they give you, medicine, everything.*  OVERLAP WITH EXTENDED FAMILY SUPPORT | **Immigration to Ireland as affecting family life**  Across the dyad both members noted the positives and benefits to life in Ireland with regards to the healthcare system and the LTI scheme. However, the father also noted the drawback of less of a support system due to their move to Ireland. |
|  | **Extended Family Relationships** | | |  |
| **Dyad** | **Subthemes** | **Adolescent** | **Parent** | **Dyadic Code/Summary** |
| **9** | **Extended family communication** | Communication with family that also live in Ireland as unchanged by diabetes noting the cautious nature of the wider family and seeking information and reassurance from his mother as to his abilities with diabetes (lines 228-230): *Ehm my two aunts so they're really like cautious about it…They will ask my mom if, like, if can I do this, can I do that.* | Noted the entire wider family contacted him regarding his son following the diagnosis and wanting to learn and be informed about diabetes (lines 363-365): *After he was, increased because my brother have to know…He wants to know what happened.* | **Levels of understanding**  The father noted an increase in communication from the wider family post-diagnosis due to concern and wishing to increase understanding. The adolescent on the other hand reported communication as unchanged but did agree about familial information seeking about diabetes highlighting an additional topic of conversation and the desire to learn. |
|  | **Extended family quality of support** | Proximity of relatives in Ireland as aiding practical support (lines 258-260): *We always like lived together or, like…Really close.* | Noted relatives in Ireland as providing support no questions asked (line 382): *To take care of him and be with him. Yeah, yeah, of course.* Noted that while they may not have the support they would’ve at home, it is available if they need it (lines 287-289): *Having more support fully, but yeah…They we need something they, come over, they going to be able to help.*  OVERLAP WITH IMPACT ON FAMILY LIFE | **Relatives in Ireland as supportive**  Across the dyad both members noted that the majority of their family does not reside in Ireland. The adolescent commented on the relatives in Ireland as living close by meaning there is a close bond and easy access to support. The father agreed with this level of available support from the relatives in Ireland while noting the awareness of the lack of full support due to moving to Ireland. |
|  | **Social Environment** | | |  |
| **Dyad** | **Subthemes** | **Adolescent** | **Parent** | **Dyadic Code/Summary** |
| 9 | **Impact on interest levels & participation** | Increased interest and participation levels with age (line 274): *I've even more interested in them, I participate every day.* Noted no impact on diabetes in any participation or interest levels. | No time for hobbies but interest reported as the same (line 399): *Less time to make the hobby there now.* Noted his son as becoming a teenager affecting family activities more so than diabetes (lines 409-413): *Now you have the teenager… So now you have some parts, but parting is normal…With diabetes or without diabetes.* Noted immigration, busy work schedules and diabetes as all impacting spousal activities (line 424): *Also we are alone here, so somebody have to stay with Issac.* | **Growing up rather than diabetes affecting change**  Both members of the dyad reported the adolescent aging as impacting social activities more than diabetes. However, the father also noted that diabetes combined with other external factors have also had an effect on his time with his wife highlighting prioritising the child as part of the parental responsibility. |
|  | **Psychological Distress** | | |  |
| **Dyad** | **Subthemes** | **Adolescent** | **Parent** | **Dyadic Code/Summary** |
| 9 | **Mental toll of Diabetes** | Reported no impact of diabetes on his emotions in recent times (line 315): *Nothing to do with diabetes.* | Anger and anxiety with his son hiding food and eating restricted foods (lines 443-447): *I came home and Issac found sweet and he eat everything but was hiding it, you know?... And we look after the something around. And when we look I get angry with him, you know, because it look like he doesn't care what happens.* Reported diabetes as an ever-present worry as a parent (lines 480-483): *Now it's more controlled, you know more, you know, more, you have more knowledge…About everything but the same, still worry, you know?* | **Adolescent rebelliousness and parental concern**  While the adolescent reported no emotional reactions to diabetes his father noted that despite the increased knowledge gained since diagnosis this does not negate the parental worry. The father also highlighted that the son has been sneaking “low” treatments when they aren’t home causing him concern about his son’s ownership over his health and diabetes. |
|  | **Diabetes visibility** | Noted the aspects of his technology as being somewhat visible (line 324): *My Dexcom and that?* | No reported impact of diabetes on physical appearance. | N/A |
|  | **Other** | N/A | N/A | N/A |

|  | **Theme** | | |  |
| --- | --- | --- | --- | --- |
|  | **Illness Representations of Diabetes** | | |  |
| **Dyad** | **Subthemes** | **Adolescent** | **Parent** | **Dyadic Code/Summary** |
| **10** | **Diabetes Awareness** | Poor understanding of diabetes but awareness of no cure (line 10): *Type one is… ehm… diabetes that cannot be treated.* Awareness of potentially viral cause of diabetes (line 19-20): *You can have a cold like a few months beforehand, and then you just get it unexpectedly.* Reported serious nature of the illness citing blindness as a potential consequence of poor management, highlighting need for constant monitoring (lines 27-29): *I think it is very serious and… you have to make sure that you… (pause) … you now… (pause)… have to make sure that you keep track of it, because if you don’t it can get very serious and it can get…(pause)... very unhealthy.* Highlighted limb issues as another potential complication of diabetes (line 34): *Having your feet chopped off.* Noted the unpredictability of diabetes as affecting her daily life in terms of trying to manage it (lines 40-42): *Well, you can’t… sometimes it’s just… your…ehm… glucose levels can drop at… the most unexpected times, and unsuitable times, like, when you’re having a test, it can go low… or you can go high or… yeah.* | Good understanding of diabetes referencing need for artificial insulin, auto immune nature, and the need for constant management with overwhelming amounts of data (lines 18-21): *Is…auto immune disease, and she’s dependent to insulin, and she… is counting her carbohydrates… ehm… what’s she’s eating… so then calculating the… the dose of carbohydrates so, she’s always she need check her… level of sugar before eating…ehm… lot of information (laugh).* Noted the adolescent is interested in taking over more management and is learning more about diabetes (lines 46-47): *She knows about the you know the thyroid problem or retinopathy problem or neuropathy.* Noted serious nature of BGs being high or low (lines 120-124): *When sugar is very low, it’s dangerous because because she can’t feel your, she doesn’t have the power to… ask for help somebody, sometimes…And yes of course when sugar is high lot of times, same problem for in the future.* | **Focus on Potential Complications**  Across the dyad while there was a varied level of understanding of diabetes and its cause between the adolescent and her mother, there was a trend of congruence with regards to awareness of potential complications. The mother highlighted the daughter’s interest in moving towards self-management and the responsibility of complication prevention that comes with this. While the adolescent noted there is an unpredictable element to diabetes at time, there was a consensus with regards to consistent management as being key to maintaining a health future. |
|  | **Emotional Perceptions and Identity** | Noted life with diabetes as dictated by the illness and fuelled by worry at having done something wrong (lines 13-15): *Living with type one is… basically… Ehm… living with… having to worry about… what you eat and what you can’t eat and what you have to… check… to make sure you… haven’t done anything wrong…* Highlighted sadness over having to live with the illness and using distraction to try redirect from this (lines 46-47): *I feel very sad like about having type one, it’s… it’s hard for me since I have to live with it forever.* Commented on the never-ending need for management with diabetes and the impact this has on her emotions (lines 61): *Very overwhelmed.* | Familial hope for a cure one day (line 88): *For us it’s a dream, it’s a dream to cure diabetes,* while noting they have been told before that a cure will be found “soon” (lines 105-106): *Hopefully they will find something but ten years old they say it was five years they would find a cure but no.* Highlighted the migration to Ireland as influencing the amount of diabetes knowledge she gained while citing differing healthcare standards, a competing cancer diagnosis and a different language as making for a difficult adjustment period (lines 32-40): *When she came Ireland, was different, so we did a lot of training… and ehm… I ehm… h-had a lot of information, I studied…Most of it from Ireland skills, experience and ehm… yes and ehm… ehm… when we came she was with Leukemia so it was both of them… and ehm… it was hard… To manage because I just came here in one after one… ten days and it was… like… (pause) …nothing, I didn’t know nothing about diabetes but step by step we did a lot of training.* Highlighted the adjustment period post-diagnosis but now accepting diabetes and working to balance it within the rest of life (lines 127-128): *Now, we are fine… we try to find a balance between diabetes and normal life.* Noted the difficulty at watching your child live with diabetes and seeing comparisons with family and the lack of management and responsibility in their lives (lines 155-160): *It’s hard when you compare it to other kids, or when we meet our family, it’s hard because they are eating all and what they want, but she needs before check her sugar, calculate carbohydrates to check her blood, always…The…to weigh the foods and… this is the hard work but we try to optim- be optimist… and keep her really motivated to take in her hands.*  OVERLAP WITH FUTURE EXPECTATIONS, MENTAL TOLL OF DIABETES AND ATTITUDES TOWARDS HEALTHCARE | **Parental and adolescent sadness**  While the mother noted a difficult initial adjustment to the diagnosis, she noted she tries to make diabetes a normal aspect of life but commented on the difficulty comparing her daughter’s life to others her age. The mother noting the hope for a cure also highlights the parental distress at seeing her daughter live with diabetes and the hope this will end one day. Similarly, the adolescent commented on the weight of living with diabetes and not remembering life without it. The congruence over the mental and emotional toll of diabetes with a particular emphasis on sadness was apparent across the dyad. |
|  | **Healthcare Orientation** | | |  |
| **Dyad** | **Subthemes** | **Adolescent** | **Parent** | **Dyadic Code/Summary** |
| 10 | **Attitudes towards healthcare** | Noted vast differences with her carefree peers regarding health-consciousness and the constant management required when living with diabetes (lines 69-74): *Yes… because usually when I’m hanging out with my friends… they don’t really care like… about anything like… usually… I have to watch over how I do stuff, say I… in school I’m having, I’m doing swimming right now…And I have to make sure that… everything’s n-normal… if my blood sugar’s going low… if it is, then I have to make sure. They’re just having fun.* Noted no opinion on her own healthcare team but reported gratitude to doctors researching diabetes (lines 88-89): *I’m happy that the doctors are… eh… they keep researching stuff and finding… stuff out about it.*  OVERLAP WITH DIABETES VISIBILITY | Noted an extremely healthy household and a particular focus since the diagnosis on preventing the long-term complications of diabetes (lines 145-146): *And she really is eating healthy food because we I’m I have paranoia with healthy food, so no fried in my house (laugh), no... so eat much healthy and exercise.* Described the initial diagnosis in her home country as leaving her (line 171): *destroyed* but highlighted her and her husbands jobs as psychologists offered them some insight into not catastrophizing the illness and trying to remain positive for their daughter (lines 174-176): *We are all together and focused all information and… we understand, if we… we will not take under control, we just can give Jessica the… power and motivation and to be you know… umm to look in the future with optimist.* Good experiences with healthcare staff in Ireland as opposed to in home country.  OVERLAP WITH EMOTIONAL PERCEPTIONS AND IDENTITY | **Lack of carefreeness with focus on preventing complications**  There was agreement across the dyad regarding having a good attitude towards personal health and overall health-consciousness. The mother highlighted how this intensified with the diabetes diagnosis and trying to prevent long-term issues for her daughter while the adolescent compared to peers and the carefree nature with which they experience life. The mother also highlighted the emotional devastation at diagnosis and trying to now remain optimistic for their daughter’s future. The adolescent similarly commented on future orientation with regards to researching cures for diabetes. |
|  | **Diabetes Management** | Commented on the physical feelings with BG fluctuations and needing to maintain a relatively stable management in order to feel well (lines 82-85): *I-I know how hard like and dangerous t-type one is, and I feel like if I do… if I leave my blood sugars too low, if I leave my blood sugars too high, I always have to know… I need to take care of it because if I don’t then…(pause)…I don’t feel very nice or good.* Technology as requiring less frequent injections meaning less pain involved overall (lines 101-106): *Sometimes when I’m putting the pump in… like the little…(pause)… Ehm I lost… in what’s… what it’s called but…As I push something in… and sometimes it hurts…But it was a lot better to… than having to take shots every day.* Noted that technology is less painful then MDIs and eases some of the mental toll as well (lines 132-139): *When I used to take shots… I hated it…Because it hurt…But now that I have the pump I feel a lot more… a lot more… relaxed because…I don’t have to worry about taking shots and everything.* | Noted parental guilt over adolescent sickness now referencing poor diabetes management as parental downfall (lines 178-180): *You know when she’s has fevers or something my mind is always “What’s wrong, what I did wrong something”.* Difficulties with management when adolescent was younger due to the pain of MDIs and trying to eat carb-free to avoid injections (lines 201-206): *She sometimes she had some pain … In her legs or her tummy. It was hard when she was… she was- she didn’t want to eat something with carbohydrates because to…Avoid the yes the injection so she was eating just low.* Noted the transition from parental to self-management allowed more flexibility even though it meant more laboursome efforts with food and carb counting for the adolescent (lines 218-221): *She wanted to put herself, so she started to put and… I was very delighted…Because she… and she… eh… had say I just, I told her if you put you can eat more, you will be more flexible…* | **Difficulty at managing diabetes**  Across the dyad there was mention of physical and mental difficulties associated with managing diabetes. The adolescent reported on the physical symptoms of the BG fluctuations as well as the pain of treatment regimes noting that technology removes some of the pain and eases the mental burden slightly. Similarly, the mother noted the pain her daughter experienced with treatment regimes while praising her daughter’s proactive approach to self-management. The mother also noted the emotional toll of parental management through the guilt at causing her daughter to feel unwell and blaming herself for poor management.  OVERLAP WITH EMOTIONAL PERCEPTIONS AND IDENTITY |
|  | **Future expectations** | Noted a familial trait of seeking perfectionism and seeing that in herself for her future with diabetes as well (lines 111-112): *Well, when I take care of my d-diabetes I expect… I… For me like how my personality is, I have to make everything perfect…* | Fear for future complications as a parent carrying the current responsibility for the illness progression (lines 244-254): *I know they can… I know the… the secondary problem after if your sugar is very high…She can get Ret- problems with eyes…Or kidney, or her legs, or her pres-blood pressure… is high…So… I am worried about, like a parent…Know, I am worried, but hopefully will be fine, hopefully we will take under control…* Highlighted again the hope for a cure one day (line 259): *Of course maybe they’ll find some cure.*  OVERLAP WITH EMOTIONAL PERCEPTIONS AND IDENTITY | **Two sides of the future coin**  While the adolescent commented on her need to be perfect including relating to her diabetes, her mother commented on potential complications that could arise from poor management. This highlights the pressure on the daughter to avoid the future complications and how she has internalised this as a goal. Furthermore, while the mother noted hope for a cure, the daughter didn’t comment on this suggesting a more present approach to daily management. |
|  | **Information seeking** | Some infrequent information seeking about treatment (121-122): *Sometimes I search online like… (long pause) … different stuff about diabetes like if there is anything good that I can use…* | Noted frequent information seeking about potential cures and treatment options (lines 263-266): *Well we, me and my husband always am checking something what’s new in diabetes or I know in US they have some research and they found one man was had transplant, pancreas transplant with… so waiting for the results, in time maybe maybe… always we are looking for new information what is or for the new devices or...*  OVERLAP WITH QUALITY OF SUPPORT AND MENTAL TOLL OF DIABETES | **Varying levels of information seeking**  While there was congruence with engaging in information seeking the frequency of this held a disparity. The adolescent commented on it as uncommon while the mother noted herself and her husband as keeping up to date with treatment options and research trials in the US. |
|  | **Impact on the Vocational Environment** | | |  |
| **Dyad** | **Subthemes** | **Adolescent** | **Parent** | **Dyadic Code/Summary** |
| 10 | **Vocational Performance** | Noted pushing through symptoms of a hypo in order to finish tests in school highlighting the burden of fluctuations on her mental performance (lines 145-153): *Well sometimes when I’m taking tests… Uhm… my sugar tends to drop…or…Whenever my sugar is low my vision starts to get blurry…And I can’t really write…I just have to push through it and… ignore it.* Noted some peer interest and curiosity about diabetes (lines 182-189): *They don’t mind it, sometimes they are interested in knowing like… I have this one friend…Where like… Okay, whata… what if… ‘What’s the highest its every been? What’s the lowest? What do you do if it’s low, what do you do if it’s high?.* Noted exclusion from PE when hypo but reported not minding this (lines 234-235): *Sometimes when I’m doing P.E at school… I might ehm…(pause)… I might my sugar might go low and I might have to sit out…But I don’t have any problem with that.* | Noted compared to her daughter she feels she cannot complain and feels parental responsibility and guilt when missing work for her own sickness (line 325): *So I don’t know how to say… I don’t have right to be sick (laugh).* Reported loss of her dream job in order to take care of her daughter noting child health as the only priority at that time for her entire family (lines 328-335): *I forgot about all my job all my… and just focused about her health and … me and my husband…Altogether just focused about her diabetes and… lot of information learning how to how to eat, how to eat healthy how can manage, she was just one years…She had just one years old so it’s ... and just leave all our jobs, our dreams, our that's focused on just her health.* | **Pushing through diabetes versus altering life for diabetes**  While the adolescent discussed having to work through BG fluctuations in school and the physical symptomatology that accompany this and affect her work, the mother noted a complete life change due to diabetes. The mother noted both herself and her husband left their dream jobs in order to care for their daughter. The mother highlighted the parental guilt at her daughter having been diagnosed as impacting her own likelihood to take a sick day and for her eventual career change to something with more flexibility for her daughter. |
|  | **Vocational Values** | Belief that school would be as important regardless of diabetes due to personal need for perfection (lines 164-165): *I think that I, if I even if I didn’t have diabetes it would still be important because…(pause)…Eh… I again have to make everything perfect.* Reported no affect of diabetes on academic goals. | Noted now while her job is still as important to her, it will never be her priority (line 339): *Yes, but it's not first.* | **Diabetes as secondary**  Across the dyad it was agreed that work/school are important regardless of having diabetes. While the daughter noted this with regards to her perfectionistic tendencies and ambition, the mother noted her child’s health will always take priority. |
|  | **Impact on the Home Environment** | | |  |
| **Dyad** | **Subthemes** | **Adolescent** | **Parent** | **Dyadic Code/Summary** |
| 10 | **Impact on Relationships** | Close bond with mother and no perception of diabetes having affected this (lines 199-200): *Even if I didn’t have diabetes I would still be very close to my mom.* Noted good relationship with father and sibling as well. | Good relationship with daughter trying to treat her as equal to her sibling and encourage her like any child (lines 379-387): *I'm doing all of that wh- what I can, to do for her…And it’s ehm first…she is like other child, like she's the same…Yeah… So I don't want to, she want, I don't want at Jessica, she feels some different, like another child. She's saying challenges she had. She has all rights and she can do all what she want…With her limits, of course and child limits.* Noted the difficulty in the sibling adjustment to the diagnosis surrounding the food restrictions (lines 398-403): *So then, so it was hard to eat something sweet and there was or cake or birthday or so. Yes, of course. It was hard was crying…Both sides explained her and we need for change our food we eat but if we want to help each other, we need just keep all, step by step.* | **Close bonds and sibling equality**  Across the dyad the close bond between the adolescent and her mother was reported. The mother went on to note that the sibling adjustment post diagnosis was difficult but that now the family treat it as if they all live with diabetes so try to retain fairness and all have the same diet. |
|  | **Quality of Support** | Varying levels of support available to her at home (line 224): *Well… my mom, yes, maybe my sister, and my dad I’m not so sure (laugh).* | Noted great level of hopeful support from her husband with regards to information seeking (lines 433-437): *Yes, I have support. Yes, I have support the support. I have my husband. He is very, very (laugh) Umm, I don't know how to say, but he is looking always for the news, for the new information about diabetes…Always checking the news or can ask from last by email about new device.*  OVERLAP WITH INFORMATION SEEKING | **Differing types of support**  While the adolescent reported varying levels of support from different family members, the mother noted her husband is a great emotional support as he always researches the newest technology and keeps the hope for the family. |
|  | **Impact on Family life** | Noted postponing or delaying chores in order to treat BG fluctuations (lines 212-214): *Sometimes my sugar eh… might go low during a chore I’m doing so sometimes I just leave the chore, take care of my sugar, and then do the do… the chore that I was asked to do after my sugar has gone better.* Noted the decreased financial demand in Ireland as compared to her home country (lines 242-243): *When we lived in Eastern European country*… it has been quite a problem but when we since we moved to Ireland it has been a lot better since then.* | Noted the drastic impact diabetes had on family life directly following the diagnosis with leaving careers to focus on diabetes management full time (lines 411-416): *Yes, I left my job…Once she got diagnosed because Umm… (pause) … I just 100% was looking for Jessica …So I left my job, my husband first, I think first half year just out of work we were…So we were, were focused about her sugar.* Noted differences in financial demands in home country (lines 466-470): *In Ireland it's like, it's much easier. Yes. In Eastern European Country* we had to buy all the equipment …Fingerpricker, Insulin, Uh doctors…appointments so but in Ireland we have the, the this device it’s free for us so?* | **Minor and Major impacts of diabetes on life**  Across the dyad both members commented on the lesser financial demands of diabetes in Ireland as compared to their home country. Additionally, the adolescent reported on small daily adjustments for her BGs while the mother reported major career changes and moving country as spurred by diabetes highlighting the parental responsibility for diabetes and family quality of life. |
|  | **Extended Family Relationships** | | |  |
| **Dyad** | **Subthemes** | **Adolescent** | **Parent** | **Dyadic Code/Summary** |
| **10** | **Extended family communication** | Noted no perceived affect of diabetes on interest or engagement with the wider family (line 261): *No, I was really I was always interested.* | Noted increased communication post-diagnosis due to familial concern and lack of awareness. Acknowledged the understanding has somewhat improved over the years (lines 495-501): *They were, they were worried and didn't understand how to calculate how to go- She can't eat something that's not normal or they can't understand why I I am …(pause) … ehm take the away the chocolate or chocolates or…or she needs count the carbohydrates…So they they think that's not it's not normal…But on time they understand, understand that that's this is the our way.* | **Post-diagnosis increase**  While the adolescent perceived no influence of diabetes on her communication with the wider family the mother highlighted the increase directly after diagnosis. This highlights how the daughter doesn’t remember life without diabetes, and the gradual growth from the wider family with regards to some diabetes awareness. |
|  | **Extended family quality of support** | Wider family support as a given from the relatives living in Ireland (line 271): *They’d definitely help me.* While also acknowledging the lack of diabetes management understanding and the probable need to educate them (lines 275-276): *They’d just tell me… They could just ask me what… what they should do and then they help me do it.* | Noted increased get-togethers with wider family post-diagnosis as they lacked awareness of the illness progression and a general fear of the unknown (lines 508-512): *They most interested…And they are worried about the, you know, the side effects…After long diabetes.* Noted no practical support available to her with regards to the diabetes (line 516): *Just emotional support…* | **Lack of true understanding**  There was congruence across the dyad about the lack of wider family understanding of diabetes. The adolescent noted while knowing she could ask for support, she would have to educate or explain what she needed. Similarly, the mother reported no available practical support but did acknowledge emotional support as available to her. |
|  | **Social Environment** | | |  |
| **Dyad** | **Subthemes** | **Adolescent** | **Parent** | **Dyadic Code/Summary** |
| 10 | **Impact on interest levels & participation** | Noted BG fluctuations as affecting hobby participation as reporting diabetes as non-negotiable in being the first priority (lines 292-294): *Sometimes when my sugars, when my glucose level goes high or low…(pause)…when I’m doing the hobby… it’s like yeah okay I have to take care of it first.* | Less time for hobbies since diagnosis noting a reprioritisation of her family over personal wants (lines 553-558): *Umm I I was more…free? before…Then I I understand I have the obligation…So just just like I put the values, what's most important for me, for me and for my family.* Noted mother as primary caregiver having the brunt of responsibilities placed on her and only with transition to self-management did activities with her spouse become more feasible (lines 583-586): *Before, when she was younger, it was hard because she depends of me 24 hours…Every year I try to learn, teach her* | **Diabetes as non-negotiable**  There was agreement across the dyad that diabetes management takes precedence over hobby engagement. The adolescent noted having to immediately tend to her BGs regardless of missing out on activities. Similarly, the mother noted her parental responsibility as the primary caregiver meant that she had to place her child’s health above her own wants and needs. |
|  | **Psychological Distress** | | |  |
| **Dyad** | **Subthemes** | **Adolescent** | **Parent** | **Dyadic Code/Summary** |
| 10 | **Mental toll of Diabetes** | Feeling like she has let her mother down when her management isn’t perfect (lines 338-340): *Sometimes when my sugar’s not really quite right… I just feel a bit …(pause)… ehm… ehm… what’s the word… I feel like I let my mom down because my sugar’s not… as good as it’s supposed to be.* Also reported increased worrying due to her diabetes. Noted some restricted and disordered eating patterns to achieve better BG levels due to feeling down on herself when they are out of range (lines 350-352): *Sometimes I feel like… ehm… I wake up… and I see that my sugar’s like… ehm… high like… okay what can I eat to make sure that my sugar goes low and to… Ehm… make me full… and sometimes… I just cannot do it so I just don’t eat in the morning.* | Noted the parental guilt she carries over miscalculating insulin doses (lines 621-623): *Yes, sometime when she's sick. And sugar, it's, I cannot understand why her sugar is high… I, I feel a bad Mom. Why? Because we calculated and I set set the basal. But sugar is still low and this is, can be, I'm very much emo-very emotional reaction I have.* Highlighted residual anger over why her daughter (lines 636-637): *Sometime yes. Why? Why? Why me? Why Jessica? …But no… (pause)… no answers…* Noted conversation with her daughter about hope for a cure, and the comparison with diabetes and other illnesses knowing that she can live a relatively normal life (lines 656-664): *Uh, we, she asked me about cure for diabetes and but she told me today, I know it's not good for the diabetes... I have to live the diabetes but maybe, maybe in time, maybe in time…But normal life you can have I know, somebody have, somebody has another problem, health problem…We have the diabetes, so we need to live with the diabetes, just take under control, just help her adjust, just more activity we don't, we don't have another choice.*  OVERLAP WITH INFORMATION SEEKING | **Dyadic guilt with diabetes**  There was congruence in the dyad about experiencing guilt or feeling like they were letting the other member down. This highlights the close bond they share, as well as the emotional burden of balancing the illness. Additionally, the adolescent noted some restricted eating to try and maintain her BGs within the desired range highlighting the extents to which behaviour is driven by this fear for complications and drive for perfection. However, the mother noted that despite the emotional toll of diabetes there is a shared understanding that her daughter can live a relatively normal life with good management.  OVERLAP WITH EMOTIONAL PERCEPTIONS AND IDENTITY, DIABETES VISIBILITY AND INFORMATION SEEKING |
|  | **Diabetes visibility** | Awareness of her diet and health as affecting her appearance overall (lines 364-357): *And I have to make sure that… I’m eating normally and sometimes I eat like more meat, I eat more vegetables, sometimes I eat more chocolate…But I feel like I wouldn’t look the same as I would if I didn’t have diabetes.* | No reported influence of diabetes on physical appearance. | **Health consciousness**  While the mother noted no impact of diabetes on her appearance, the adolescent highlighted her increased health consciousness and restricted diet as influencing her appearance.  OVERLAP WITH MENTAL TOLL OF DIABETES AND ATTITUDES TOWARDS HEALTHCARE |
|  | **Other** | Reported the need to highlight that while diagnosis is a difficult period, that overall diabetes is a manageable condition and eventually becomes part of daily life (lines 371-373): *Well, I guess when you have diabetes for a very long time, it’s a lot more… ehm…manageable and if you’ve been just diagnosed then like you don’t know what to do…(pause)…but it’ll be it’ll be okay, like about 3 years’ time you’ll be fine.* | N/A | **Adjustment as a gradual process**  While the mother had no additional comments the adolescent noted that diabetes does eventually become an integrated aspect of daily life. |
